# Total Synthesis of the Allenic Macrolide (+)-Archangiumide

**DOI:** 10.1021/jacs.3c13304

**Published:** 2024-01-19

**Authors:** Jack L. Sutro, Alois Fürstner

**Affiliations:** Max-Planck-Institut für Kohlenforschung, 45470 Mülheim/Ruhr, Germany

## Abstract

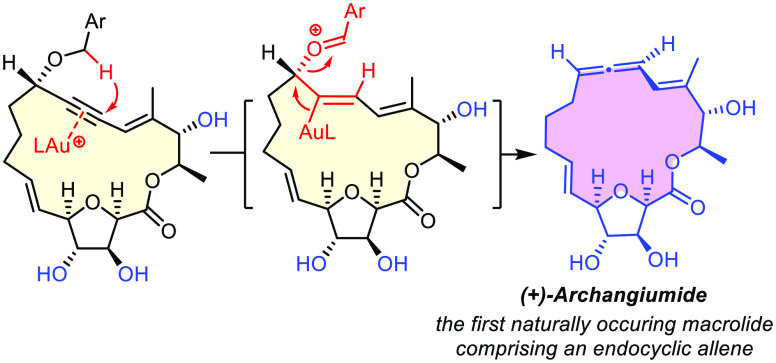

Archangiumide is the first known macrolide natural product comprising an endocyclic allene. For the ring strain that this linear substructure might entail, it was planned to unveil the allene at a very late stage of the projected total synthesis; in actual fact, this was achieved as the last step of the longest linear sequence by using an otherwise globally deprotected substrate. This unconventional timing was made possible by a gold catalyzed rearrangement of a macrocyclic propargyl benzyl ether derivative that uses a −PMB group as latent hydride source to unveil the signature cycloallene; the protecting group therefore gains a strategic role beyond its mere safeguarding function. Although the gold catalyzed reaction per se is stereoablative, the macrocyclic frame of the target was found to impose high selectivity and a stereoconvergent character on the transformation. The required substrate was formed by ring closing alkyne metathesis (RCAM) with the aid of a new air-stable molybdenum alkylidyne catalyst.

Allenic natural products are scarce overall,^1^ but examples comprising an allene as part of a macrocycle are exceedingly rare. Only a single type of plant-derived germacranolide had been assigned such a substructure^2^ before the macrolide archangiumide (**1**) was disclosed in 2021.^3^ This unique polyketide incorporates a stereogenic allene in a 17-membered lactone, as rigorously proven by X-ray diffraction (Scheme 1A).^3,4^ The myxobacterium *Archangium violaceum* SDU8 found to produce **1** was collected in Shangdong province, China, and had been singled out by combined genome mining and NMR-based metabolomic profiling as a potential source of secondary metabolites with little structural redundancy.

**Scheme 1:**
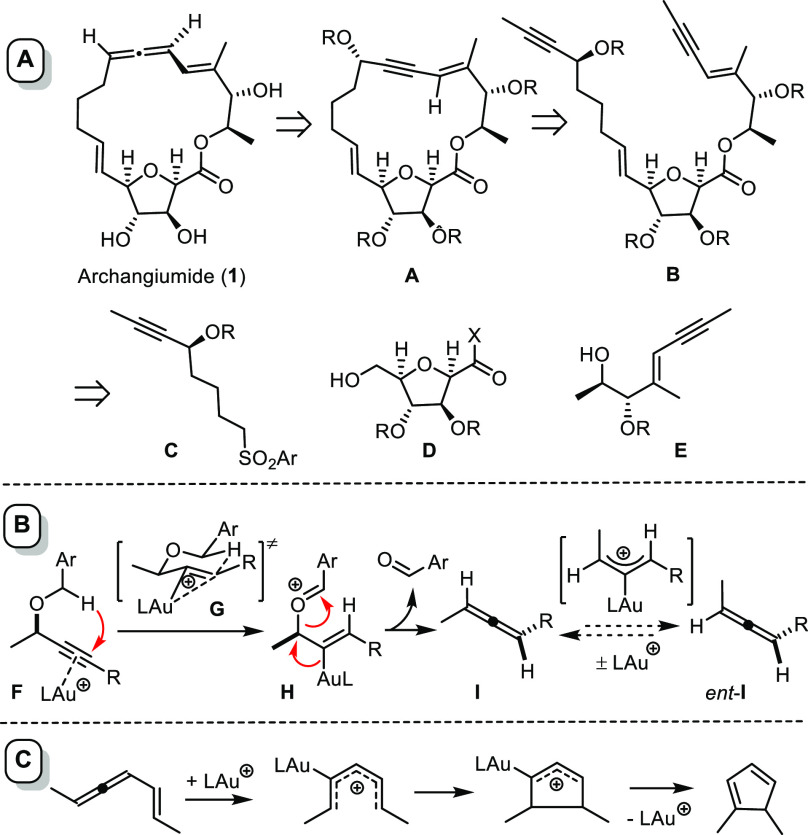
(A) Retrosynthetic Plan; (B) Projected Allene Formation by Gold Catalyzed Hydride Transfer; (C) Gold Catalyzed Cycloisomerization of Vinyl-allenes

While the discovery of **1** nicely confirmed this notion and represents the landmark of an essentially untapped sector of chemical space, the biological role of the compound remains obscure: **1** was assayed by the isolation team for antibacterial, antifungal, anticancer, antioxidant, and anti-inflammatory activity, but no significant effects were noticed.^3^ This puzzling aspect notwithstanding, archangiumide deserves attention as the prototype of a new class of secondary metabolites of microbial origin. For its linearity, an allene might well be expected to impose strain onto a macrocyclic scaffold, which, conversely, will enhance the already high reactivity of the cumulated double bonds.^5,6^ This seemed particularly true in the present case, where two additional *E*-configured alkenes and an annulated tetrahydrofuran ring cause extra rigidity and, hence, likely augment the synthetic challenge. From a strategic viewpoint, it therefore seemed prudent to unveil the allene entity at a very late stage of the projected total synthesis.^7^

We conjectured that the gold catalyzed rearrangement of propargyl benzyl ethers pioneered by Gagosz and co-workers might provide an adequate way to do so (Scheme 1B).^8^ By virtue of its high alkynophilicity,^9,10^ the π-acid catalyst almost certainly allows a polyunsaturated substrate of type **A** to be activated at the proper site;^11−15^ a kinetically favorable intramolecular 1,5-hydride transfer will ensue (**F** → **I**). The entropic and enthalpic gain upon release of benzaldehyde as a stable byproduct should drive the reaction forward, even if the targeted (cyclo)allene is potentially strained.

In contrast to these presumed chemical assets, the stereochemical outcome of the gold-catalyzed reaction was difficult to gauge, not least because the original report does not provide any pertinent information.^8,16,17^ Although a highly ordered six-membered transition state **G** can be envisaged that should result in chirality transfer from the propargylic center of substrate **F** to the chiral axis of product **I** upon (presumed) *anti*-elimination of an intermediate of type **H**, gold catalysts are known for their ability to racemize optically active allene derivatives; this process can be fast and facile.^18^ Yet, the literature also documents cases in which little or no loss of chiral information was observed on exposure of such derivatives to π-acids.^19^ Although a reliable forecast was therefore impossible, recourse to a chiral gold catalyst appeared to us as a potential last resort, should no other way be found to gain control over the stereochemical course of the reaction.^20^

Equally daunting was the fact that archangiumide is a vinylallene. On treatment with catalytic [LAu^+^], such compounds are known to convert into cyclopentadiene derivatives by electrocyclization via a presumed pentadienyl cation intermediate (Scheme 1C).^8,21^ In the present case, however, such a cycloisomerization could be disfavored by the confined macrocyclic envelope that might prevent a substrate of type **A** from adopting the conformation required for cyclization; moreover, the ensuing ring contraction seemed unfavorable. Beyond these plausibility arguments, there was no evidence available at the outset that the projected gold catalyzed reaction would stop at the vinylallene stage.

Although these potential issues concerned the very end game of the envisaged total synthesis, the strategic advantages of encoding the chiral allene as a macrocyclic propargyl alcohol derivative seemed to outweigh the risks. Alkynes are useful handles for fragment coupling purposes and cycloalkynes are well within reach of ring closing alkyne metathesis (RCAM).^22−24^ The required catalysts, most notably molybdenum alkylidynes endowed with (tripodal) silanolate ligands, hold the promise of being compatible with all functionalities present in a substrate of type **B**.^25−28^ They activate triple bonds but leave all kinds of olefins untouched, which is imperative when targeting a polyunsaturated compound such as **A**. Under the premise of rigorous chemical orthogonality,^29^ archangiumide (**1**) can be traced back to three very manageable building blocks, **C**–**E**, whereby fragment **D** corresponds to protected d-chitaric acid.

A suitable compound of this type was available on gram scale from commercial d-mannono-1,4-lactone (Scheme 2).^30^ The derived acetal **2** is known to react selectively at the C2-OH position with Tf_2_O/pyridine. Treatment of the resulting sulfonate ester **3** with MeOH in acidic medium entailed concomitant lactone opening, acetal cleavage, and cyclization of the resulting polyol to furnish compound **4**.^30^ This product was then elaborated into aldehyde **6** in readiness for fragment coupling. It is important to note, however, that **6** proved to be unstable to storage and had to be used immediately upon preparation.

**Scheme 2:**
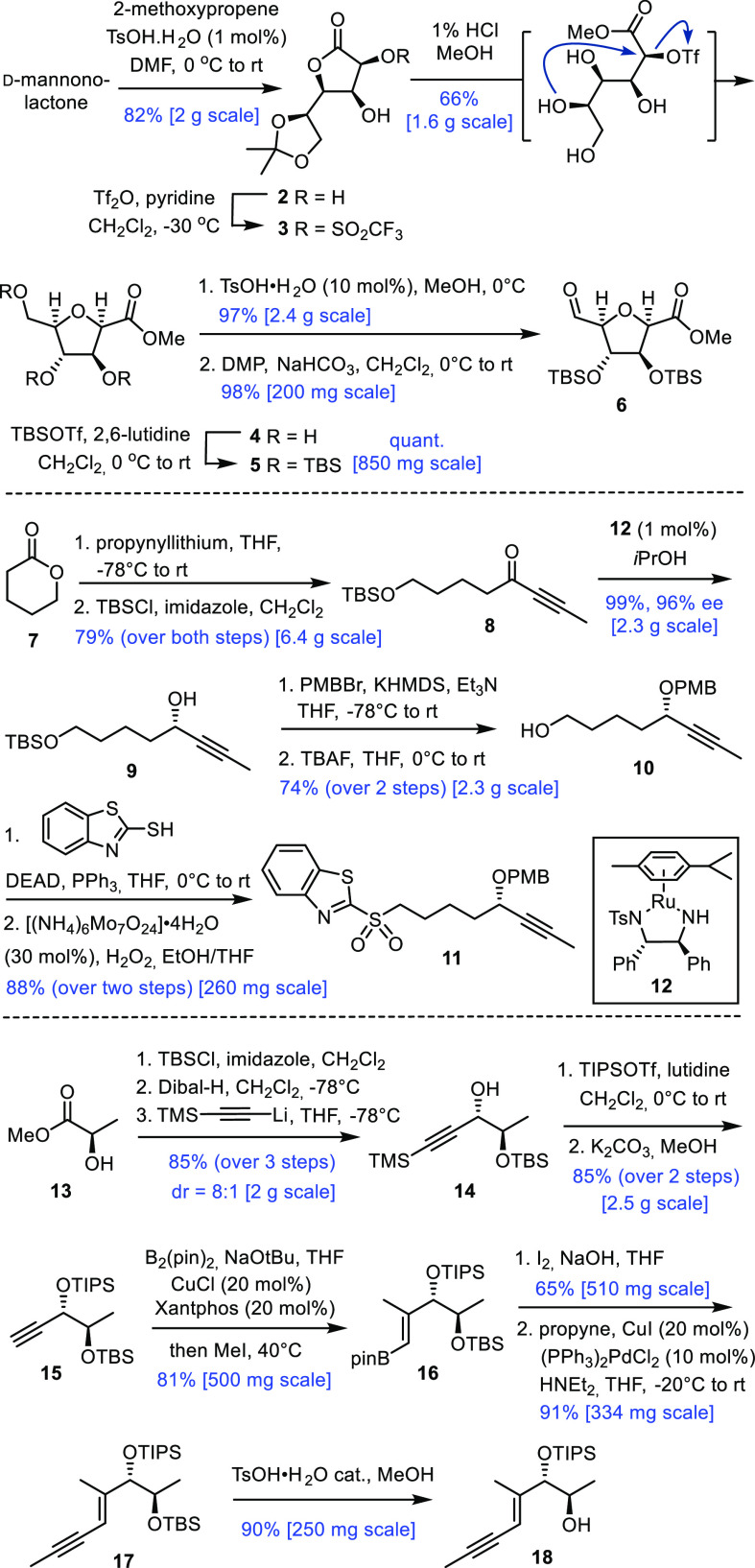
Preparation of the Building Blocks

Sulfone **11** was secured by addition of propynyllithium to δ-valerolactone (**7**) in THF at low temperature to give the corresponding alkynylketone, which was isolated only after silylation of the primary −OH group.^31^ A Noyori transfer hydrogenation ensured the asymmetric reduction of the carbonyl group of **8** with excellent ee and near quantitative chemical yield;^32^ the plan was to engage this chiral propargylic center in the late-stage allene formation. We chose to protect the alcohol as −PMB (rather than parent benzyl) ether based on the mechanistic rationale that an electron-rich benzyl group might facilitate the gold catalyzed hydride transfer **F** → **I** via stabilization of the presumed oxocarbenium intermediate **H**, even though the literature does not mention any substituent effect.^8^ Compound **10** was then readily transformed into sulfone **11**. All it took to secure the antipodal building block *ent*-**11** was to use the enantiomeric Noyori catalyst *ent*-**12** in the reduction step and follow the sequence from there on (for details, see the Supporting Information). With both antipodes in hand, it should be possible to clarify the so far unknown stereochemical course of the projected gold catalyzed allene formation.

For the preparation of the third building block, methyl d-lactate (**13**) was O-silylated and the resulting ester reduced with DIBAL-H; lithiated TMS-acetylene was then added to the aldehyde thus formed. This reaction was diastereoselective (*anti*/*syn* ≈ 8:1), high yielding, and scalable.^33^ Orthogonal protection of the newly formed alcohol in **14** followed by cleavage of the C-TMS group gave **15**. Attempted carboalumination **15** (or partly deprotected analogues) followed by an iodine quench basically met with failure; gratifyingly though, a copper catalyzed net carboboration constituted a convenient and robust alternative that provided access to **16** in good yield and excellent selectivity.^34,35^ Subsequent boron/iodine exchange followed by a Sonogashira coupling^36^ with excess propyne at low temperature secured enyne **17** in isomerically pure form. Treatment with tosic acid in MeOH then gave the required alcohol derivative **18**.

The assembly phase commenced with a modified Julia-type olefination of freshly prepared aldehyde **6** with sulfone **11** (Scheme 3). Despite the excellent track record of this transformation,^37^ this step required careful optimization. Specifically, LDA (rather than the more commonly used alkali hexamethyldisilazides) turned out to be the base of choice; the deprotonation of **11** had to be carried out in THF at −100 °C; a precooled (−65 °C) solution of the aldehyde in DMF was then rapidly added to the lithiated sulfone, and the resulting mixture was slowly warmed. Any excess base had to be strictly avoided and the temperature had to be rigorously controlled to prevent notable decomposition. Under these conditions, however, alkene **19** was formed in well reproducible 75% yield with a favorable isomer ratio (*E*/*Z* ≈ 6:1); the material was best purified after the saponification of the methyl ester. The resulting acid was then linked to alcohol **18** via the Yamaguchi protocol.^38^

**Scheme 3:**
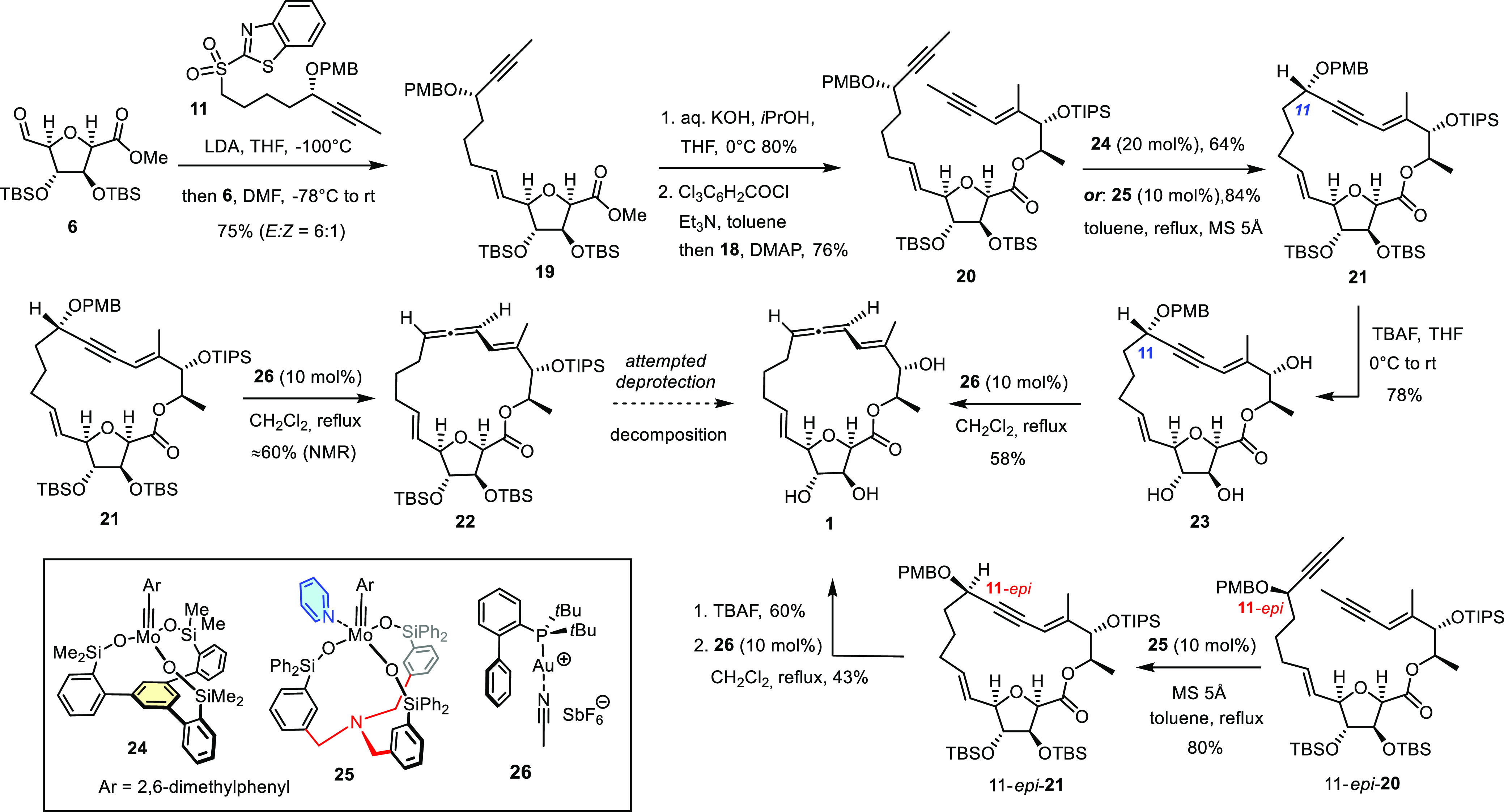
Completion of the Total Synthesis

In line with our expectations, macrocyclization of diyne **20** by RCAM worked well with the aid of the latest alkyne metathesis catalysts developed in our laboratory. The reaction proceeded within 30 min when the highly active “canopy catalyst” **24** was used.^26,27,39,40^ Although complex **25** reacted more slowly (≈2 h), this pyridine adduct has the distinct advantage that it can be handled and weighed in air;^28^ it is storable in a desiccator outside a glovebox for many months without noticeable decomposition. Upon dissolution in toluene, the pyridine ligand dissociates spontaneously and the active molybdenum alkylidyne is released without the need for any extra physical or chemical stimulus.^28^ For the ease of handling in combination with a broad functional group compatibility, **25** is deemed to mark an important advance in the field of alkyne metathesis in general.^22^ As the current example illustrates, **24** and **25** both selectively activated the triple bonds in **20**, whereas the (conjugated) alkenes and all polar substituents, including the critically important stereogenic benzylic ether center, remained intact.

With cycloalkyne **21** in hand,^41^ the stage was set to test the gold catalyzed allene formation. On treatment with complex **26** (10 mol %)^42^ in CH_2_Cl_2_ at reflux temperature, a slow but clean formation of a single allene isomer **22** (δ_C_ = 209 ppm) was observed by NMR. Rather than trying to assign its stereostructure by spectroscopic means, global deprotection and comparison of the resulting product with archangiumide seemed to provide a faster and more conclusive answer, since the stereostructure of **1** had been unambiguously established by X-ray diffraction.^3^ Unfortunately, however, all our attempts to cleave the three silyl ethers in **22** resulted in rapid decomposition.^43^

We assumed that the increase in ring strain upon endocyclic allene formation might render this compound so sensitive. This notion was supported by the fact that the reverse order of events ultimately proved successful. Thus, cycloalkyne **21** was first treated with excess TBAF in THF; the resulting triol **23** was then exposed to **26** under the conditions described above to furnish an allene product and an equivalent of *p*-methoxybenzaldehyde as judged by ^1^H NMR. After purification of the crude material by flash chromatography, the spectral and analytical data of the synthetic sample matched those of archangiumide (**1**) in all respects.^3^ The goal was reached in 13 steps (longest linear sequence) with an overall yield of ≈3.8%; no indications argue against scale-up of the endgame, if deemed necessary.

The conceptual implications of performing the gold catalyzed reaction as the ultimate step of the entire sequence deserve comment. Since the chosen protecting group serves as a latent hydride source needed to unveil the signature cycloallene substructure of the target, the final ether cleavage gains a *strategic* role. The fact that the longest linear sequence was terminated by the actual key step, which followed only after an otherwise global deprotection, is highly uncommon in natural product synthesis;^44^ this unorthodox orchestration arguably marks an underappreciated opportunity provided by π-acid catalysis.

The exclusive formation of **1** from **23** seems to imply strict chirality transfer in the gold catalyzed step, but this conclusion could be premature. Therefore, 11-*epi*-**20** was assembled by following the same route but using the antipodal sulfone fragment *ent*-**11** (for details, see the Supporting Information). Once again, RCAM proceeded cleanly with the aid of the new pyridine adduct **25**.^28^ Cleavage of the silyl groups off the resulting cycloalkyne 11-*epi*-**21** followed by the gold catalyzed deprotective allene formation also furnished a single discrete product, although in lower yield and after a longer reaction time. Somewhat surprisingly, the recorded data once again perfectly matched those of archangiumide (**1**)^3^ and were identical to those of the sample derived from **21**.

This perplexing stereoconvergence compelled us to subject the acyclic substrate **27** (96% ee), derived from one of intermediates passed through during the preparation of the sulfone building block, to the same reaction conditions (Scheme 4). In this case, the resulting allene **28** was racemic, which shows that the gold catalyzed reaction per se is stereoablative and does not transmit stereochemical information from the propargyl benzyl ether center to the incipient chiral axis of the resulting allene. The fact that archangiumide (**1**) was obtained as a single diastereomer, independent of whether **23** or 11-*epi*-**23** was used as the substrate, is therefore attributed to thermodynamic control;^45^ it is the macrobicyclic framework decorated with six stereogenic centers and two *E*-alkenes that determines the stereochemical course.^46^ This conclusion may even bear implications for the biosynthesis of the natural product.^47^

**Scheme 4:**
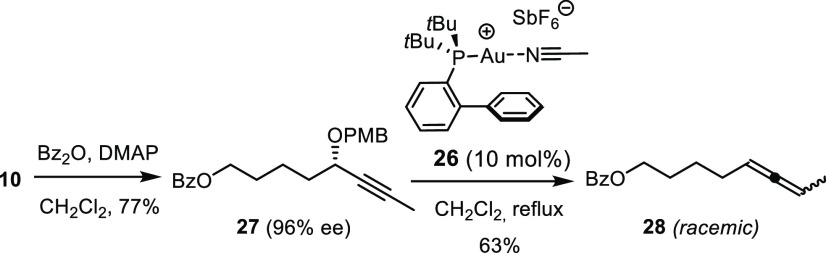
Control Experiment

## Supporting Information Available

Experimental section including characterization data and NMR spectra of new compounds (PDF)
