# Supplementary material for: Total Synthesis of the Allenic Macrolide (+)-Archangiumide
Source: J Am Chem Soc. 2024 Jan 19;146(4):2345–50. doi: 10.1021/jacs.3c13304 (PMC10835656; doi:10.1021/jacs.3c13304)
Supplement: Supplementary file 1 — ja3c13304_si_001.pdf [file ja3c13304_si_001.pdf]

SUPPORTING INFORMATION

Total Synthesis of the Allenic Macrolide (+)-Archangiumide

Jack L. Sutro and Alois Fürstner*

Max-Planck-Institut für Kohlenforschung, 45470 Mülheim-an-der-Ruhr, Germany

Email: fuerstner@kofo.mpg.de

Table of Contents

General Information S1

Preparative Procedures and Characterization Data S2

Building Blocks S2

Control Experiment S13

Fragment Coupling and Completion of the Total Synthesis S21

NMR Spectra of New Compounds S35

References S71

General Information

Unless stated otherwise, all reactions were carried out in flame-dried glassware using anhydrous solvents under an argon atmosphere. The following solvents were purified by distillation over the indicated drying agents and were transferred under an argon atmosphere: THF (magnesium/anthracene); diisopropylamine, 2,6-lutidine, pyridine, *tert*-butyl methyl ether, dichloromethane (CaH_2); toluene (sodium tetraethylaluminate); MeOH, EtOH, *i*PrOH (Mg; stored over 3 Å MS). DMSO, DMF, NEt_3, and pyridine were dried by an adsorption solvent purification system based on molecular sieves. 5 Å Molecular sieves (MS) were activated at 150 °C for 24 h under high vacuum (1×10^{-3} mbar), and stored and transferred under argon.

Thin layer chromatography (TLC): Macherey-Nagel precoated plates (POLYGRAM®SIL/UV254); Visualization by UV light (254 nm) or by staining with solutions of *p*-anisaldeyhde or $KMnO_4$.

Flash chromatography: Merck silica gel 60 (40-63 µm or 15-40 µm) using pre-distilled or HPLC grade solvents.

NMR: Spectra were recorded on Bruker AV 400, AVIII 600 or AVneo 600 spectrometers in the solvents indicated; chemical shifts (δ) are given in ppm relative to tetramethylsilane (TMS) and coupling constants (*J*) are given in Hz. The (residual) solvent signals were used as references, and were themselves referenced to known values relative to TMS ($CDCl_3$: δ_C = 77.16 ppm; residual $CHCl_3$ in $CDCl_3$: δ_H = 7.26 ppm; [D_6]-DMSO: δ_C = 39.52 ppm; residual D_2HSOCD_3 in [D_6]-DMSO: δ_H = 2.50 ppm; CD_3OD: δC = 49.00 ppm, residual CD_2HOD in CD_3OD: δ_H = 3.31 ppm; CD_3CN: δC = 118.26 ppm, residual CD_2HCN in CD_3CN: δ_H = 1.94 ppm). All spectra were recorded at 25 °C. Multiplicities are reported as they appear in the spectrum, rather than as would be expected *a priori* (i.e. a resonance which would be predicted to be a double doublet but has two identical coupling constants is reported as a triplet). Peak multiplicities are indicated by the following abbreviations: s: singlet, d: doublet, t: triplet, q: quartet, p: pentet, h: hextet, hept: heptet, m: multiplet, dd: double doublet, dt: double triplet, and so on; "br" indicates a broad peak, where the width is large enough to eclipse small coupling constants, which might otherwise be expected. ^{13}C NMR spectra were recorded with ^{1}H-decoupling, and the values of the chemical shifts are rounded to one decimal place. Two decimal places are given in cases where there are two or more ^{13}C resonances which have the same value when rounded to one decimal place. Signal assignments were established using HSQC, HMBC, COSY, and NOESY.

IR: Spectra were recorded on an Alpha Platinum ATR instrument (Bruker); wavenumbers ($\tilde{\nu}$) are reported in cm^{-1}.

MS (ESI-MS): Finnigan MAT 8200 (70 eV), ESI-MS: ESQ3000 (Bruker), accurate mass determinations: Bruker APEX III FTMS (7 T magnet) or Mat 95 (Finnigan).

Optical rotations ($[\alpha]_D^{20}$) were measured with an A-Krüss Optronic Model P8000-t polarimeter at a wavelength of 589 nm. Optical rotations are not reported for compounds of lower than 90% diastereomeric purity.

The data for all literature-known compounds were consistent with those previously reported.

Unless stated otherwise, all compounds were commercially available (Alfa Aesar, Sigma Aldrich, TCI, Strem Chemicals, ChemPUR) and used as received. The alkyne metathesis catalyst was prepared by other members of the Fürstner group in accordance with the published literature procedure.[1]

Preparative Procedures and Characterization Data

Building Blocks

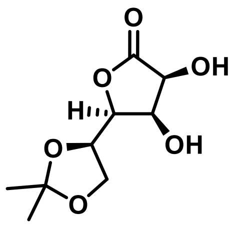

Compound 2.[7,8] D-Mannono-1,4-lactone (2.00 g, 11.2 mmol) was dissolved in dimethylformamide (8 mL) and the resulting solution was cooled to 0 °C in an ice bath. *Para*-toluenesulfonic acid monohydrate (20 mg, 0.11 mmol) was added, followed by 2-methoxypropene (1.45 mL, 15.1 mmol), which was added dropwise over 3 min. The resulting colorless solution was allowed to warm to 20 °C and was stirred for 20 h. Solid potassium carbonate (1 g) was added, and the suspension was filtered through a pad of Celite™, eluting with ethyl acetate. The combined filtrates were concentrated under reduced pressure. The residue was purified *via* flash chromatography eluting with ethyl acetate/hexane (50→80% ethyl acetate by volume) to give the title compound as a white solid (2.07 g, 82% yield). m.p. = 138.0-138.2 °C (recrystallized from hexane/ethyl acetate; fine needles) (lit:[7,8] 136-139 °C). $[\alpha]_D^{20}$= 49.0 (c = 1.37, MeOH) (lit.:[7] $[\alpha]_D^{21}$= 58.5, c = 1.00, H_2O). ^1H NMR (400 MHz, CD_3CN): δ 4.46 (br d, *J* = 4.4 Hz, 1H), 4.41 – 4.23 (m, 3H), 4.08 (dd, *J* = 8.8, 6.1 Hz, 1H), 3.91 (dd, *J* = 8.8, 4.8 Hz, 1H), 3.64 (br

s, 1H), 1.38 (s, 3H), 1.32 (s, 3H). ^{13}C NMR (101 MHz, CD_3CN): δ 176.1, 109.7, 80.0, 73.5, 71.4, 70.0, 66.6, 26.9, 25.3. IR (film): $\tilde{\nu}$ 3423 (br), 2988, 2938, 1783, 1375, 1257, 1213, 1190, 1142, 1067, 841 cm^{-1}. HRMS (ESI$^+$) calcd. for $C_9H_{14}O_6Na$ [M+Na]$^+$: 241.06826; found: 241.06810.

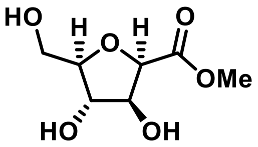

Compound 4.[7] Compound **2** (1.61 g, 7.38 mmol) was dissolved in pyridine (7.5 mL) and dichloromethane (65 mL), and the resulting solution was cooled to –30 °C. Triflic anhydride (1.60 mL, 9.51 mmol) was added dropwise, and the resulting mixture was stirred at –30 °C for 1 h. Water (0.75 mL) was added, and the mixture was allowed to warm to ambient temperature whilst being stirred vigorously. The mixture was then filtered through a short plug of silica topped with anhydrous magnesium sulfate, eluting with ethyl acetate. Toluene (20 mL) was added to the filtrate, and the solution was concentrated under reduced pressure to yield a crimson oil.

This product was dissolved in methanol (74 mL) and concentrated hydrochloric acid (0.74 mL) was added dropwise. The resulting yellow solution was stirred at ambient temperature for 24 h, over which time the color of the solution faded significantly. After this time had elapsed, sodium hydrogen carbonate (5 g) was added, and the resulting mixture was stirred rapidly at ambient temperature for 2 h before it was filtered through Celite™ (eluting with methanol). The filtrate was concentrated under reduced pressure, and the residue was adsorbed onto Celite™ and purified *via* flash chromatography, eluting with methanol/dichloromethane (0→5% methanol by volume), to give the title compound as a yellow oil (936 mg, 66% yield). $[\alpha]_D^{20}$= 24.3 (c = 1.25, MeOH) (lit.:[7] $[\alpha]_D^{21}$= 27.8, c = 1.00, MeOH). ^{1}H NMR (400 MHz, MeOD): δ 4.67 (d, *J* = 4.3 Hz, 1H), 4.22 (dd, *J* = 4.3, 1.9 Hz, 1H), 4.04 (dd, *J* = 2.7, 1.9 Hz, 1H), 3.92 (td, *J* = 4.6, 2.7 Hz, 1H), 3.78 (s, 3H), 3.74 (d, *J* = 4.6 Hz, 2H). ^{13}C NMR (101 MHz, MeOD): δ 172.3, 88.6, 82.3, 79.5, 79.0, 63.2, 52.4. IR (film): $\tilde{\nu}$ 3364 (br), 2952, 2928, 2857, 1739, 1514, 1462, 1441, 1362, 1326, 1250, 1081, 837, 777 cm^{-1}. HRMS (ESI$^+$) calcd. for $C_7H_{12}O_6Na$ [M+Na]$^+$: 215.05261; found: 215.05287.

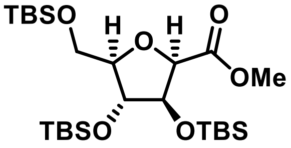

Compound 5.[7] Compound **4** (850 mg, 4.42 mmol) was dissolved in dichloromethane (44 mL) and 2,6-lutidine (5.12 mL, 44.0 mmol) and the resulting solution was cooled to –78 °C. TBSOTf (6.01 mL, 26.5 mmol) was added dropwise, and the resulting colorless mixture was allowed to warm to ambient temperature over 1 h. Saturated aqueous sodium bicarbonate (50 mL) was added, and the mixture was diluted with water (50 mL) and dichloromethane (50 mL). The organic

layer was separated, and the aqueous phase was extracted three times with ethyl acetate (50 mL). The combined organic extracts were washed with brine, dried over anhydrous magnesium sulfate, and filtered. The filtrate was concentrated under reduced pressure, and the residue was purified *via* flash chromatography eluting with ethyl acetate/hexane (0→5% ethyl acetate by volume) to give the title compound as a colorless oil (2.36 g, quant.). $[\alpha]_D^{20}$= 9.5 (c = 0.25, $CHCl_3$) (lit.:[7] $[\alpha]_D^{21}$= 13.6, c = 1.00, $CHCl_3$). ^{1}H NMR (400 MHz, $CDCl_3$): δ 4.70 (d, *J* = 3.4 Hz, 1H), 4.22 – 4.19 (br d, *J* = 3.4 Hz, 1H), 4.17 (br s, 1H), 3.93 (dd, *J* = 10.1, 5.5 Hz, 1H), 3.83 (dd, *J* = 9.9, 5.5 Hz, 1H), 3.73 (s, 3H), 3.70 (t, *J* = 10 Hz, 1H), 0.89 (s, 9H), 0.88 (s, 9H), 0.85 (s, 9H), 0.10 (s, 3H), 0.10 (s, 3H), 0.10 (s, 3H), 0.05 (s, 3H), 0.04 (s, 6H). ^{13}C NMR (101 MHz, $CDCl_3$): δ 170.0, 88.5, 82.0, 80.3, 78.5, 63.4, 51.9, 26.1, 25.8, 25.7, 18.4, 18.0, 17.9, –4.4 (2C), –4.6, –5.2, –5.28, –5.34. IR (film): $\tilde{\nu}$ 2953, 2930, 2858, 1737, 1472, 1463, 1254, 1097, 1074, 883, 774 cm^{-1}. HRMS (ESI$^+$) calcd. for $C_{25}H_{54}O_6Si_3Na$ [M+Na]$^+$: 557.31205; found: 557.31154.

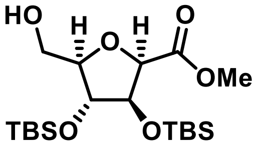

Compound S1. Compound **5** (2.36 g, 4.42 mmol) was dissolved in methanol (45 mL) and the resulting solution was cooled to 0 °C. *para*-Toluenesulfonic acid monohydrate (86 mg, 0.45 mmol) was added and the mixture was stirred at 0 °C for 2.5 h. Sodium bicarbonate (0.5 g) was added and stirring continued for 30 min while the mixture was allowed to warm to ambient temperature. The mixture was filtered through Celite™, eluting with methanol. The filtrate was concentrated under reduced pressure, and the residue was purified *via* flash chromatography eluting with ethyl acetate/hexane (5→20% ethyl acetate by volume) to give the title compound as a colorless oil (1.80 g, 97% yield). $[\alpha]_D^{20}$= 3.8 (c = 0.85, $CHCl_3$). ^{1}H NMR (400 MHz, $CDCl_3$): δ 4.66 (d, *J* = 4.4 Hz, 1H), 4.27 (dd, *J* = 4.4, 2.4 Hz, 1H), 4.09 (t, *J* = 2.4 Hz, 1H), 3.99 (td, *J* = 4.3, 2.4 Hz, 1H), 3.79 – 3.76 (m, 2H), 3.75 (s, 3H), 2.88 (t, *J* = 6.0 Hz, 1H), 0.89 (s, 9H), 0.86 (s, 9H), 0.11 (s, 3H), 0.10 (s, 3H), 0.09 (s, 3H), 0.07 (s, 3H). ^{13}C NMR (101 MHz, $CDCl_3$): δ 170.7, 88.0, 80.9, 80.0, 77.9, 62.2, 52.2, 25.8, 25.7, 18.0, 17.9, –4.3, –4.47, –4.48, –5.1. IR (film): $\tilde{\nu}$ 3400 (br), 2953, 2930, 2858, 1738, 1256, 1217, 1132, 1096, 931, 836, 777 cm^{-1}. HRMS (ESI$^+$) calcd. for $C_{19}H_{40}O_6Si_2Na$ [M+Na]$^+$: 443.22556; found: 443.22567.

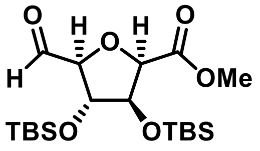

Compound 6. Compound **S1** (200 mg, 0.475 mmol) was dissolved in dichloromethane (3 mL) and sodium bicarbonate (395 mg, 4.75 mmol) was added. A solution of Dess-Martin periodinane (403 mg, 0.950 mmol) in dichloromethane (2 mL) was added, and the resulting suspension was stirred at

ambient temperature for 2 h. Saturated aqueous sodium bicarbonate (2 mL) was added followed by saturated aqueous sodium thiosulfate (2 mL), and the reaction mixture was stirred for a further 10 min. The reaction was diluted with water (5 mL) and dichloromethane (5 mL), the organic layer was separated, and the aqueous phase was extracted three times with ethyl acetate (10 mL). The combined organic extracts were washed with brine, dried over anhydrous magnesium sulfate, and filtered. The filtrate was concentrated under reduced pressure, and the residue was found to be pure enough for direct use in the next reaction (195 mg, 98% yield). $[\alpha]_D^{20}$= –17.1 (c = 1.38, $CHCl_3$). ^1H NMR (400 MHz, $CDCl_3$): δ 9.69 (d, *J* = 1.1 Hz, 1H), 4.88 (d, *J* = 3.0 Hz, 1H), 4.23 (dd, *J* = 3.0, 1.4 Hz, 1H), 4.21 (d, *J* = 1.1 Hz, 1H), 4.20 (d, *J* = 1.4 Hz, 1H), 3.79 (s, 3H), 0.90 (s, 9H), 0.81 (s, 9H), 0.14 (s, 3H), 0.13 (s, 3H), 0.04 (s, 3H), 0.03 (s, 3H). ^{13}C NMR (101 MHz, $CDCl_3$): δ 203.3, 169.5, 91.0, 82.8, 82.0, 78.0, 52.2, 25.8, 25.5, 18.0, 17.8, –4.5, –4.6, –4.7, –5.3. IR (film): $\tilde{\nu}$ 2953, 2931, 2859, 1773, 1732, 1462, 1257, 1204, 1125, 1099, 1056, 929, 837, 810, 778 cm^{-1}. HRMS (ESI^+) calcd. for $C_{19}H_{38}O_6Si_2Na$ $[M+Na]^+$: 441.20992; found: 441.21008.

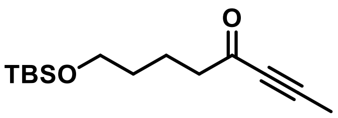

Compound 8. According to the modified procedure of Chabala and Vincent,[2] liquid propyne (stored in a Schlenk at –78 °C, 5.00 mL, excess) was dissolved in THF (100 mL) and the mixture was stirred at –78 °C. *n*-Butyllithium (1.6 M in hexanes, 22.0 mL, 35.2 mmol) was added, and the solution was warmed to ambient temperature and stirred for 30 min, during which time a white suspension formed. The suspension was cooled to –78 °C before δ-valerolactone (**7**) (3.00 mL, 32.3 mmol) was added dropwise, and the reaction was subsequently warmed to ambient temperature and stirred for 3 h. During this time, the suspended solid dissolved into the solution and a deep yellow color developed. Saturated aqueous ammonium chloride (100 mL) was then added, and the reaction was diluted with water (200 mL) and ethyl acetate (200 mL). The organic layer was separated, and the aqueous phase was extracted three times with ethyl acetate (200 mL). The combined organic extracts were washed with brine (200 mL), dried over anhydrous magnesium sulfate, and filtered. The filtrate was concentrated under reduced pressure, and the crude residue was dissolved in dichloromethane (150 mL). Imidazole (4.43 g, 65.0 mmol) was added, followed by TBSCl (7.31 g, 49.0 mmol). The mixture was stirred at ambient temperature for 1 h, during which time a suspension formed. Saturated aqueous sodium bicarbonate (100 mL) was added, and the reaction was diluted with water (100 mL) and dichloromethane (150 mL). The organic layer was separated, and the aqueous phase was

extracted three times with dichloromethane (150 mL). The combined organic extracts were washed with brine (150 mL), dried over anhydrous magnesium sulfate, and filtered. The filtrate was concentrated under reduced pressure, and the residue was purified *via* flash chromatography eluting with ethyl acetate/hexane (0→10% ethyl acetate by volume) to give the title compound as a yellow oil (6.44 g, 79% yield). ^{1}H NMR (400 MHz, $CDCl_3$): δ 3.61 (t, *J* = 6.3 Hz, 2H), 2.54 (t, *J* = 7.4 Hz, 2H), 2.01 (s, 3H), 1.76 – 1.67 (m, 2H), 1.57 – 1.48 (m, 2H), 0.89 (s, 9H), 0.04 (s, 6H). ^{13}C NMR (101 MHz, $CDCl_3$): δ 188.4, 90.1, 80.3, 62.8, 45.3, 32.1, 26.1, 20.7, 18.5, 4.2, –5.2. IR (film): $\tilde{\nu}$ 2953, 2929, 2857, 2220, 1674, 1472, 1462, 1252, 1166, 1102, 1006, 980, 835, 775 cm^{-1}; HRMS (ESI^+) calcd. for $C_{14}H_{26}O_2SiNa$ $[M+Na]^+$: 277.15943; found: 277.15936.

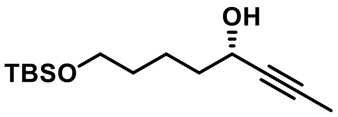

Activation of the Noyori Catalyst (*S,S*-12).[3] RuCl(*p*-cymene)[(*S,S*)-Ts-DPEN] (318 mg, 0.50 mmol) was dissolved in dichloromethane (5 mL). A solution of potassium hydroxide (281 mg, 5.00 mmol) in water (1 M in KOH, 5 mL) was added, and the biphasic mixture was stirred rapidly for 10 min, during the course of which a deep mauve color developed. The mixture was transferred to a separating funnel, the dichloromethane layer was separated, and the aqueous layer was extracted with dichloromethane. The combined organic extracts were washed with water, dried over calcium hydride, and filtered into a Schlenk flask. The filtrate was concentrated under reduced pressure to give a deep violet residue. This residue was repeatedly washed with dry pentane followed by concentration to remove residual volatile organic compounds, giving the activated catalyst as a deep violet powder (230 mg, 67% yield).

Compound (*S*)-9. According to the modified procedure of Noyori *et al.,*[4] compound **8** (2.30 g, 9.04 mmol) was dissolved in isopropanol (90 mL) and the mixture stirred at ambient temperature. (*S,S*)-**12** (activated as above, 54 mg, 0.09 mmol) was dissolved in dichloromethane (1 mL) and the solution injected into the mixture to give a pale orange solution. Stirring was continued at ambient temperature for 12 h. The reaction mixture was concentrated under reduced pressure, and the residue was purified *via* flash chromatography eluting with ethyl acetate/hexane (0→25% ethyl acetate by volume) to give the title compound as a colorless oil (2.29 g, 99% yield). $[\alpha]_D^{20}$= 8.7 (c = 0.50, $CHCl_3$). ^{1}H NMR (400 MHz, $CDCl_3$): δ 4.32 (tq, *J* = 6.7, 2.1 Hz, 1H), 3.62 (t, *J* = 6.3 Hz, 2H), 1.83 (d, *J* = 2.1 Hz, 3H), 1.73 – 1.61 (m, 2H), 1.59 – 1.37 (m, 4H), 0.89 (s, 9H), 0.04 (s, 6H). ^{13}C NMR (101 MHz, $CDCl_3$): δ 81.1,

80.5, 63.2, 62.8, 38.0, 32.5, 26.1, 21.7, 18.5, 3.7, –5.1. IR (film): $\tilde{\nu}$ 3300 (br), 2929, 2886, 2857, 1472, 1254, 1097, 834, 774 cm^{-1}. HRMS (ESI$^+$) calcd. for $C_{14}H_{28}O_2SiNa$ [M+Na]$^+$: 279.17508; found: 279.17494.

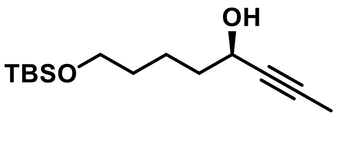

Compound (*R*)-9. Prepared analogously using the activated antipodal Noyori catalyst (*R,R*)-**12**. The title compound was isolated as a colorless oil (2.98 g, 99% yield). $[\alpha]_D^{20}$ = –7.2 (c = 0.50, CHCl$_3$). NMR, MS, and IR data matched those obtained for compound **(*S*)-9**.

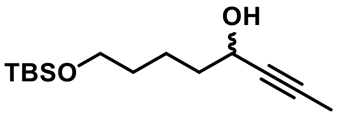

Compound (±)-9. Compound **8** (15 mg, 0.059 mmol) was dissolved in methanol (1 mL) and the resulting solution was cooled to 0 °C. Cerium(III) chloride heptahydrate (50 mg, 0.13 mmol) was added, followed by sodium borohydride (5.0 mg, 0.13 mmol). Moderate effervescence was observed upon the addition of the latter reagent. The resulting solution was stirred at 0 °C for 10 min before saturated aqueous ammonium chloride (1 mL) was added, and the reaction was diluted with water (5 mL) and ethyl acetate (5 mL). The organic layer was separated, and the aqueous layer was extracted three times with ethyl acetate (10 mL). The combined organic extracts were washed with brine, dried over anhydrous magnesium sulfate, and filtered. The filtrate was concentrated under reduced pressure, and the residue was purified *via* flash column chromatography eluting with ethyl acetate/hexane (0→25% ethyl acetate by volume) to give the title compound as a colorless oil (15 mg, 99% yield). NMR, MS, and IR data matched those obtained for compounds **(*S*)-9** and **(*R*)-9**.

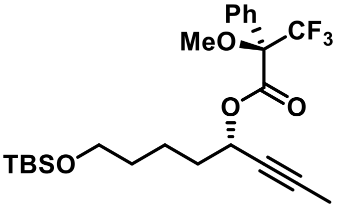

Compound S2 ((*R*)-Mosher's ester): (*S*)-Mosher's acid chloride (0.010 mL, 0.043 mmol) was dissolved in dichloromethane (1 mL). Compound **(*S*)-9** (5.0 mg, 0.020 mmol) and *N,N*-DMAP (6.0 mg, 0.049 mmol) were added. The resulting colorless solution was stirred at ambient temperature for 1 h. Saturated aqueous sodium bicarbonate (1 mL) was added, and the mixture was diluted with ethyl acetate (3 mL) and water (3 mL). The organic layer was separated, and the aqueous phase was extracted three times with ethyl acetate (5 mL). The combined organic extracts were washed with brine, dried over anhydrous magnesium sulfate, and filtered. The filtrate was concentrated under reduced pressure, and the residue was purified *via* flash column chromatography eluting with ethyl acetate/hexane (5% ethyl acetate by volume) to give the title compound as a colorless oil (7.3 mg, 79% yield).

$[\alpha]_D^{20}$= 7.9 (c = 0.73, $CHCl_3$). ^{1}H NMR (600 MHz, $CDCl_3$): δ 7.57 – 7.51 (m, 2H), 7.44 – 7.36 (m, 3H), 5.49 (tq, *J* = 6.6, 2.2 Hz, 1H), 3.60 (t, *J* = 6.0 Hz, 2H), 3.56 (q, *J* = 1.1 Hz, 3H), 1.89 – 1.77 (m, 2H), 1.82 (d, *J* = 2.2 Hz, 3H), 1.55 – 1.43 (m, 4H), 0.88 (s, 9H), 0.04 (s, 6H). ^{13}C NMR (151 MHz, $CDCl_3$): δ 165.9, 132.2, 129.7, 128.4, 127.7, 123.4 (q, *J* = 290 Hz), 84.8 (q, *J* = 28 Hz) 83.1, 75.5, 67.2, 62.9, 55.7, 34.7, 32.3, 26.1, 21.7, 18.5, 3.7, –5.2. IR (film): $\tilde{\nu}$ 2953, 2929, 2857, 1751, 1463, 1253, 1187, 1169, 1105, 1017, 836, 776 cm^{-1}. HRMS (ESI$^+$) calcd. for $C_{24}H_{35}O_4SiF_3Na$ $[M+Na]^+$: 495.21489; found: 495.21509

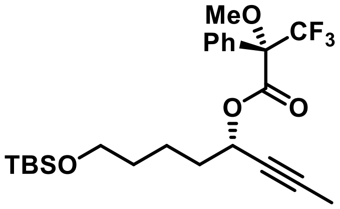

Compound S3 ((*S*)-Mosher's ester): (*R*)-Mosher's acid chloride (0.010 mL, 0.043 mmol) was dissolved in dichloromethane (1 mL). Compound **(*S*)-9** (5.0 mg, 0.020 mmol) and *N,N*-DMAP (6.0 mg, 0.049 mmol) were added. The resulting colorless solution was stirred at ambient temperature for 1 h. Saturated aqueous sodium bicarbonate (1 mL) was added, and the reaction was diluted with ethyl acetate (3 mL) and water (3 mL). The organic layer was separated, and the aqueous phase was extracted three times with ethyl acetate (5 mL). The combined organic extracts were washed with brine, dried over anhydrous magnesium sulfate, and filtered. The filtrate was concentrated under reduced pressure, and the residue was purified *via* flash column chromatography eluting with ethyl acetate/hexane (5% ethyl acetate by volume) to give the title compound as a colorless oil (8.3 mg, 90% yield). $[\alpha]_D^{20}$= –39.3 (c = 0.83, $CHCl_3$). ^{1}H NMR (600 MHz, $CDCl_3$): δ 7.59 – 7.51 (m, 2H), 7.45 – 7.34 (m, 3H), 5.52 (tq, *J* = 6.5, 2.2 Hz, 1H), 3.59 (q, *J* = 1.1 Hz, 3H), 3.55 (t, *J* = 6.3, Hz, 2H), 1.85 (d, *J* = 2.2 Hz, 3H), 1.81 – 1.72 (m, 2H), 1.50 – 1.45 (m, 2H), 1.44 – 1.34 (m, 2H), 0.88 (s, 9H), 0.03 (s, 6H). ^{13}C NMR (151 MHz, $CDCl_3$): δ 166.0, 132.6, 129.7, 128.5, 127.5, 123.4 (q, *J* = 290 Hz), 84.5 (q, *J* = 28 Hz), 83.2, 75.7, 66.8, 62.9, 55.6, 34.7, 32.2, 26.1, 21.4, 18.5, 3.7, –5.2. IR (film): $\tilde{\nu}$ 2952, 2929, 2857, 1751, 1453, 1251, 1185, 1169, 1105, 1018, 836, 776 cm^{-1}. HRMS (ESI$^+$) calcd. for $C_{24}H_{35}O_4SiF_3Na$ $[M+Na]^+$: 495.21489; found: 495.21497.

Mosher's ester analysis of compound (*S*)-9.[5]

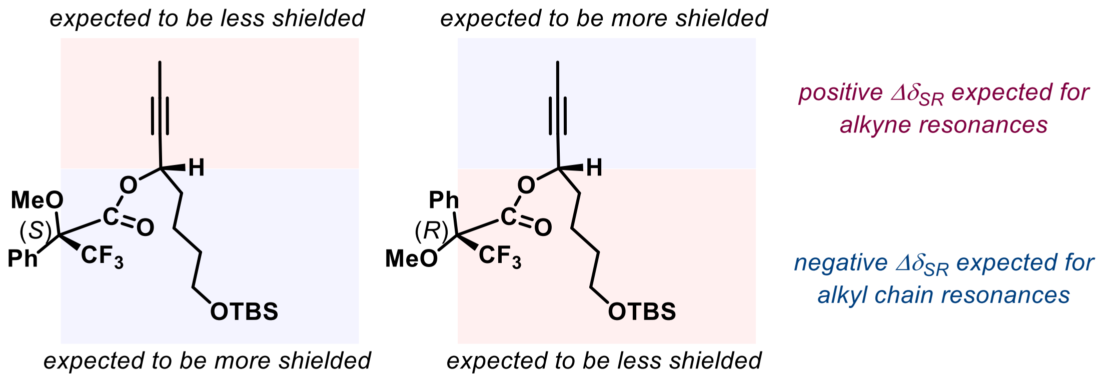

Observed$\Delta\delta_{SR}$ values (Hz):

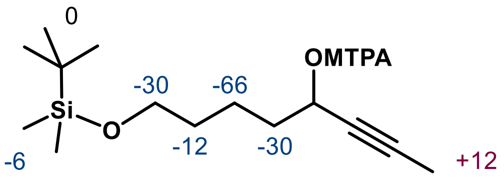

Compound assigned as (*S*)

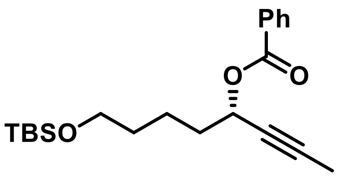

Compound (*S*)-S4: Compound **(*S*)-9** (15 mg, 0.059 mmol) was dissolved in dichloromethane (0.5 mL). Benzoic anhydride (24 mg, 0.11 mmol) was added, followed by DMAP (12 mg, 0.099 mmol), and the resulting solution was stirred at ambient temperature overnight. Saturated aqueous sodium bicarbonate (1 mL) was added, and the reaction was diluted with ethyl acetate (2 mL). The organic layer was separated, and the aqueous layer was extracted three times with ethyl acetate (5 mL). The combined organic extracts were washed with brine, dried over anhydrous magnesium sulfate, and filtered. The filtrate was concentrated under reduced pressure, and the residue was purified *via* flash column chromatography eluting with ethyl acetate/hexane (0→5% ethyl acetate by volume) to give the title compound as a colorless oil (19.5 mg, 92%). $[\alpha]_D^{20}$= –16.2 (c = 1.49, $CHCl_3$). ^1H NMR (400 MHz, $CDCl_3$): δ 8.09 – 7.99 (m, 2H), 7.55 (ddt, *J* = 7.9, 6.9, 1.4 Hz, 1H), 7.48 – 7.40 (m, 2H), 5.57 (tq, *J* = 6.5, 2.1 Hz, 1H), 3.67 – 3.59 (m, 2H), 1.94 – 1.86 (m, 2H), 1.85 (d, *J* = 2.1 Hz, 3H), 1.61 – 1.56 (m, 4H), 0.88 (s, 9H), 0.04 (s, 3H), 0.04 (s, 3H). ^{13}C NMR (101 MHz, $CDCl_3$): δ 165.8, 133.1, 130.3, 129.9, 128.5, 82.0, 77.0, 65.2, 63.0, 35.1, 32.5, 26.1, 21.7, 18.5, 3.8, –5.2.

IR (film): $\tilde{\nu}$ 2952, 2928, 2857, 1721, 1462, 1453, 1265, 1095, 835, 775 cm^{-1}. HRMS (ESI$^+$) calcd. for $C_{21}H_{32}O_3SiNa$ [M+Na]$^+$: 383.20129; found: 383.20125.

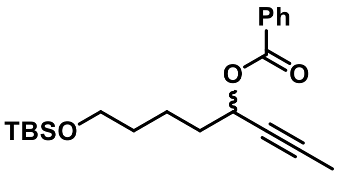

Compound (±)-S4. Prepared analogously starting with compound **(±)-9**. The title compound was isolated as a colorless oil (20 mg, 93% yield). NMR, MS, IR, and R_f data matched those obtained for compound **(*S*)-S4**.

Chiral stationary phase HPLC data of (±)-S4 and (*S*)-S4:

SUJ-SA-186-01 Racemat
150 mm Chiralcel OJ-3R,3 µm, 4.6 mm i.D.
CH3OH / H2O = 90:10
0.5 ml/min, 10.8 MPa, 298 K

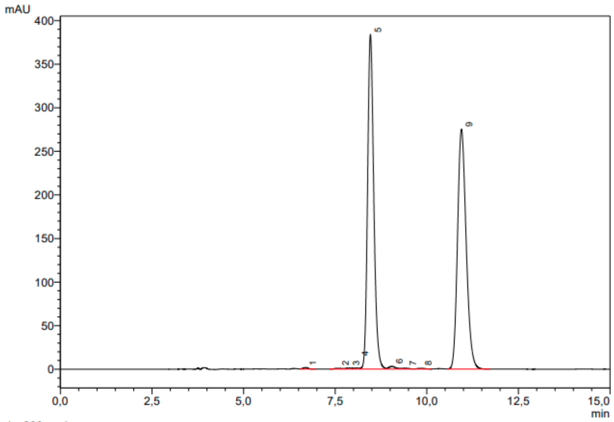

1 230nm,4nm

PDA Ch1 230nm

Peak #	Ret. Time	Area %	Name
1	6,69	0,19	
2	7,58	0,15	
3	7,87	0,18	
4	8,11	0,15	
5	8,46	49,37	1. Enantiomer
6	9,05	0,46	
7	9,42	0,17	
8	9,84	0,15	
9	10,95	49,17	2. Enantiomer
Total		100,00	

SUJ-SA-185-01
150 mm Chiralcel OJ-3R,3 µm, 4.6 mm i.D.
CH3OH / H2O = 90:10
0.5 ml/min, 10.8 MPa, 298 K

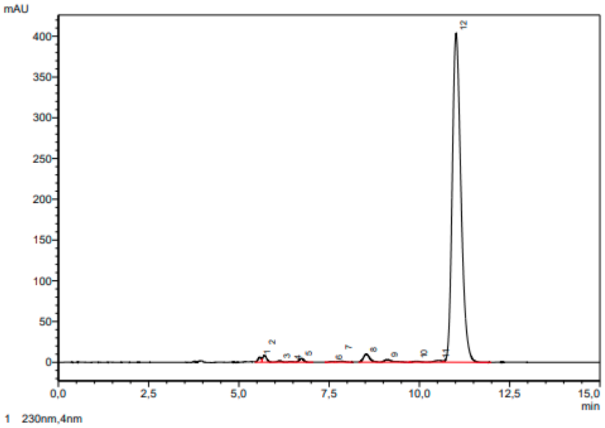

1 230nm,4nm

PDA Ch1 230nm

Peak #	Ret. Time	Area %	Name
1	5,58	0,57	
2	5,71	0,90	
3	6,13	0,25	
4	6,43	0,13	
5	6,73	0,59	
6	7,57	0,12	
7	7,84	0,28	
8	8,53	1,74	1. Enantiomer
9	9,11	0,77	
10	9,92	0,26	
11	10,54	0,48	
12	11,02	93,92	2. Enantiomer 96.4 % ee
Total		100,00	

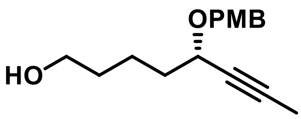

Compound (*S*)-10: A solution of compound **(*S*)-9** (1.54 g, 6.00 mmol) in THF (60 mL) was cooled to –78 °C before triethylamine (2.20 mL, 15.8 mmol) was added, followed by PMBBr (3.03 g, 15.1 mmol). A solution of KHMDS (1.80 g, 9.00 mmol) in THF (1.0 M in KHMDS, 9.0 mL) was then added dropwise, and the mixture was allowed to warm to ambient temperature over 1 h. After this period had elapsed, saturated aqueous ammonium chloride (20 mL) was added, and the mixture was stirred vigorously for 1 h before being diluted with water (50 mL) and ethyl acetate (100 mL). The organic layer was separated, and the aqueous phase was extracted three times with ethyl acetate (100 mL). The combined organic extracts were washed with brine, dried over anhydrous magnesium sulfate, and filtered. The filtrate was concentrated under reduced pressure, and the residue was redissolved in THF (60 mL).

The resulting solution was cooled to 0 °C before TBAF (1.0 M in THF, 7.20 mL, 7.20 mmol) was added dropwise, and the resulting colorless mixture was warmed to ambient temperature and stirred for 4 h. Saturated aqueous ammonium chloride was added (50 mL), and the mixture was diluted with water (50 mL) and ethyl acetate (100 mL). The organic layer was separated, and the aqueous layer was extracted three times with ethyl acetate (100 mL). The combined organic extracts were washed with brine, dried over anhydrous magnesium sulfate, and filtered. The filtrate was concentrated under reduced pressure, and the residue was purified *via* flash chromatography eluting with ethyl acetate/hexane (0→30% ethyl acetate by volume) to give the title compound as a colorless oil (1.16 g, 74% yield). $[\alpha]_D^{20}$= –82.3 (c = 1.01, $CHCl_3$). ^1H NMR (400 MHz, $CDCl_3$): δ 7.31 – 7.27 (m, 2H), 6.93 – 6.83 (m, 2H), 4.70 (d, *J* = 11.4 Hz, 1H), 4.41 (d, *J* = 11.4 Hz, 1H), 4.02 (tq, *J* = 6.4, 2.1 Hz, 1H), 3.80 (s, 3H), 3.63 (t, *J* = 6.2 Hz, 2H), 1.89 (d, *J* = 2.0 Hz, 3H), 1.72 (ddd, *J* = 14.8, 8.5, 6.5 Hz, 2H), 1.66 – 1.39 (m, 4H). ^{13}C NMR (101 MHz, $CDCl_3$): δ 159.3, 130.4, 129.8, 113.9, 82.0, 78.4, 70.1, 68.6, 63.0, 55.4, 35.8, 32.6, 21.7, 3.8. IR (film): $\tilde{\nu}$ 3394, 2937, 2862, 1612, 1513, 1442, 1338, 1302, 1248, 1174, 1065, 1035, 822 cm^{-1}. HRMS (ESI^+) calcd. for $C_{16}H_{22}O_3Na$ $[M+Na]^+$: 285.14612; found: 285.14616

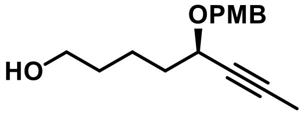

Compound (*R*)-10. Prepared analogously from **(*R*)-9**. The title compound was isolated as a colorless oil (1.18 g, 37% yield). $[\alpha]_D^{20}$= 94.0 (c = 0.80, $CHCl_3$). NMR, MS, and IR data matched those obtained for compound **(*S*)-10**.

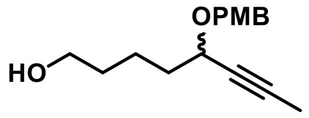

Compound (±)-10. Prepared analogously starting with compound **(±)-9**. The title compound was isolated as a colorless oil (5.1 mg, 54% yield). NMR, MS, and IR data matched those obtained for compound **(*S*)-10**.

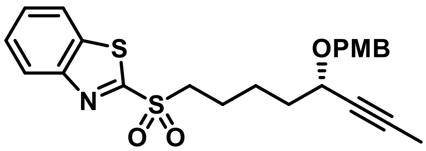

Compound (*S*)-11. Compound **(*S*)-10** (263 mg, 1.00 mmol) was dissolved in THF (5 mL) and triphenylphosphine (524 mg, 2.00 mmol), and 2-mercaptobenzothiazole (335 mg, 2.00 mmol) were added. DEAD (40% by weight in toluene, 871 mg, 0.90 mL, 1.98 mmol) was added dropwise, and the resulting yellow solution was stirred at ambient temperature for 2 h. Saturated aqueous sodium bicarbonate (5 mL) was then added, and the mixture was diluted with ethyl acetate (10 mL) and water (5 mL). The organic layer was separated, and the aqueous phase was extracted three times with ethyl acetate (20 mL). The combined organic extracts were washed with brine, dried over anhydrous magnesium sulfate, and filtered. The filtrate was concentrated under reduced pressure, and the residue was triturated with *tert*-butyl methyl ether/hexane (1:1) and the mixture filtered through a plug of silica. The filtrate was concentrated under reduced pressure. The residue was dissolved in THF (10 mL) to give a solution which was then cooled to 0 °C and stirred.

Ammonium molybdate tetrahydrate (618 mg, 0.500 mmol) was dissolved in water (4 mL) and ethanol (4 mL). Hydrogen peroxide (35% by weight in water, 4.0 mL) was added dropwise to give a bright yellow solution, which was then added portionwise (3 x 4 mL over 3 min) to the solution of the crude product in THF at 0°C. The resulting turbid yellow mixture was allowed to warm to ambient temperature and stirred for 14 h. After this time had elapsed, water (30 mL) was added, and the mixture was diluted with *tert*-butyl methyl ether (30 mL). The organic layer was separated, and the aqueous phase was extracted three times with *tert*-butyl methyl ether (50 mL). The combined organic extracts were washed with saturated aqueous sodium thiosulfate and brine, dried over anhydrous magnesium sulfate, and filtered. The filtrate was concentrated under reduced pressure, and the residue was purified *via* flash chromatography eluting with *tert*-butyl methyl ether/hexane (10→40% *tert*-butyl methyl ether by volume) to give the title compound as a pale yellow oil (393 mg, 88%). $[\alpha]_D^{20}$= –59.7 (c = 1.00, $CHCl_3$). ^1H NMR (400 MHz, $CDCl_3$): δ 8.27 – 8.18 (m, 1H), 8.05 – 7.98 (m, 1H), 7.69 – 7.55 (m, 2H), 7.26 – 7.22 (m, 2H), 6.90 – 6.81 (m, 2H), 4.66 (d, *J* = 11.4 Hz, 1H), 4.36 (d, *J* = 11.4 Hz, 1H), 3.98 (tq, *J* = 6.4, 2.1 Hz, 1H), 3.80 (s, 3H), 3.63 – 3.43 (m, 2H), 1.94 – 1.86 (m, 2H), 1.86 (d, *J* = 2.1 Hz, 3H),

1.75 – 1.65 (m, 2H), 1.65 – 1.56 (m, 2H). ^{13}C NMR (101 MHz, $CDCl_3$): δ 166.0, 159.4, 152.9, 136.9, 130.2, 129.7, 128.2, 127.8, 125.6, 122.5, 113.9, 82.4, 77.9, 70.1, 68.0, 55.4, 54.8, 35.4, 24.2, 22.3, 3.7. IR (film): $\tilde{\nu}$ 1612, 1513, 1471, 1329, 1248, 1147, 1032, 763, 520 cm^{-1}. HRMS (ESI^+) calcd. for $C_{23}H_{25}O_4S_2Na$ $[M+Na]^+$: 466.11172; found: 466.11185.

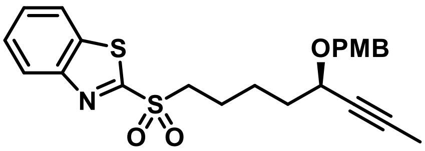

Compound (*R*)-11. Prepared analogously from compound **(*R*)-10**. The title compound was isolated as a colorless oil (374 mg, 84% yield). $[\alpha]_D^{20}$= 66.3 (c = 2.58, $CHCl_3$). NMR, MS, and IR data matched those obtained for compound **(*S*)-10**.

Control Experiment

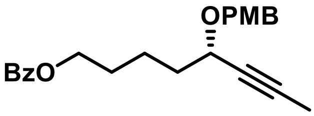

Compound (*S*)-27. Compound **(*S*)-10** (15 mg, 0.057 mmol) was dissolved in dichloromethane (0.5 mL). Benzoic anhydride (23 mg, 0.10 mmol) was added, followed by DMAP (12 mg, 0.099 mmol), and the resulting solution was stirred at ambient temperature for 3 h. Saturated aqueous sodium bicarbonate (1 mL) was added, and the mixture was diluted with ethyl acetate (2 mL). The organic layer was separated, and the aqueous phase was extracted three times with ethyl acetate (5 mL). The combined organic extracts were washed with brine, dried over anhydrous magnesium sulfate, and filtered. The filtrate was concentrated under reduced pressure, and the residue was purified *via* flash column chromatography eluting with ethyl acetate/hexane (10→15% ethyl acetate by volume) to give the title compound as a colorless oil (16.1 mg, 77%). $[\alpha]_D^{20}$= –49.5 (c = 0.41, $CHCl_3$). ^1H NMR (400 MHz, $CDCl_3$): δ 8.10 – 8.00 (m, 2H), 7.60 – 7.50 (m, 1H), 7.48 – 7.37 (m, 2H), 7.31 – 7.26 (m, 2H), 6.92 – 6.80 (m, 2H), 4.71 (d, *J* = 11.4 Hz, 1H), 4.42 (d, *J* = 11.4 Hz, 1H), 4.31 (t, *J* = 6.5 Hz, 2H), 4.04 (tq, *J* = 6.4, 2.1 Hz, 1H), 3.79 (s, 3H), 1.87 (d, *J* = 2.1 Hz, 3H), 1.83 – 1.71 (m, 4H), 1.62 (m, 2H). ^{13}C NMR (101 MHz, $CDCl_3$): δ 166.8, 159.3, 132.9, 130.6, 130.4, 129.72, 129.70, 128.5, 113.9, 82.1, 78.3, 70.1, 68.4, 65.0, 55.4, 35.7, 28.6, 22.1, 3.7. IR (film): $\tilde{\nu}$ 1716, 1513, 1452, 1271, 1246, 1174, 1110, 1070, 1028, 820, 711 cm^{-1}. HRMS (ESI^+) calcd. for $C_{23}H_{26}O_4Na$ $[M+Na]^+$: 389.17233; found: 389.17232.

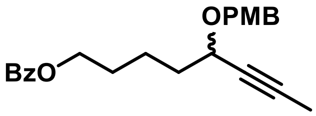

Compound (±)-27. Prepared analogously starting with compound **(±)-10**. The title compound was isolated as a colorless oil (4.5 mg, 71% yield). NMR, MS, and IR data matched those obtained for compound **(*S*)-27**.

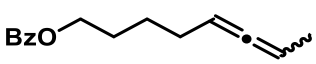

Compound 28. According to the modified procedure of Gagosz *et al.,*[6] compound **(*S*)-27** (5.0 mg, 0.014 mmol) was dissolved in dichloromethane (0.1 mL). Complex **26** (0.3 mg, 0.001 mmol) was added as a solution in dichloromethane (0.1 mL), and the resulting solution was stirred at 30 °C bath temperature for 1 h. The mixture was then filtered through a plug of silica, eluting with ethyl acetate. The filtrate was concentrated under reduced pressure, and the residue was purified *via* flash chromatography eluting with ethyl acetate/hexane (0→10% ethyl acetate by volume) to give the title compound as a colorless oil (2.0 mg, 63%). ^{1}H NMR (600 MHz, $CDCl_3$): δ 8.08 – 8.02 (m, 2H), 7.58 – 7.51 (m, 1H), 7.47 – 7.40 (m, 2H), 5.12 – 5.00 (m, 2H), 4.33 (t, *J* = 6.6 Hz, 2H), 2.11 – 1.99 (m, 2H), 1.86 – 1.77 (m, 2H), 1.66 – 1.63 (m, 3H), 1.60 – 1.55 (m, 2H). ^{13}C NMR (151 MHz, $CDCl_3$): δ 205.0, 166.8, 133.0, 130.7, 129.7, 128.5, 89.9, 86.0, 65.1, 28.5, 28.2, 25.7, 14.7. IR (film): $\tilde{\nu}$ 2931, 1718, 1452, 1315, 1273, 1776, 1115, 1070, 1027, 711 cm^{-1}. HRMS (EI) calcd. for $C_{15}H_{18}O_2$ $[M]^+$: 230.13013; found: 230.13022.

This compound did not exhibit any optical rotation. A sample prepared from compound **(±)-27** using the above procedure was identical in all respects.

Chiral stationary phase HPLC data:

Sample prepared from compound **(*S*)-27:**

SUJ-SA-239-01
150 mm Chiralpak IA, 3 µm, 4.6 mm i. D.,
MeOH / H2O = 85:15
1.0 ml/min / 149 bar / 298 K

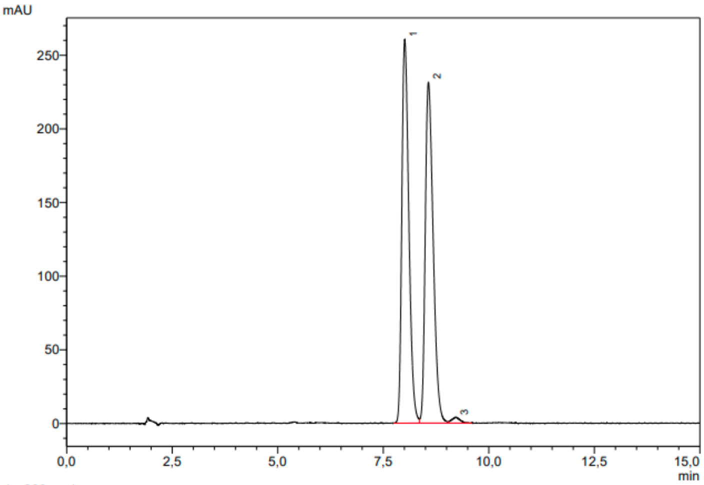

1 230nm,4nm

PDA Ch1 230nm

Peak #	Ret. Time	Area %	Name
1	8,01	49,45	1. Enantiomer
2	8,57	49,68	2. Enantiomer racemic
3	9,22	0,87	
Total		100,00	

Sample prepared from compound **(±)-27:**

SUJ-SA-264-01 Racemat
150 mm Chiralpak IA, 3 µm, 4.6 mm i. D.,
MeOH / H2O = 85:15
1.0 ml/min / 149 bar / 298 K

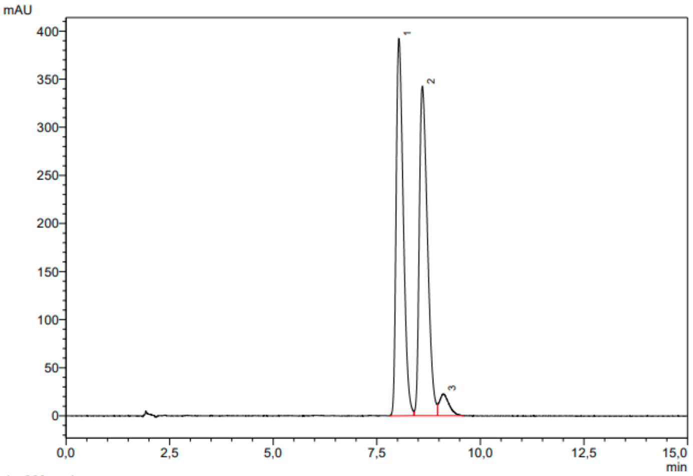

1 230nm,4nm

PDA Ch1 230nm

Peak #	Ret. Time	Area %	Name
1	8,04	48,06	1. Enantiomer
2	8,60	48,24	2. Enantiomer
3	9,11	3,70	
Total		100,00	

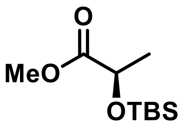

Compound S5.[9] Methyl D-lactate (2.07 g, 1.90 mL, 19.9 mmol) was dissolved in dichloromethane (50 mL) and the resulting solution was cooled to 0 °C. Imidazole (2.70 g, 39.7 mmol) was added, followed by TBSCl (4.50 g, 29.9 mmol). A white suspension rapidly developed, and the mixture was warmed to ambient temperature and stirred for 3 h. Saturated aqueous sodium bicarbonate (50 mL) was then added and the mixture was diluted with dichloromethane (50 mL) and water (50 mL). The organic layer was separated, and the aqueous phase was extracted three times with dichloromethane (100 mL). The combined organic extracts were washed with brine, dried over anhydrous magnesium sulfate, and filtered. The filtrate was concentrated under reduced pressure, and the residue was taken forward without further purification due to its acceptable purity and slight volatility.

For analytical purposes, an aliquot was purified *via* flash chromatography eluting with *tert*-butyl methyl ether/hexane (0→10% *tert*-butyl methyl ether by volume) to give the title compound as a colorless oil. $[\alpha]_D^{20}$= 33.4 (c = 0.50, $CHCl_3$) (lit.:[9] $[\alpha]_D^{24}$= 28.8, c = 2.35, $CHCl_3$). ^{1}H NMR (400 MHz, $CDCl_3$): δ 4.33 (q, *J* = 6.8 Hz, 1H), 3.72 (s, 3H), 1.40 (d, *J* = 6.8 Hz, 3H), 0.90 (s, 9H), 0.10 (s, 3H), 0.07 (s, 3H). ^{13}C NMR (101 MHz, $CDCl_3$): δ 174.5, 68.3, 51.8, 25.7, 21.3, 18.3, –5.0, –5.3. IR (film): $\tilde{\nu}$ 2954, 2931, 2858, 1759, 1463, 1255, 1142, 1061, 1004, 976, 829, 777 cm^{-1}. HRMS (ESI$^+$) calcd. for $C_{10}H_{22}O_3SiNa$ [M+Na]$^+$: 241.12304; found: 241.12306.

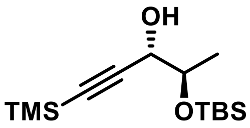

Compound 14.[10] Compound **13** (crude, assumed 20.0 mmol) was dissolved in dichloromethane (200 mL) and the resulting solution was cooled to –78 °C. Dibal-H (1.0 M in dichloromethane, 40.0 mL, 40.0 mmol) was added dropwise in 10 mL portions over 1 h and the resulting solution was stirred at –78 °C for 1 h. Methanol (10 mL) was then added dropwise and stirring was continued at –78 °C for 10 min, after which time the reaction was warmed to ambient temperature. Saturated aqueous ammonium chloride (50 mL) and saturated aqueous sodium potassium tartrate (50 mL) were added, and the mixture was diluted with water (20 mL) and dichloromethane (50 mL). The reaction mixture was stirred rapidly for 1 h at ambient temperature. After this period had elapsed, the organic layer was separated, and the aqueous phase was extracted three times with dichloromethane (200 mL). The combined organic extracts were washed with brine, dried over anhydrous magnesium sulfate, and filtered. The

filtrate was concentrated under reduced pressure, and the residue was dissolved in THF (20 mL).

In a separate Schlenk flask, trimethylsilylacetylene (4.69 g, 6.75 mL, 47.9 mmol) was dissolved in THF (150 mL) and the resulting solution was cooled to –78 °C. *n*-Butyllithium (1.6 M in hexanes, 24.0 mL, 38.4 mmol) was added dropwise, and the mixture was warmed to ambient temperature over 30 min. The mixture was cooled back to –78 °C and the THF solution of the product of the previous step was added dropwise over 20 min. The reaction was stirred for 2 h at –78 °C before it was warmed to ambient temperature. Saturated aqueous ammonium chloride (50 mL) was added, and the mixture was diluted with ethyl acetate (100 mL) and water (100 mL). The organic layer was separated, and the aqueous phase was extracted three times with ethyl acetate (200 mL). The combined organic extracts were washed with brine, dried over anhydrous magnesium sulfate, and filtered. The filtrate was concentrated under reduced pressure, and the residue was purified *via* flash column chromatography eluting with *tert*-butyl methyl ether /hexane (0→5% *tert*-butyl methyl ether by volume) to give the title compound as a white solid (4.84 g, 85% yield, *E:Z* ≈8:1). ^{1}H NMR (400 MHz, $CDCl_3$): δ 4.23 (br d, *J* = 3.8 Hz, 1H), 3.91 (qd, *J* = 6.2, 3.8 Hz, 1H), 2.35 (br s, 1H), 1.22 (d, *J* = 6.2 Hz, 3H), 0.90 (s, 9H), 0.17 (s, 9H), 0.09 (s, 3H), 0.09 (s, 3H). ^{13}C NMR (101 MHz, $CDCl_3$) δ 103.8, 90.9, 71.1, 67.6, 25.9, 18.3, 18.2, 0.0, –4.3, –4.6. IR (film): $\tilde{\nu}$ 3200 (br), 2955, 2929, 2896, 2857, 1292, 1249, 1132, 1109, 1056, 1009, 968, 876, 831, 777, 759, 698, 671 cm^{-1} HRMS (ESI$^+$) calcd. for $C_{14}H_{30}O_2Si_2Na$ [M+Na]$^+$: 309.16766; found 309.16761.

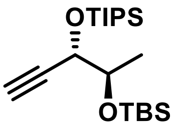

Compound 15. Compound **14** (2.50 g, 8.72 mmol, approximately 8:1 mixture of diastereomers) was dissolved in dichloromethane (87 mL) and the solution was cooled to 0 °C. 2,6-Lutidine (1.84 g, 2.00 mL, 17.2 mmol) was added, followed by dropwise addition of triisopropylsilyl triflate (3.42 g, 3.00 mL, 11.2 mmol) over 3 min. The resulting solution was warmed to ambient temperature and stirred for 1 h. Saturated aqueous sodium bicarbonate (50 mL) was added, and the mixure was diluted with dichloromethane (50 mL) and water (50 mL). The organic layer was separated, and the aqueous phase was extracted three times with dichloromethane (100 mL). The combined organic extracts were washed with brine, dried over anhydrous magnesium sulfate, and filtered. The filtrate was concentrated under reduced pressure, and the residue was dissolved in methanol (80 mL).

Potassium carbonate (5.50 g, 39.8 mmol) was added to this solution, and the resulting suspension was stirred at ambient temperature for 1 h. Saturated aqueous ammonium chloride (200 mL) was added, and the mixture was diluted with water (100 mL) and ethyl acetate (200 mL). The organic layer was separated, and the aqueous phase was extracted three times with ethyl acetate (200 mL). The combined organic extracts were washed with brine, dried over anhydrous magnesium sulfate, and filtered. The filtrate was concentrated under reduced pressure, and the residue was purified *via* flash chromatography eluting with hexane to give the title compound as a colorless oil (2.47 g, 85% yield, single diastereomer). $[\alpha]_D^{20}$= 20.5 (c = 0.40, $CHCl_3$). ^{1}H NMR (400 MHz, $CDCl_3$): δ 4.27 (dd, *J* = 5.0, 2.1 Hz, 1H), 3.83 (qd, *J* = 6.1, 5.0 Hz, 1H), 2.34 (d, *J* = 2.1 Hz, 1H), 1.23 (d, *J* = 6.1 Hz, 3H), 1.13 – 1.04 (m, 21H), 0.89 (s, 9H), 0.08 (s, 3H), 0.07 (s, 3H). ^{13}C NMR (101 MHz, $CDCl_3$): δ 84.7, 73.1, 72.8, 68.6, 26.0, 19.4, 18.24, 18.23, 12.5, –4.38, –4.42. IR (film) $\tilde{\nu}$ 3312, 2930, 2892, 2866, 1463, 1376, 1313, 1253, 1157, 1106, 1067, 1043, 991, 950, 882, 833, 804, 775, 680, 656 cm^{-1}. HRMS (ESI$^+$) calcd. for $C_{21}H_{42}O_2Si_2Na$ [M+Na]$^+$: 393.26156; found 393.26124.

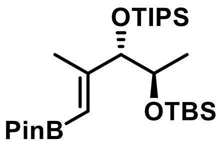

Compound 16. According to the modified procedure of Tortosa *et al.,*[11] copper(I) chloride (15 mg, 0.16 mmol), Xantphos (92 mg, 0.16 mmol), and $(BPin)_2$ (812 mg, 3.20 mmol) were dissolved in THF (10 mL), and the resulting solution was stirred at ambient temperature. Sodium *tert*-butoxide (298 mg, 3.10 mmol) was added, and the resulting brown solution was stirred for 30 min. Compound **15** (500 mg, 1.35 mmol) was added as a solution in THF (5 mL) and the resulting mixture was stirred for a further 5 min before being warmed to 40 °C. A solution of iodomethane (770 mg, 0.33 mL, 5.43 mmol) in THF (5 mL) was then added dropwise over 2 h. After this addition was complete, stirring was continued for 1 h. Water (5 mL) was then added, and the mixture was stirred vigorously for 15 min before being diluted with *tert*-butyl methyl ether (20 mL). The organic layer was separated, and the aqueous phase was extracted three times with *tert*-butyl methyl ether (20 mL). The combined organic extracts were washed with brine, dried over anhydrous magnesium sulfate, and filtered. The filtrate was concentrated under reduced pressure, and the residue was purified *via* flash chromatography eluting with *tert*-butyl methyl ether/hexane (0→5% *tert*-butyl methyl ether by volume) to give the title compound as a colorless oil (555 mg, 81% yield). $[\alpha]_D^{20}$= 11.9 (c = 0.97, $CHCl_3$). ^{1}H NMR (400 MHz, $CDCl_3$): δ 5.40 – 5.26 (m, 1H), 3.96 (dd, *J* = 5.0, 1.0 Hz, 1H), 3.74 (qd, *J* = 6.1, 4.9 Hz, 1H), 1.97 (d, *J* = 1.0 Hz, 3H), 1.32 – 1.20 (m, 12H), 1.14 (d, *J* = 6.1 Hz, 3H), 1.07 – 1.02 (m, 21H), 0.85 (s, 9H), 0.02

(s, 3H), 0.01 (s, 3H). ^{13}C NMR (101 MHz, $CDCl_3$): δ 162.2, 84.3, 82.7, 71.7, 26.1, 25.0, 24.8, 19.8, 18.4, 18.3, 17.9, 12.7, –4.2, –4.7. [NOTE: *The ^{13}C resonance corresponding to the carbon atom bound directly to boron could not be seen in the 1D spectrum, presumably due to the nearby quadrupolar nucleus. A peak in the HSQC spectrum indicates that this carbon nucleus resonates at 115.3 ppm*]. IR (film): $\tilde{\nu}$ 2930, 2893, 2866, 1644, 1464, 1388, 1370, 1321, 1257, 1146, 1105, 1065, 1041, 1014, 987, 971, 883, 870, 835, 775, cm^{-1}. HRMS (ESI$^+$) calcd. for $C_{27}H_{57}O_4Si_2BNa$ [M+Na]$^+$: 535.37806; found 535.37889.

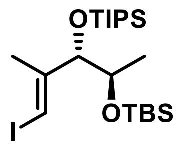

Compound S6. A solution of compound **16** (513 mg, 1.00 mmol) in THF (5 mL) was cooled to 0 °C. A solution of sodium hydroxide (600 mg, 15.0 mmol) in water (5.0 mL, 3.0 M in NaOH) was added dropwise, followed by a solution of iodine (761 mg, 3.00 mmol) in THF (5 mL). The resulting solution was stirred rapidly for 15 min before saturated aqueous sodium thiosulfate was added (10 mL), and the reaction was diluted with hexane (20 mL) and water (10 mL). The organic layer was separated, and the aqueous phase was extracted three times with hexane (20 mL). The combined organic extracts were washed with brine, dried over anhydrous magnesium sulfate, and filtered. The filtrate was concentrated under reduced pressure, and the residue was purified via flash chromatography eluting with hexane to give the title compound as a yellow oil (334 mg, 65% yield). $[\alpha]_D^{20}$= 16.4 (c = 1.01, $CHCl_3$). ^{1}H NMR (400 MHz, $CDCl_3$): δ 6.20 – 6.11 (m, 1H), 4.03 (dd, *J* = 6.0, 0.7 Hz, 1H), 3.71 (p, *J* = 6.0 Hz, 1H), 1.80 (d, *J* = 1.1 Hz, 3H), 1.16 (d, *J* = 6.0 Hz, 3H), 1.10 – 0.99 (m, 21H), 0.85 (s, 9H), 0.03 (s, 3H), 0.01 (s, 3H). ^{13}C NMR (101 MHz, $CDCl_3$): δ 148.9, 82.7, 80.1, 70.9, 26.0, 20.8, 20.5, 18.3, 18.2, 12.7, –4.2, –4.6. IR (film): $\tilde{\nu}$ 2945, 2929, 2892, 2866, 1463, 1383, 1274, 1256, 1111, 1066, 1040, 1013, 997, 985, 883, 834, 823, 809, 774, 681 cm^{-1}. HRMS (ESI$^+$) calcd. for $C_{21}H_{45}O_2ISi_2Na$ [M+Na]$^+$: 535.18950; found: 535.19008.

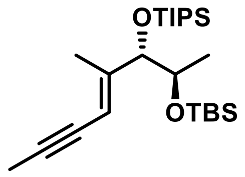

Compound 17. Copper(I) iodide (38 mg, 0.20 mmol) and [$(Ph_3P)_2PdCl_2$] (70 mg, 0.10 mmol) were dissolved in THF (2.5 mL) and diethylamine (5 mL) and the resulting solution was cooled to –20 °C. Compound **S6** (334 mg, 0.653 mmol) was added as a solution in THF (2.5 mL) before liquid propyne (1 mL transferred by cannula from a Schlenk flask at –78 °C, excess) was introduced. The mixture was stirred while slowly warming to ambient temperature overnight, during which time the mixture darkened to an opaque brown. The reaction was diluted with water (30 mL) and hexane (20 mL), the organic layer was separated, and the aqueous phase was

extracted three times with hexane (30 mL). The combined organic extracts were washed with brine, dried over anhydrous magnesium sulfate, and filtered. The filtrate was concentrated under reduced pressure, and the residue was purified *via* flash chromatography eluting with *tert*-butyl methyl ether/hexane (0→1% *tert*-butyl methyl ether by volume) to give the title compound as a pale yellow oil (251 mg, 91% yield). $[\alpha]_D^{20}$= 20.0 (c = 0.22, $CHCl_3$). ^1H NMR (400 MHz, $CDCl_3$): δ 5.64 – 5.33 (m, 1H), 3.98 (d, *J* = 4.5 Hz, 1H), 3.75 (qd, *J* = 6.2, 4.5 Hz, 1H), 1.99 (d, *J* = 2.3 Hz, 3H), 1.84 (br s, 3H), 1.11 (d, *J* = 6.2 Hz, 3H), 1.08 – 1.01 (m, 21H), 0.86 (s, 9H), 0.03 (s, 3H), 0.03 (s, 3H). ^{13}C NMR (101 MHz, $CDCl_3$): δ 149.9, 107.8, 89.7, 82.0, 71.5, 26.0, 19.4, 18.32, 18.29, 18.26, 16.3, 12.7, 4.7, –4.3, –4.7. IR (film): $\tilde{\nu}$ 2945, 2893, 2867, 1462, 1385, 1255, 1145, 1111, 1092, 1064, 883, 835, 775, 680 cm^{-1}. HRMS (ESI^+) calcd. for $C_{24}H_{48}O_2Si_2Na$ $[M+Na]^+$: 447.30850; found: 447.30845.

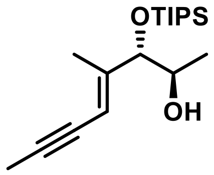

Compound 18. *para*-Toluenesulfonic acid (28 mg, 0.15 mmol) was added to a solution of compound **17** (250 mg, 0.589 mmol) in methanol (6 mL) and THF (3 mL). The resulting colorless mixture was stirred at ambient temperature for 24 h before saturated aqueous sodium bicarbonate (5 mL) was added. The reaction was diluted with ethyl acetate (20 mL) and water (10 mL), the organic layer was separated, and the aqueous phase was extracted three times with ethyl acetate (20 mL). The combined organic extracts were washed with brine, dried over anhydrous magnesium sulfate, and filtered. The filtrate was concentrated under reduced pressure, and the residue was purified *via* flash column chromatography eluting with *tert*-butyl methyl ether/hexane (0→10% *tert*-butyl methyl ether by volume) to give the title compound as a pale yellow oil (164 mg, 90% yield). $[\alpha]_D^{20}$= 38.6 (c = 0.51, $CHCl_3$). ^1H NMR (400 MHz, $CDCl_3$): δ 5.49 – 5.35 (m, 1H), 4.06 (d, *J* = 4.4 Hz, 1H), 3.93 – 3.78 (m, 1H), 2.21 (d, *J* = 3.6 Hz, 1H), 2.00 (d, *J* = 2.3 Hz, 3H), 1.89 (br s, 3H), 1.14 – 0.98 (m, 24H). ^{13}C NMR δ (101 MHz, $CDCl_3$): δ 148.5, 108.7, 90.5, 80.8, 76.8, 70.3, 18.19, 18.17, 17.8, 16.1, 12.6, 4.6. IR (film): $\tilde{\nu}$ 3442 (br), 2944, 2892, 2867, 1463, 1386, 1365, 1253, 1088, 1062, 1015, 997, 883, 856, 681, 658 cm^{-1}. HRMS (ESI^+) calcd. for $C_{18}H_{34}O_2SiNa$ $[M+Na]^+$: 333.22203; found: 333.22267.

Fragment Coupling and Completion of the Total Synthesis

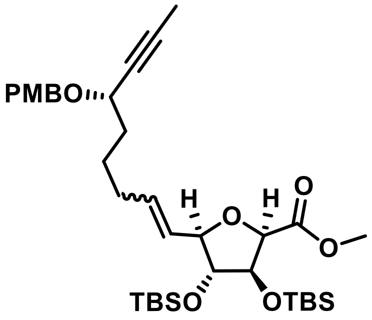

Compound 19. A solution of compound (***S***)-**11** (90.2 mg, 0.200 mmol) in THF (5 mL) was cooled to –100 °C in a three-necked round bottomed flask equipped with an internal thermometer. A solution of LDA (0.5 M in THF, 0.40 mL, 0.200 mmol) was added dropwise over 5 min, maintaining a inner temperature below –85 °C. The addition of LDA gave rise to an intensely orange, clear solution [NOTE*: during optimization, it was observed that allowing the reaction to warm to –65 °C during this phase of the process resulted in extensive decomposition, which was visible as a darkening and clouding of the solution to give a turbid, yellow-brown mixture*]. The solution was stirred for 5 min whilst the reaction was cooled back to –100 °C.

A solution of compound **6** (85.0 mg, 0.20 mmol) in DMF (5 mL) was cooled to –65 °C. This cold solution was added to the lithiated sulfone solution *via* rapid cannula transfer; upon this addition, the bright orange color paled to a light yellow [NOTE: *during optimization, it was observed that dropwise addition of the aldehyde solution to the lithiated sulfone solution resulted in decomposition. Likewise,attempts to add the lithiated sulfone solution to the aldehyde solution* via *either a cooled jacketed addition funnel or rapid cannula transfer resulted in decomposition of the exceptionally temperature-sensitive lithiated sulfone*]. The mixture was allowed to warm to ambient temperature over 2 h. After this time had elapsed, saturated aqueous ammonium chloride (20 mL) was added, and the mixture was diluted with *tert*-butyl methyl ether (20 mL) and water (20 mL). The organic layer was separated, and the aqueous phase was extracted three times with *tert*-butyl methyl ether (30 mL). The combined organic extracts were washed five times with water (10 mL) followed by washing with brine, before they were dried over anhydrous magnesium sulfate and filtered. The filtrate was concentrated under reduced pressure, and the residue was purified *via* flash chromatography eluting with *tert*-butyl methyl ether /hexane (0→20% *tert*-butyl methyl ether by volume) to give the title compound as a colorless oil (133 mg, 75% yield, *E:Z* ≈ 6:1). As the material contained ca. 10% of aldehyde **6**, an analytically pure sample was obtained by washing with $NaHSO_3$ in accordance with a literature procedure.[12] ^1H NMR (400 MHz, $CDCl_3$): δ 7.30 – 7.26 (m, 2H), 6.92 – 6.81 (m, 2H), 5.74 – 5.59 (m, 2H), 4.69 (d, *J* = 11.5 Hz, 1H), 4.67 – 4.61 (m, 1H), 4.41 (d, *J* = 11.5 Hz, 1H), 4.24 – 4.20 (m, 2H), 3.99 (tq, *J* = 6.6, 2.2 Hz, 1H), 3.89 (t, *J* = 1.8 Hz,

1H), 3.80 (s, 3H), 3.75 (s, 3H), 2.12 – 1.96 (m, 2H), 1.88 (d, *J* = 2.2 Hz, 3H), 1.75 – 1.64 (m, 2H), 1.62 – 1.41 (m, 2H), 0.88 (s, 9H), 0.86 (s, 9H), 0.08 (s, 6H), 0.06 (s, 3H), 0.05 (s, 3H). ^{13}C NMR (101 MHz, $CDCl_3$): δ 170.3, 159.3, 133.7, 130.5, 129.7, 129.3, 113.9, 89.5, 82.5, 81.9, 81.7, 80.2, 78.5, 70.0, 68.6, 55.4, 52.0, 35.9, 32.1, 25.9, 25.7, 24.9, 18.0, 17.9, 3.8, –4.2, –4.41, –4.42, –5.1. IR (film): $\tilde{\nu}$ 2952, 2930, 2858, 1736, 1514, 1251, 1205, 1087, 837, 778 cm^{-1}. HRMS (ESI$^+$) calcd. for $C_{35}H_{58}O_7Si_2Na$[M+Na]$^+$: 669.36133; found: 669.36182.

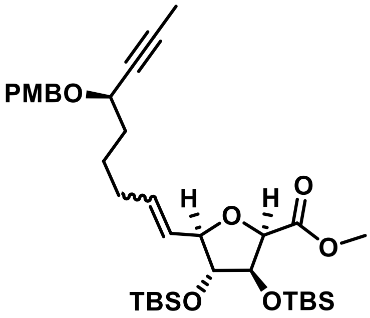

Compound 11-*epi*-19. Prepared analogously from sulfone (***R***)-**11** (106 mg, 0.240 mmol) and aldehyde **6** (100 mg, 0.238 mmol); colorless oil (133 mg, 85% yield, *E/Z* ≈ 8:1, contaminated with ca. 10% of unreacted aldehyde **6**). ^{1}H NMR (400 MHz, $CDCl_3$): δ 7.29 – 7.26 (m, 2H), 6.91 – 6.81 (m, 2H), 5.72 – 5.60 (m, 2H), 4.69 (d, *J* = 11.4 Hz, 1H), 4.66 (d, *J* = 3.9 Hz, 1H), 4.40 (d, *J* = 11.4 Hz, 1H), 4.27 – 4.18 (m, 2H), 4.00 (tq, *J* = 6.5, 2.0 Hz, 1H), 3.89 (t, *J* = 1.8 Hz, 1H), 3.80 (s, 3H), 3.74 (s, 3H), 2.09 – 1.94 (m, 2H), 1.88 (d, *J* = 2.0 Hz, 3H), 1.78 – 1.60 (m, 2H), 1.57 – 1.46 (m, 2H), 0.88 (s, 9H), 0.86 (s, 9H), 0.08 (s, 3H), 0.07 (s, 3H), 0.06 (s, 3H), 0.05 (s, 3H). ^{13}C NMR (101 MHz, $CDCl_3$): δ 170.3, 159.3, 133.8, 130.5, 129.7, 129.3, 113.9, 89.5, 82.4, 82.0, 81.7, 80.2, 78.5, 70.0, 68.6, 55.4, 51.9, 35.8, 32.1, 25.9, 25.7, 24.8, 18.0, 17.9, 3.8, –4.2, –4.41, –4.43, –5.1. IR (film): $\tilde{\nu}$ 2952, 2929, 2857, 1735, 1514, 1463, 1251, 1205, 1087, 931, 836, 777 cm^{-1}. HRMS (ESI$^+$) calcd. for $C_{35}H_{58}O_7Si_2Na$ [M+Na]$^+$: 669.36133; found: 669.36125.

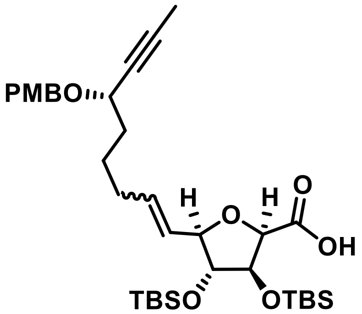

Compound S7. Compound **19** (60.3 mg, 0.093 mmol) was dissolved in THF (1 mL) and isopropanol (3 mL), and the resulting solution was cooled to 0 °C. A solution of potassium hydroxide (1.00 mmol in 3.0 mL water, 0.33 M, 56 mg) was added, and the resulting homogeneous solution was stirred at 0 °C for 24 h. The reaction was quenched by the addition of sulfate buffer (1.0 M, pH 2, 20 mL) and the mixture allowed to warm to ambient temperature, whereupon it was diluted with ethyl acetate (20 mL). The organic layer was separated, and the aqueous phase was extracted three times with ethyl acetate (30 mL). The combined organic extracts were dried over anhydrous magnesium sulfate, and filtered. The filtrate was concentrated under reduced pressure, and the residue was purified *via* flash chromatography eluting with acetic acid/ethyl acetate/hexane (0→30% [1% acetic acid in ethyl acetate] by

volume) to give the title compound as a colorless oil (47.0 mg, 80% yield, *E/Z* ≈6:1). ^{1}H NMR (400 MHz, $CDCl_3$): δ 7.30 – 7.26 (m, 2H), 6.90 – 6.84 (m, 2H), 5.73 – 5.60 (m, 2H), 4.70 (d, *J* = 11.4 Hz, 1H), 4.68 – 4.65 (m, 1H), 4.41 (d, *J* = 11.4 Hz, 1H), 4.36 – 4.31 (m, 1H), 4.22 (td, *J* = 2.7, 1.2 Hz, 1H), 4.01 (tq, *J* = 6.8, 2.0 Hz, 1H), 3.93 – 3.89 (m, 1H), 3.80 (s, 3H), 2.10 – 1.99 (m, 2H), 1.89 (d, *J* = 2.0 Hz, 3H), 1.76 – 1.66 (m, 2H), 1.58 – 1.50 (m, 2H), 0.89 – 0.87 (m, 18H), 0.09 (s, 3H), 0.08 (s, 3H), 0.08 (s, 3H), 0.08 (s, 3H). ^{13}C NMR (101 MHz, $CDCl_3$): δ 170.0, 159.3, 134.9, 130.4, 129.7, 128.5, 113.9, 91.4, 82.7, 82.5, 82.0, 79.9, 78.4, 70.1, 68.5, 55.4, 35.8, 32.1, 25.8, 25.7, 24.8, 18.01, 18.00, 3.8, –4.47, –4.53, –4.6, –5.2. IR (film) $\tilde{\nu}$ 2952, 2930, 2858, 1736, 1514, 1252, 1206, 1131, 1087, 837, 778 cm^{-1}. HRMS (ESI^{-}) calcd. for $C_{34}H_{55}O_7Si_2$ [M-H]$^{-}$: 631.34918; found: 631.34903.

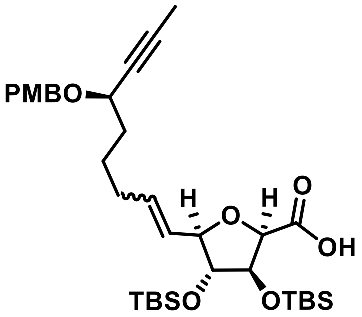

Compound 11-*epi*-S7. Prepared analogously from ester 11-*epi*-**19** (65.1 mg, 0.101 mmol) as a colorless oil (56.4 mg, 88% yield, *E/Z* ≈8:1). ^{1}H NMR (400 MHz, $CDCl_3$): δ 7.30 – 7.26 (m, 2H), 6.90 – 6.83 (m, 2H), 5.74 – 5.58 (m, 2H), 4.70 (d, *J* = 11.4 Hz, 1H), 4.67 (d, *J* = 3.2 Hz, 1H), 4.41 (d, *J* = 11.4 Hz, 1H), 4.37 – 4.29 (m, 1H), 4.24 – 4.16 (m, 1H), 4.01 (tq, *J* = 6.4, 2.0 Hz, 1H), 3.95 – 3.87 (m, 1H), 3.80 (s, 3H), 2.09 – 1.99 (m, 2H), 1.89 (d, *J* = 2.0 Hz, 3H), 1.78 – 1.62 (m, 2H), 1.60 – 1.48 (m, 2H), 0.89 (s, 9H), 0.88 (s, 9H), 0.09 (s, 3H), 0.09 (s, 3H), 0.08 (s, 3H), 0.07 (s, 3H). ^{13}C NMR (101 MHz, $CDCl_3$): δ 170.0, 159.3, 135.0, 130.4, 129.7, 128.5, 113.9, 91.4, 82.7, 82.5, 82.0, 79.9, 78.4, 70.1, 68.4, 55.4, 35.8, 32.0, 25.8, 25.7, 24.7, 18.0, 3.8, –4.47, –4.53, –4.6, –5.2. IR (film): $\tilde{\nu}$ 2951, 2928, 2857, 1731, 1612, 1513, 1463, 1250, 1132, 1084, 938, 836, 778 cm^{-1}. HRMS (ESI^{-}) calcd. for $C_{34}H_{55}O_7Si_2$ [M-H]$^{-}$: 631.34918; found: 631.34968.

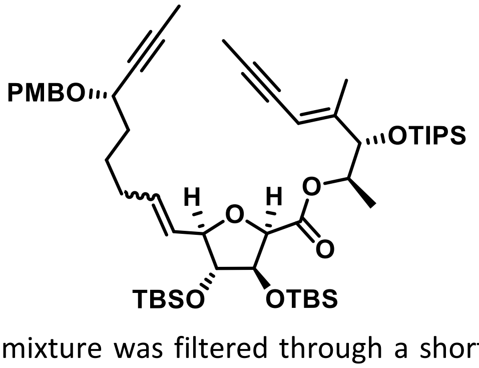

Compound 20. Compound **S7** (44.3 mg, 0.070 mmol) was dissolved in THF (0.7 mL). Trichlorobenzoyl chloride (12 µL, 0.077 mmol) was added, followed by triethylamine (14 µL, 0.100 mmol). The resulting solution was stirred for 16 h at ambient temperature, during the course of which it became turbid. The mixture was filtered through a short plug of Celite™ into a fresh flame dried Schlenk flask, eluting with dry diethyl ether (2 x 1 mL). The resulting solution was concentrated under a flow of argon, and then dried under high vacuum. The residue was dissolved in toluene (0.5 mL).

Compound **18** (21.9 mg, 0.071 mmol) and *N*,*N*-DMAP (10.0 mg, 0.082 mmol) were added as a solution in toluene (0.5 mL), resulting in the rapid formation of a white precipitate, and the resulting mixture was stirred at ambient temperature for 2 h. Water (5 mL) was then added and the reaction diluted with ethyl acetate (1 mL).The organic layer was separated, and the aqueous phase was extracted three times with ethyl acetate (5 mL). The combined organic extracts were washed with brine, dried over anhydrous magnesium sulfate, and filtered. The filtrate was concentrated under reduced pressure, and the residue was purified *via* flash chromatography eluting with *tert*-butyl methyl ether/hexane (0→10% *tert*-butyl methyl ether by volume) to give the title compound as a colorless oil (49.0 mg, 76% yield, *E*/*Z* $\approx$ 6:1). ^{1}H NMR (400 MHz, $CDCl_3$): δ 7.30 – 7.26 (m, 2H), 6.90 – 6.83 (m, 2H), 5.70 – 5.58 (m, 3H), 4.87 (qd, *J* = 6.2, 2.5 Hz, 1H), 4.69 (d, *J* = 11.4 Hz, 1H), 4.62 – 4.58 (m, 1H), 4.41 (d, *J* = 11.4 Hz, 1H), 4.38 (br s, 1H), 4.22 (dt, *J* = 5.5, 1.5 Hz, 1H), 4.20 – 4.17 (m, 1H), 3.99 (tq, *J* = 5.7, 2.0 Hz, 1H), 3.90 – 3.87 (m, 1H), 3.80 (s, 3H), 2.08 – 2.00 (m, 2H), 1.99 (d, *J* = 2.3 Hz, 3H), 1.88 (d, *J* = 2.0 Hz, 3H), 1.83 (br s, 3H), 1.75 – 1.65 (m, 2H), 1.56 – 1.49 (m, 2H), 1.20 (d, *J* = 6.2 Hz, 3H), 1.09 – 1.04 (m, 21H), 0.89 (s, 9H), 0.86 (s, 9H), 0.08 (s, 3H), 0.07 (s, 3H), 0.07 (s, 3H), 0.06 (s, 3H). ^{13}C NMR (101 MHz, $CDCl_3$): δ 168.7, 159.3, 147.8, 133.0, 130.6, 129.6, 129.5, 113.9, 108.0, 90.8, 89.4, 82.7, 81.9, 81.7, 80.2, 78.5, 77.9, 77.0, 73.7, 70.0, 68.7, 55.4, 35.9, 32.1, 25.9, 25.8, 24.9, 18.24, 18.23, 18.04, 18.00, 16.9, 12.8, 12.8, 4.7, 3.8, –4.1, –4.3, –4.5, –4.9. IR (film) $\tilde{\nu}$ 2930, 2896, 2861, 1730, 1514, 1463, 1388, 1363, 1031, 1251, 1174, 1085, 1041, 882, 837, 778, 666 cm^{-1}. HRMS (ESI$^+$) calcd. for $C_{52}H_{88}O_8Si_3Na$ [M+Na]$^+$: 947.56792; found: 947.56724.

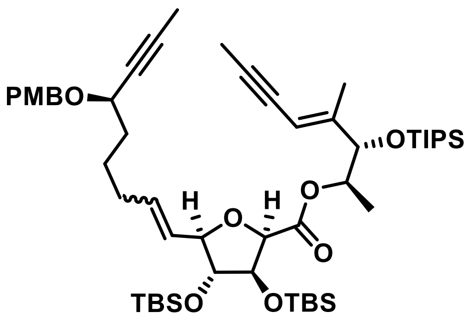

Compound 11-*epi*-20. Prepared analogously from compound **11-*epi*-S7** (40.4 mg, 0.063 mmol) and alcohol **18** (19.2 mg, 0.061 mmol) as a colorless oil (52.3 mg, 92% yield, *E*/*Z* $\approx$ 8:1). ^{1}H NMR (400 MHz, $CDCl_3$) δ 7.30 – 7.26 (m, 2H), 6.90 – 6.82 (m, 2H), 5.73 – 5.57 (m, 3H), 4.87 (qd, *J* = 6.2, 2.4 Hz, 1H), 4.69 (d, *J* = 11.4 Hz, 1H), 4.59 (d, *J* = 3.4 Hz, 1H), 4.41 (d, *J* = 11.4 Hz, 1H), 4.38 (br s, 1H), 4.24 – 4.20 (m, 1H), 4.18 (dd, *J* = 3.4, 1.4 Hz, 1H), 4.04 – 3.96 (m, 1H), 3.89 (t, *J* = 1.4 Hz, 1H), 3.80 (s, 3H), 2.07 – 2.00 (m, 2H), 1.99 (d, *J* = 2.4 Hz, 3H), 1.88 (d, *J* = 2.1 Hz, 3H), 1.83 (br s, 3H), 1.78 – 1.60 (m, 2H), 1.56 – 1.48 (m, 2H), 1.20 (d, *J* = 6.2 Hz, 3H), 1.10 – 1.03 (m, 21H), 0.89 (s, 9H), 0.86 (s, 9H), 0.08 (s, 6H), 0.07 (s, 3H), 0.06 (s, 3H). ^{13}C NMR (101 MHz, $CDCl_3$): δ 168.7, 159.3, 147.8, 133.1, 130.5, 129.7, 129.5, 113.9, 108.0, 90.8, 89.4, 82.7, 81.8, 81.7, 80.2, 78.5, 77.9, 77.0, 73.7, 70.0, 68.6,

55.4, 35.9, 32.1, 25.9, 25.8, 24.9, 18.2, 18.03, 18.00, 16.9, 12.80, 12.75, 4.7, 3.8, –4.1, –4.3, –4.5, –4.9. IR (film): $\tilde{\nu}$ 2928, 2859, 1732, 1513, 1463, 1463, 1388, 1364, 1252, 1173, 1083, 882, 837, 778, 681 cm^{-1}. HRMS (ESI$^+$) calcd. for $C_{52}H_{88}O_8Si_3Na$ [M+Na]$^+$: 947.56792; found: 947.56826.

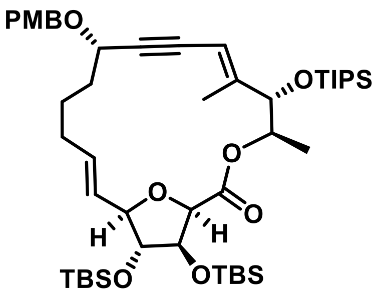

Compound 21. Compound **20** (34.0 mg, 0.037 mmol) was dissolved in toluene (12 mL), and activated molecular sieves (5 Å, 200 mg) were added. The flask was immersed into an oil bath at 90 °C (bath temperature). A pink solution of catalyst **25** (4.2 mg, 0.0037 mmol) in toluene (3 mL) was added to the mixture once the temperature was stable at 90 °C, resulting in a pale orange mixture. Stirring was continued at 90 °C for 2 h, after which time ethanol (2 mL) was added to quench the catalyst, and the suspension was allowed to cool to ambient temperature. Filtration of the mixture through a plug of Celite™ gave a colorless solution, which was concentrated under reduced pressure; the residue was purified *via* flash chromatography eluting with *tert*-butyl methyl ether/hexane (0→10% *tert*-butyl methyl ether by volume) to give the title compound as a colorless oil (27.2 mg, 84% yield), along with the isomeric *Z*-alkene derivative **S8** (see below). $[\alpha]_D^{20}$= –66.0 (c = 1.00, $CHCl_3$). ^1H NMR (400 MHz, $CDCl_3$) δ 7.33 – 7.27 (m, 2H), 6.91 – 6.83 (m, 2H), 5.74 – 5.57 (m, 2H), 5.47 – 5.36 (m, 1H), 5.05 (qd, *J* = 6.5, 3.0 Hz, 1H), 4.73 (d, *J* = 11.3 Hz, 1H), 4.66 (d, *J* = 5.6 Hz, 1H), 4.46 (d, *J* = 11.3 Hz, 1H), 4.30 (dd, *J* = 5.6, 3.6 Hz, 1H), 4.29 – 4.25 (m, 1H), 4.22 – 4.17 (m, 1H), 4.15 (d, *J* = 2.9 Hz, 1H), 3.94 (t, *J* = 3.5 Hz, 1H), 3.80 (s, 3H), 2.17 – 2.09 (m, 1H), 2.08 (d, *J* = 1.2 Hz, 3H), 2.05 – 1.97 (m, 1H), 1.93 – 1.84 (m, 1H), 1.84 – 1.70 (m, 3H), 1.25 (d, *J* = 6.5 Hz, 3H), 1.10 – 1.00 (m, 21H), 0.87 (s, 9H), 0.86 (s, 9H), 0.08 (s, 3H), 0.07 (s, 3H), 0.06 (s, 3H), 0.04 (s, 3H). ^{13}C NMR (101 MHz, $CDCl_3$) δ 170.1, 159.3, 152.8, 133.7, 130.5, 130.2, 129.8, 113.9, 106.6, 92.3, 87.7, 84.9, 81.5, 81.2, 80.1, 79.5, 76.2, 70.2, 69.6, 55.4, 34.3, 30.9, 26.0, 25.9, 25.2, 18.19, 18.15, 18.12, 18.07, 18.0, 16.9, 12.4, –4.1, –4.2, –4.3, –4.7. IR (film): $\tilde{\nu}$ 2930, 2894, 2862, 1733, 1514, 1463, 1388, 1363, 1251, 1171, 1083, 882, 833, 778, 681 cm^{-1}. HRMS (ESI$^+$) calcd. for $C_{48}H_{82}O_8Si_3Na$ [M+Na]$^+$: 893.52097; found: 893.52090.

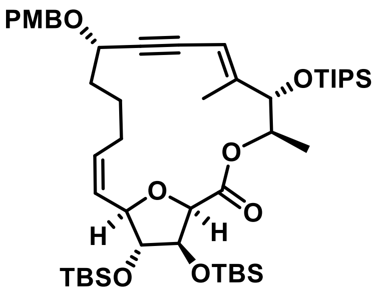

(Z)-Alkene S8. Obtained from the above reaction as minor by-product (2.0 mg, 6% yield). $[\alpha]_D^{20}$= –50.0 (c = 0.15, $CHCl_3$). ^{1}H NMR (600 MHz, $CDCl_3$): δ 7.32 – 7.28 (m, 2H), 6.92 – 6.82 (m, 2H), 5.78 (ddd, *J* = 10.0, 5.6, 4.5 Hz, 1H), 5.64 – 5.62 (m, 1H), 5.61 – 5.57 (m, 1H), 4.94 (dq, *J* = 6.5, 5.5 Hz, 1H), 4.72 (d, *J* = 11.4 Hz, 1H), 4.51 (d, *J* = 3.5 Hz, 1H), 4.45 (d, *J* = 11.4 Hz, 1H), 4.36 (ddd, *J* = 9.7, 2.6, 1.0 Hz, 1H), 4.31 (ddd, *J* = 6.5, 4.1, 1.9 Hz, 1H), 4.28 (dd, *J* = 3.5, 0.9 Hz, 1H), 4.20 (dd, *J* = 5.5, 1.0 Hz, 1H), 3.85 (dd, *J* = 2.6, 0.9 Hz, 1H), 3.80 (s, 3H), 2.51 (dtd, *J* = 13.5, 9.3, 4.5 Hz, 1H), 1.89 (d, *J* = 1.0 Hz, 3H), 1.87 – 1.76 (m, 2H), 1.77 – 1.69 (m, 1H), 1.66 – 1.58 (m, 1H), 1.35 (d, *J* = 6.5 Hz, 3H), 1.09 – 0.98 (m, 21H), 0.87 (s, 9H), 0.86 (s, 9H), 0.09 (s, 3H), 0.07 (s, 3H), 0.05 (s, 3H), 0.02 (s, 3H). ^{13}C NMR (151 MHz, $CDCl_3$) δ 169.2, 159.3, 149.7, 135.9, 130.5, 129.7, 127.7, 113.9, 108.3, 93.0, 85.1, 83.9, 82.3, 82.1, 81.8, 79.9, 74.9, 70.4, 69.7, 55.4, 35.0, 26.9, 26.7, 26.0, 25.9, 18.5, 18.2, 18.1, 18.0, 16.2, 12.5, 1.2, –4.1, –4.6, –4.7. IR (film): $\tilde{\nu}$ 2951, 2931, 2889, 2862, 1733, 1514, 1463, 1252, 1097, 1081, 882, 838, 778 cm^{-1} HRMS (ESI$^+$) calcd. for $C_{48}H_{82}O_8Si_3Na$ $[M+Na]^+$: 893.52097; found: 893.52090.

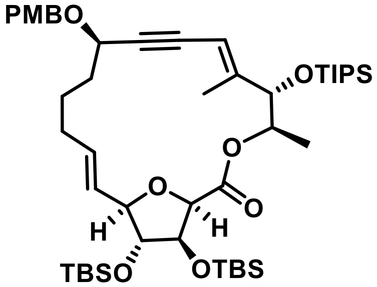

Compound 11-*epi*-21. Prepared analogously from compound **11-*epi*-20** (20.1 mg, 0.022 mmol) with the aid of catalyst **25** (2.6 mg, 0.002 mmol) at 90 °C for 2 h; colorless oil (15.0 mg, 80% yield). $[\alpha]_D^{20}$= 17.1 (c = 0.28, $CHCl_3$). ^{1}H NMR (400 MHz, $CDCl_3$): δ 7.32 – 7.27 (m, 2H), 6.91 – 6.84 (m, 2H), 5.74 – 5.57 (m, 2H), 5.42 (br s, 1H), 5.07 (qd, *J* = 6.5, 3.1 Hz, 1H), 4.74 (d, *J* = 11.4 Hz, 1H), 4.67 (d, *J* = 5.6 Hz, 1H), 4.45 (d, *J* = 11.4 Hz, 1H), 4.38 – 4.34 (m, 1H), 4.32 (dd, *J* = 5.6, 3.4 Hz, 1H), 4.20 (dt, *J* = 5.6, 3.4 Hz, 1H), 4.14 (d, *J* = 3.1 Hz, 1H), 3.96 (t, *J* = 3.4 Hz, 1H), 3.80 (s, 3H), 2.23 – 2.11 (m, 1H), 2.10 – 2.04 (m, 1H), 2.03 (d, *J* = 1.2 Hz, 3H), 1.94 – 1.83 (m, 1H), 1.84 – 1.77 (m, 1H), 1.76 – 1.68 (m, 1H), 1.67 – 1.59 (m, 1H), 1.23 (d, *J* = 6.5 Hz, 3H), 1.07 – 1.00 (m, 21H), 0.87 (s, 9H), 0.86 (s, 9H), 0.08 (s, 3H), 0.07 (s, 3H), 0.06 (s, 3H), 0.05 (s, 3H). ^{13}C NMR (101 MHz, $CDCl_3$) δ 170.2, 159.3, 151.7, 133.8, 130.4, 129.9, 129.7, 113.9, 106.7, 92.6, 87.9, 85.0, 81.6, 81.1, 80.2, 79.5, 76.1, 70.5, 69.3, 55.4, 34.3, 31.2, 26.0, 25.9, 24.3, 18.21, 18.16, 18.13, 18.07, 18.0, 16.6, 12.4, –4.1, –4.2, –4.3, –4.7. IR (film): $\tilde{\nu}$ 2926, 2856, 1734, 1463, 1380, 1251, 1082, 882, 833, 778 cm^{-1}. HRMS (ESI$^+$) calcd. for $C_{48}H_{82}O_8Si_3Na$ $[M+Na]^+$: 893.52097; found: 893.52104.

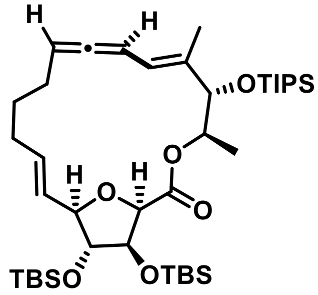

Compound 22. The gold complex **26** (0.25 mL of a stock solution prepared from 1.0 mg in 1 mL dichloromethane; 0.25 mg, 0.0003 mmol) was added to a solution of compound **21** (2.5 mg, 0.0029 mmol) in dichloromethane (0.75 mL). The resulting solution was stirred at 40 °C (bath temperature) for 12 h before the mixture was filtered through a short plug of silica, eluting with ethyl acetate. The filtrate was concentrated under reduced pressure, and the residue was purified *via* flash column chromatography eluting with *tert*-butyl methyl ether/hexane (0→1% *tert*-butyl methyl ether by volume) to give the title compound as a colorless oil. The product was contaminated with some silicon grease, but the yield was indicated to be 0.017 mmol by ^{1}H NMR (60%). $[\alpha]_D^{20}$= –23.0 (c = 0.13, $CHCl_3$). ^{1}H NMR (600 MHz, $CDCl_3$) δ 5.92 (ddt, *J* = 11.0, 5.6, 2.4 Hz, 1H), 5.86 (d, *J* = 11.0 Hz, 1H), 5.53 (ddd, *J* = 15.0, 9.0, 2.0 Hz, 1H), 5.45 (ddd, *J* = 15.0, 10.8, 3.5 Hz, 1H), 5.09 (q, *J* = 6.8 Hz, 1H), 4.92 (dq, *J* = 9.4, 5.6 Hz, 1H), 4.37 (d, *J* = 8.1 Hz, 1H), 4.27 (t, *J* = 8.1 Hz, 1H), 4.18 (t, *J* = 8.1 Hz, 1H), 4.12 (d, *J* = 9.0 Hz, 1H), 3.95 (t, *J* = 8.6 Hz, 1H), 2.14 – 2.05 (m, 2H), 1.62 (t, *J* = 1.2 Hz, 3H), 1.59 – 1.50 (m, 2H), 1.40 (d, *J* = 6.8 Hz, 3H), 1.31 – 1.27 (m, 2H), 1.11 – 1.01 (m, 21H), 0.89 (s, 9H), 0.88 (s, 9H), 0.12 (s, 3H), 0.09 (s, 3H), 0.04 (s, 3H), 0.01 (s, 3H). ^{13}C NMR (151 MHz, $CDCl_3$) δ 209.3, 169.8, 135.5, 135.4, 131.4, 125.8, 90.3, 89.7, 83.4, 82.5, 79.1, 78.8, 77.7, 71.1, 29.8, 27.0, 26.4, 26.04, 26.01, 18.8, 18.3, 18.2, 18.15, 18.10, 10.7, 1.2, -3.5, -3.7, -4.49, -4.51. IR (film): $\tilde{\nu}$ 2929, 2857, 1737, 1464, 1257, 1084, 1011, 837 cm^{-1}. HRMS (ESI^+) calcd. for $C_{40}H_{74}O_6Si_3Na$ $[M+Na]^+$: 757.46855; found: 757.46871.

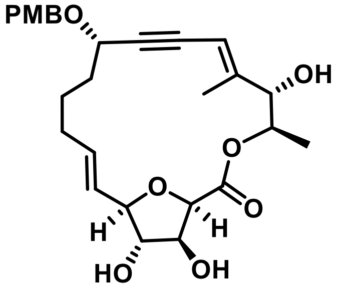

Compound 23. Compound **21** (25.0 mg, 0.028 mmol) was dissolved in THF (2 mL), and the resulting solution was cooled to 0 °C. TBAF (1.0 M in THF, 0.30 mL, 0.30 mmol) was added dropwise, and the resulting solution was warmed to ambient temperature over 45 min. Saturated aqueous ammonium chloride (3 mL) was then added, and the mixture was diluted with water (3 mL), brine (3 mL) and ethyl acetate (5 mL). The organic layer was separated, and the aqueous phase was extracted three times with ethyl acetate (10 mL). The combined organic extracts were dried over anhydrous sodium sulfate, and filtered. The filtrate was concentrated under reduced pressure, and the residue was purified *via* flash chromatography eluting with ethanol/*tert*-butyl methyl ether (0→10% ethanol by volume) to give the title compound as a colorless oil (11.1 mg, 78% yield). $[\alpha]_D^{20}$= –18.4 (c = 0.25, $CHCl_3$). ^{1}H NMR (600 MHz, $CDCl_3$): δ 7.30 – 7.26

(m, 2H), 6.90 – 6.85 (m, 2H), 5.93 (dddd, *J* = 15.5, 8.2, 4.7, 1.3 Hz, 1H), 5.62 – 5.58 (m, 1H), 5.59 – 5.57 (m, 1H), 5.11 (p, *J* = 6.4 Hz, 1H), 4.72 (d, *J* = 5.6 Hz, 1H), 4.70 (d, *J* = 11.4 Hz, 1H), 4.42 (d, *J* = 11.4 Hz, 1H), 4.35 (dd, *J* = 5.6, 3.7 Hz, 1H), 4.34 – 4.31 (m, 1H), 4.28 (dt, *J* = 7.3, 2.7 Hz, 1H), 4.08 – 4.05 (m, 2H), 3.80 (s, 3H), 2.24 – 2.17 (m, 1H), 2.07 – 2.02 (m, 1H), 2.01 (d, *J* = 1.2 Hz, 3H), 1.91 – 1.83 (m, 1H), 1.81 – 1.73 (m, 1H), 1.72 – 1.67 (m, 1H), 1.67 – 1.62 (m, 1H), 1.41 (d, *J* = 6.4 Hz, 3H). ^{13}C NMR (151 MHz, $CDCl_3$): δ 169.3, 159.4, 149.5, 133.7, 130.3, 129.7, 128.7, 113.9, 109.5, 93.7, 85.9, 83.3, 80.7, 80.3, 79.1, 78.3, 73.6, 70.4, 69.1, 55.4, 33.9, 30.5, 23.2, 18.0, 15.5. IR (film): $\tilde{\nu}$ 3395 (br), 2925, 2857, 1742, 1513, 1302, 1248, 1175, 1036, 973, 823 cm^{-1}. HRMS (ESI$^+$) calcd. for $C_{27}H_{34}O_8Na$ $[M+Na]^+$: 509.21459; found: 509.21489.

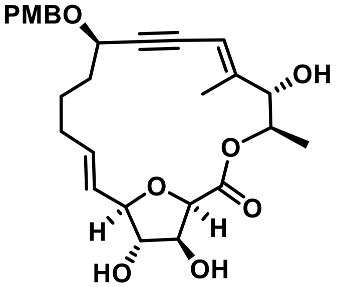

Compound 11-*epi*-23. Prepared analogously from compound **11-*epi*-21** (15.0 mg, 0.017 mmol); colorless oil (5.1 mg, 0.010 mmol, 60% yield). $[\alpha]_D^{20}$= 2.3 (c = 0.13, $CHCl_3$). ^{1}H NMR (400 MHz, $CDCl_3$): δ 7.32 – 7.26 (m, 2H), 6.93 – 6.82 (m, 2H), 5.94 (dddd, *J* = 15.6, 8.1, 4.6, 1.4 Hz, 1H), 5.63 (ddt, *J* = 15.6, 6.2, 1.5 Hz, 1H), 5.60 (br s, 1H), 5.10 (p, *J* = 6.5 Hz, 1H), 4.71 (d, *J* = 5.6 Hz, 1H), 4.69 (d, *J* = 11.4 Hz, 1H), 4.43 (d, *J* = 11.4 Hz, 1H), 4.37 – 4.30 (m, 2H), 4.20 – 4.13 (m, 1H), 4.08 (t, *J* = 3.6 Hz, 1H), 4.03 (d, *J* = 6.5 Hz, 1H), 3.80 (s, 3H), 2.30 – 2.16 (m, 1H), 2.10 – 1.98 (m, 1H), 1.96 (d, *J* = 1.2 Hz, 3H), 1.81 – 1.49 (m, 4H), 1.40 (d, *J* = 6.5 Hz, 3H). ^{13}C NMR (101 MHz, $CDCl_3$): δ 169.3, 159.4, 148.6, 133.3, 130.1, 129.8, 128.6, 113.9, 109.6, 94.0, 85.8, 83.4, 80.9, 80.2, 79.1, 78.3, 73.3, 70.5, 69.3, 55.4, 34.3, 30.6, 23.3, 18.0, 15.2. IR (film): $\tilde{\nu}$ 3400 (br), 2926, 2856, 1740, 1514, 1302, 1251, 1175, 1036, 977, 823 cm^{-1}. HRMS (ESI$^+$) calcd. for $C_{27}H_{34}O_8Na$ $[M+Na]^+$: 509.21459; found: 509.21486.

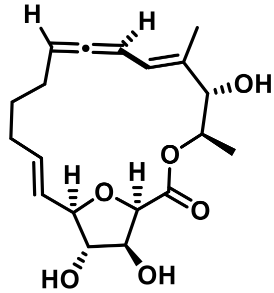

Archangiumide (1).[13] The gold complex **26** (3.1 mg, 0.004 mmol) was added to a solution of compound **23** (11.1 mg, 0.023 mmol) in dichloromethane (1 mL). The resulting mixture was stirred at 40 °C (bath temperature) for 12 h before the mixture was filtered through a short plug of silica, eluting with ethyl acetate. The filtrate was concentrated under reduced pressure, and the residue was purified *via* flash chromatography eluting with ethanol/*tert*-butyl methyl ether (0→10% ethanol by volume) to give the title compound as a colorless oil which solidified upon standing (4.5 mg, 58% yield).

A sample prepared from epimeric starting material **11-*epi*-23** was identical in all respects.

$[\alpha]_D^{20}$= 41.0 (c = 0.20, MeOH) (lit.:[13] $[\alpha]_D^{25}$= 37.0, c = 0.05, MeOH). ^1H NMR (600 MHz, [D_6]-DMSO) δ 6.03 (dddd, *J* = 11.4, 5.6, 3.6, 1.8 Hz, 1H), 5.83 (d, *J* = 11.2 Hz, 1H), 5.60 (d, *J* = 4.7 Hz, 1H), 5.54 (ddd, *J* = 15.3, 8.4, 1.7 Hz, 1H), 5.39 (ddd, *J* = 15.0, 10.7, 4.0 Hz, 1H), 5.25 (d, *J* = 4.6 Hz, 1H), 5.22 (d, *J* = 6.0 Hz, 1H), 5.17 (dt, *J* = 10.2, 5.6 Hz, 1H), 4.67 (dq, *J* = 9.9, 6.0 Hz, 1H), 4.25 (d, *J* = 8.4 Hz, 1H), 4.14 (td, *J* = 8.4, 4.7 Hz, 1H), 3.85 (dd, *J* = 9.9, 4.6 Hz, 1H), 3.83 (t, *J* = 8.4 Hz, 1H), 3.80 (td, *J* = 8.4, 6.0 Hz, 1H), 2.23 – 2.17 (m, 1H), 2.04 – 2.00 (m, 2H), 1.99 – 1.92 (m, 1H), 1.53 (t, *J* = 1.2 Hz 1H), 1.52 – 1.48 (m, 1H), 1.48 – 1.41 (m, 1H), 1.23 (d, *J* = 6.0 Hz, 1H). ^{13}C NMR (151 MHz, [D_6]-DMSO) δ 208.3, 170.0, 135.9, 132.6, 131.9, 124.0, 90.1, 89.9, 82.9, 79.5, 79.0, 78.0, 76.1, 69.8, 29.4, 26.3, 26.0, 18.0, 10.1. IR (film): $\tilde{\nu}$ 3372 (br), 2929, 1727, 1375, 1352, 1299, 1262, 1115, 1045, 1027 cm^{-1}. HRMS (ESI^+) calcd. for $C_{19}H_{26}O_6Na$ $[M+Na]^+$: 373.16216; found: 373.16234.

Table S1. NMR data ([D_6]-DMSO, 600 MHz) of synthetic archangiumide (1)

[NOTE: The highlighted coupling (H16-H17) constant disagrees with that reported in the literature, but is consistent with the other coupling constants observed within the spectrum. We attribute this apparent disparity to the higher resolution of a 600 MHz spectrum, enabling a more accurate assignment of a multiplet which overlaps the neighboring resonances].

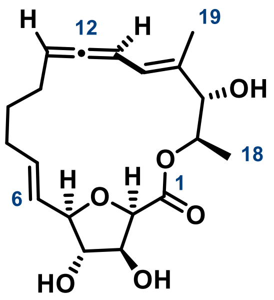

Numbering scheme as shown in the Insert.

Atom	δ (ppm)	*J* (Hz)	COSY	HSQC	HMBC	NOESY
**1 C**	170.0				3, 17	
**2 C**	79.01			2’	3, 3’	
**2’ H**	4.25	8.4	3, 5’	2	3, 4, 5	
**3 C**	76.1			3	2’, 3’, 4, 4’	
**H**	4.14	8.4, 4.7	2’, 3’, 4	3	1, 2, 4	3’, 4’, 5’
**3’ O**						
**H**	5.60	4.7	3		2, 3, 4	3, 4
**4 C**	78.0			4	2’, 3, 3’, 4’, 5’	
**H**	3.80	8.4, 6.0	3, 4’, 5’	4	3, 5	3’, 4’, 6, 18
**4’ O**						
**H**	5.22	6.0	4		3, 4, 5	3, 4, 5’
**5 C**	82.9			5’	2’, 4, 4’, 7	
**5’ H**	3.83	8.3	2’, 4, 6, 7	5	4, 7	3, 4’, 7
**6 C**	131.9			6	7	
**H**	5.54	15.3, 8.4, 1.7	5’, 7, 8b	6	7, 8	4, 8a, 14
**7 C**	132.6			7	5’, 6	
**H**	5.39	15.0, 10.7, 4.0	5’, 6, 8a, 8b	7	5, 6	5’, 8b
**8 C**	29.4			8a, 8b	6	
**Ha**	2.20		7, 8b, 9a, 9b	8		6
**Hb**	2.02		6, 7, 8a, 9a, 9b	8		7
**9 C**	26.0			9a, 9b		

**Ha**	1.50		8a, 8b, 9b, 10a, 10b	9		
**Hb**	1.45		8a, 8b, 9a, 10a	9		
**10 C**	26.3			10a, 10b		
**Ha**	2.03		9a, 9b, 10b, 11, 13’	10		11
**Hb**	1.95		9a, 10a, 11, 13’	10		
**11 C**	90.1			11		
**H**	5.17	10.2, 5.6	10a, 10b, 13’, 14, 19	11		10a
**12 C**	208.3				14	
**13 C**	89.9			13’	14	
**13’ H**	6.03	11.2, 5.6, 3.6, 1.8	10a, 10b, 11, 14	13		19
**14 C**	124.0			14	16, 19	
**H**	5.83	11.2	11, 13’, 16, 19	14	12, 13, 16, 19	6, 16
**15 C**	135.9				16’, 17, 19	
**16 C**	79.5			16	14, 16’, 17, 18, 19	
**H**	3.85	9.9, 4.6	14, 16’, 17, 18	16	14, 17, 18, 19	14, 16’, 18
**16’ O**						
**H**	5.25	4.6	16		15, 16, 17	16, 17, 18, 19
**17 C**	69.8			17	16, 16’, 18	
**H**	4.67	9.9, 6.0	16, 18	17	1, 15, 16	16’, 19
**18 C**	18.0			18	16	
**H3**	1.23	6.0	16, 17	18	16, 17	4, 16, 16’
**19 C**	10.1			19	14, 16	
**H3**	1.53	1.2	11, 14	19	14, 15, 16	13’, 16’, 17

Table S2. ^{1}H NMR data comparison between synthetic archangiumide (1) and literature data[13]

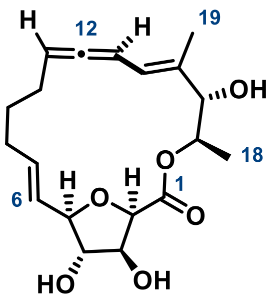

Spectra recorded in [D_6]-DMSO on a 600 MHz spectrometer (lit.:[13] 500 MHz). Highlighted peaks in the literature data correspond to OH resonances, which were not given in tabulated form in the isolation paper; these values were obtained by inspecting the picked peaks in the NMR spectra in the supplementary information of that paper and averaging the two values corresponding to the doublet peaks in each case. For resonances assigned as complex multiplets, the midpoint of the peak was taken as the ppm value.

Numbering scheme as shown in the Insert.

Atom	δ (lit.) (ppm)	δ (1) (ppm)	Δδ (lit.-1) (ppm)
**1**	-	-	-
**2**	4.25	4.25	0
**3**	4.14/5.59	4.14/5.60	0/-0.01
**4**	3.81/5.21	3.80/5.22	0.01/-0.01
**5**	3.83	3.83	0
**6**	5.54	5.54	0
**7**	5.39	5.39	0
**8**	2.20/2.02	2.20/2.02	0/0
**9**	1.50/1.43	1.50/1.45	0/-0.02
**10**	2.00/1.94	2.02/1.95	-0.02/-0.01
**11**	5.17	5.17	0
**12**	-	-	-
**13**	6.02	6.03	-0.01
**14**	5.83	5.83	0
**15**	-	-	-
**16**	3.85/5.23	3.85/5.25	0/-0.02
**17**	4.67	4.67	0
**18**	1.23	1.23	0
**19**	1.53	1.53	0

Table S3. **^{13}C NMR data comparison between synthetic archangiumide (**[D$_6$]-DMSO, 151 MHz**) and literature data (**[D$_6$]-DMSO, 125 MHz**).**[13]

Numbering scheme as shown in the Insert.

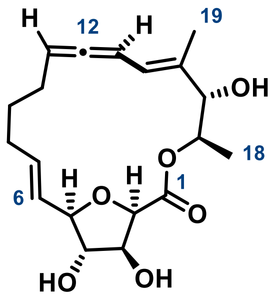

Atom	δ (lit.) (ppm)	δ (1) (ppm)	Δδ (lit.-1) (ppm)
**1**	170.0	170.0	0
**2**	79.0	79.0	0
**3**	76.1	76.1	0
**4**	78.0	78.0	0
**5**	82.9	82.9	0
**6**	131.9	131.9	0
**7**	132.6	132.6	0
**8**	29.4	29.4	0
**9**	26.0	26.0	0
**10**	26.3	26.3	0
**11**	90.1	90.1	0
**12**	208.3	208.3	0
**13**	89.9	89.9	0
**14**	124.0	124.0	0
**15**	135.9	135.9	0
**16**	79.6	79.5	0.01
**17**	69.8	69.8	0
**18**	18.0	18.0	0
**19**	10.1	10.1	0

Figure S1. Spectral ^{13}C NMR data comparison between synthetic archangiumide (1) and literature data[13]

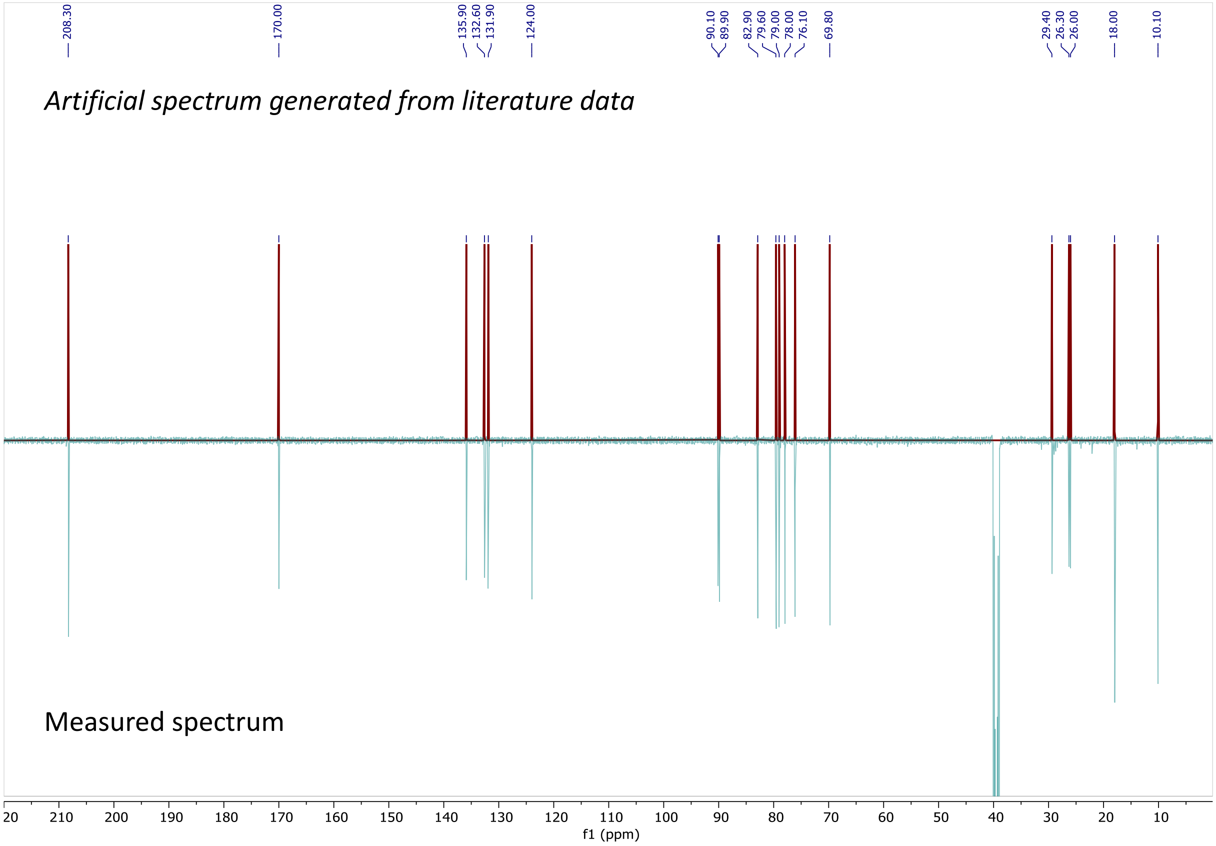

NMR Spectra of New Compounds

Compound 2

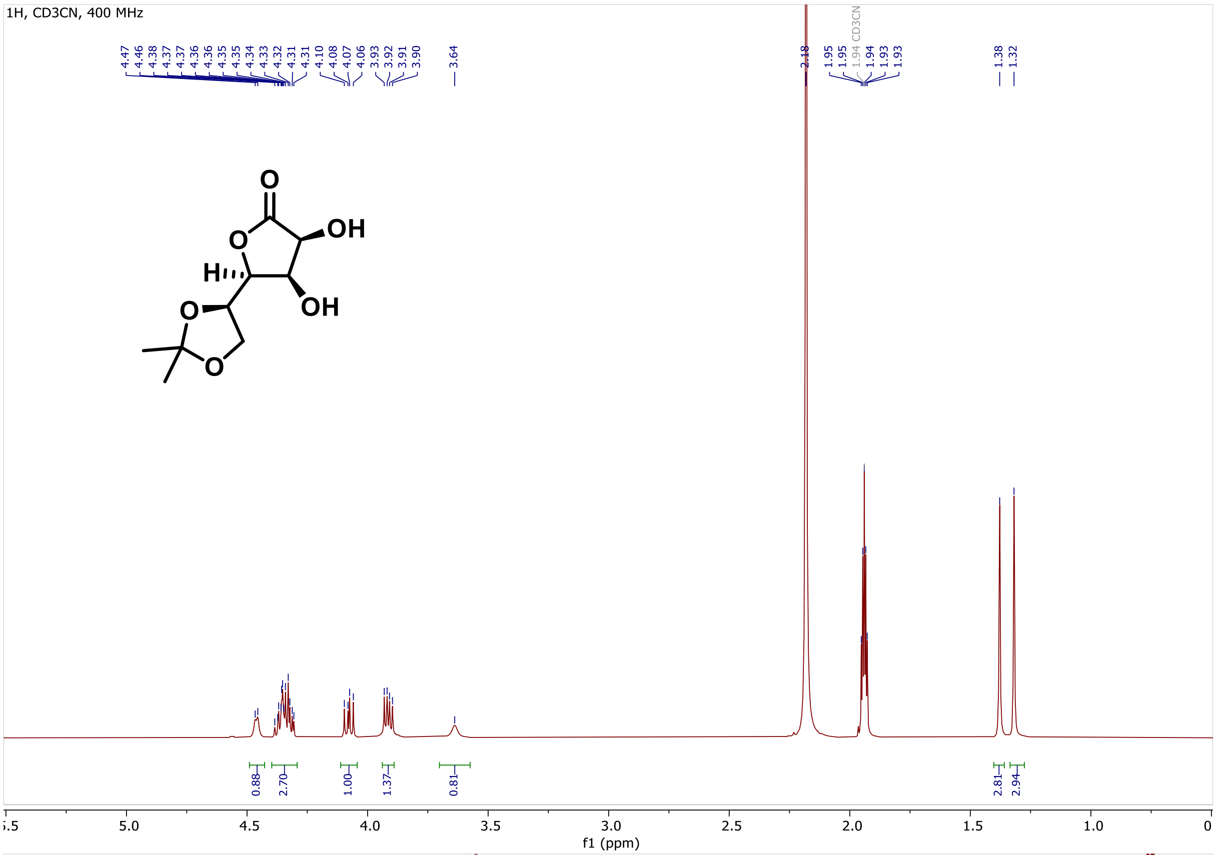

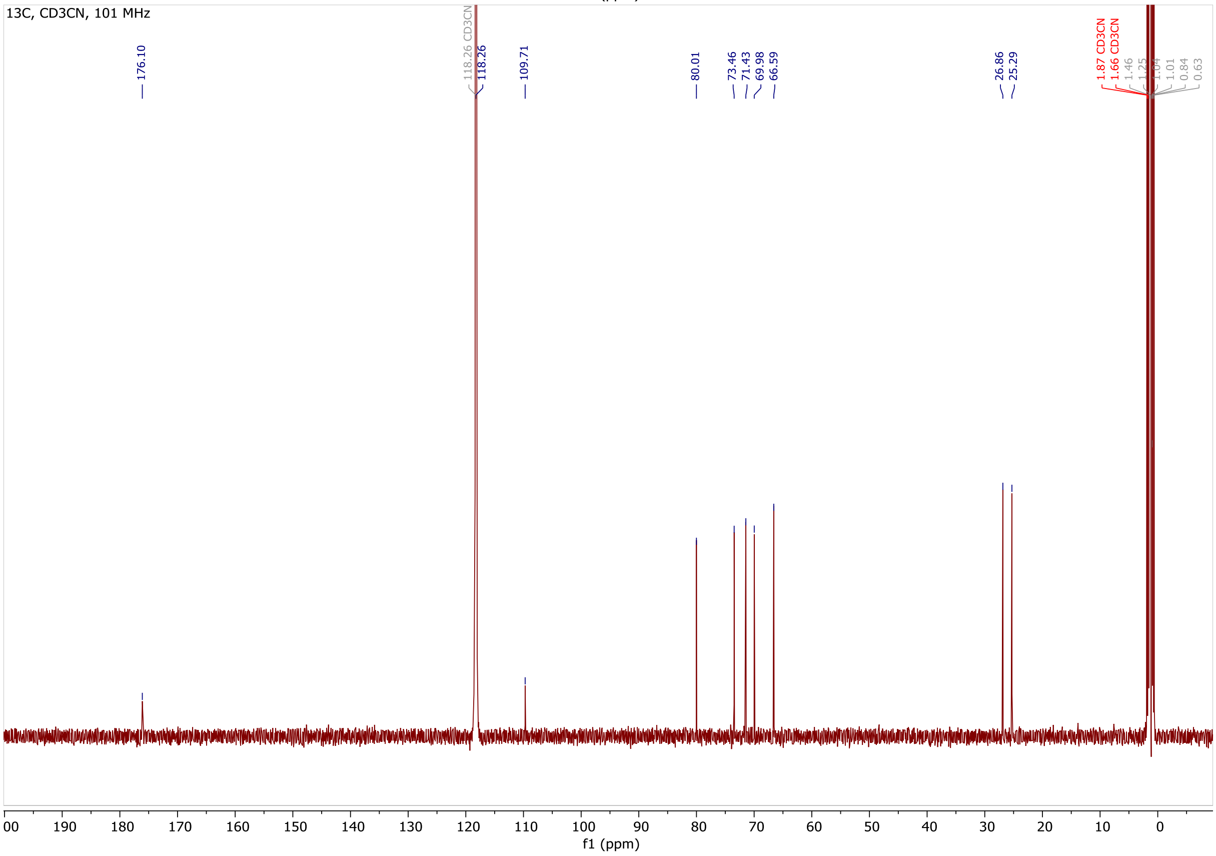

Compound 4

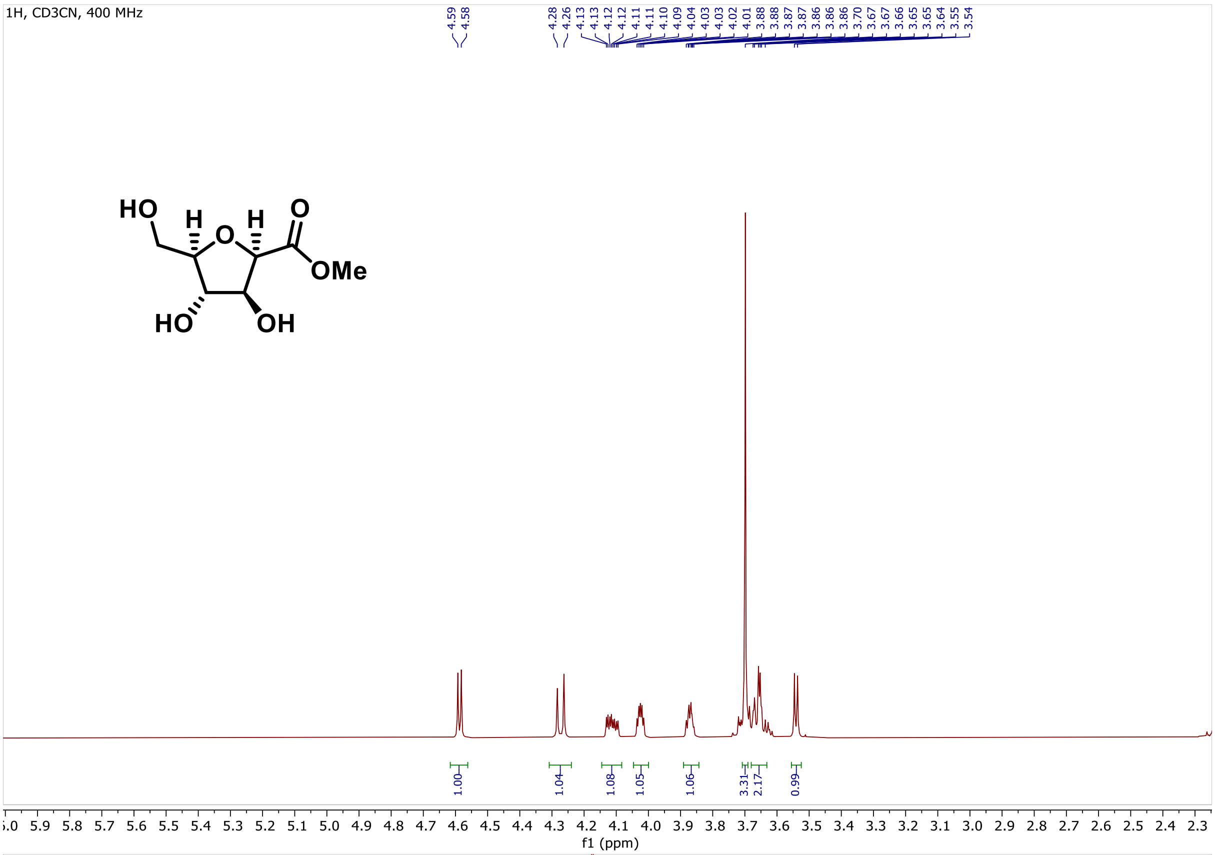

1H, CD3CN, 400 MHz
HO
H
H
O
O
OMe
HO
OH
f1 (ppm)

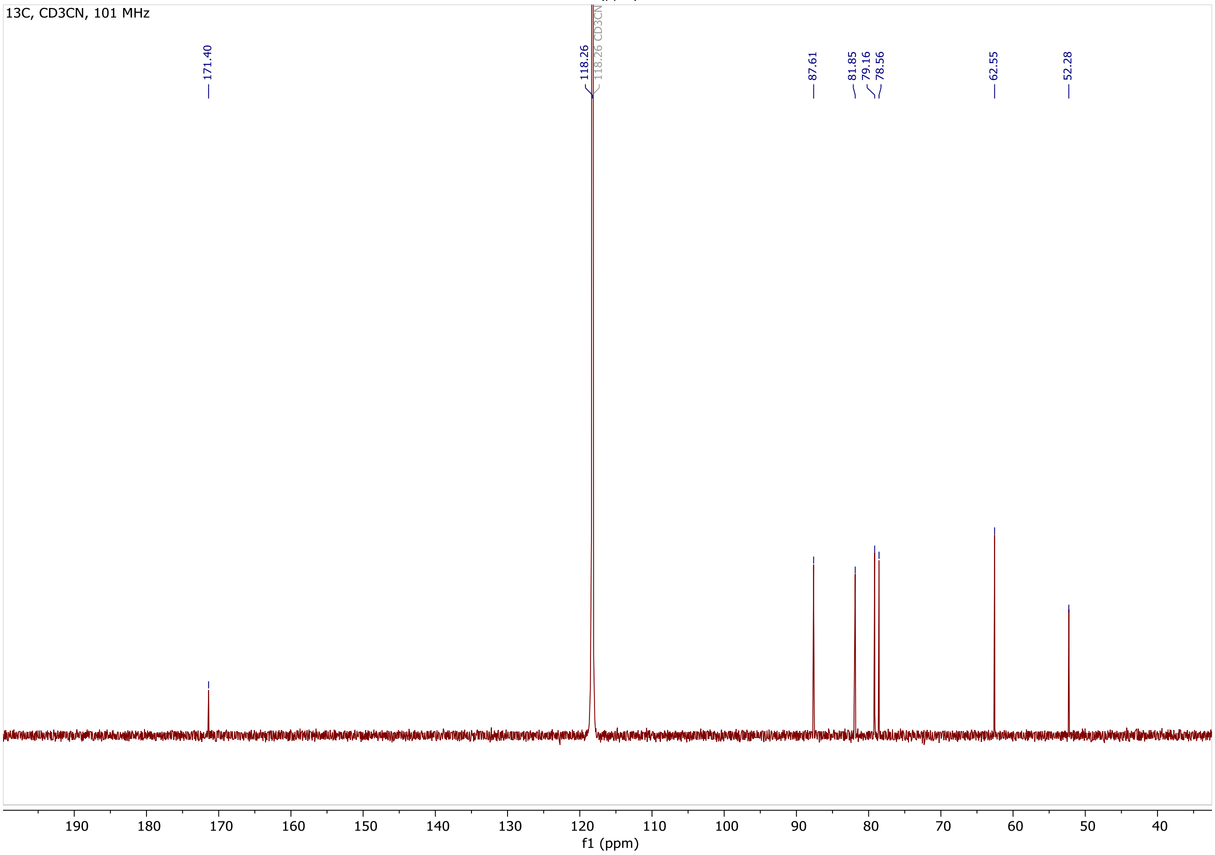

13C, CD3CN, 101 MHz
171.40
118.26
87.61
81.85
79.16
78.56
62.55
52.28
f1 (ppm)

Compound 5

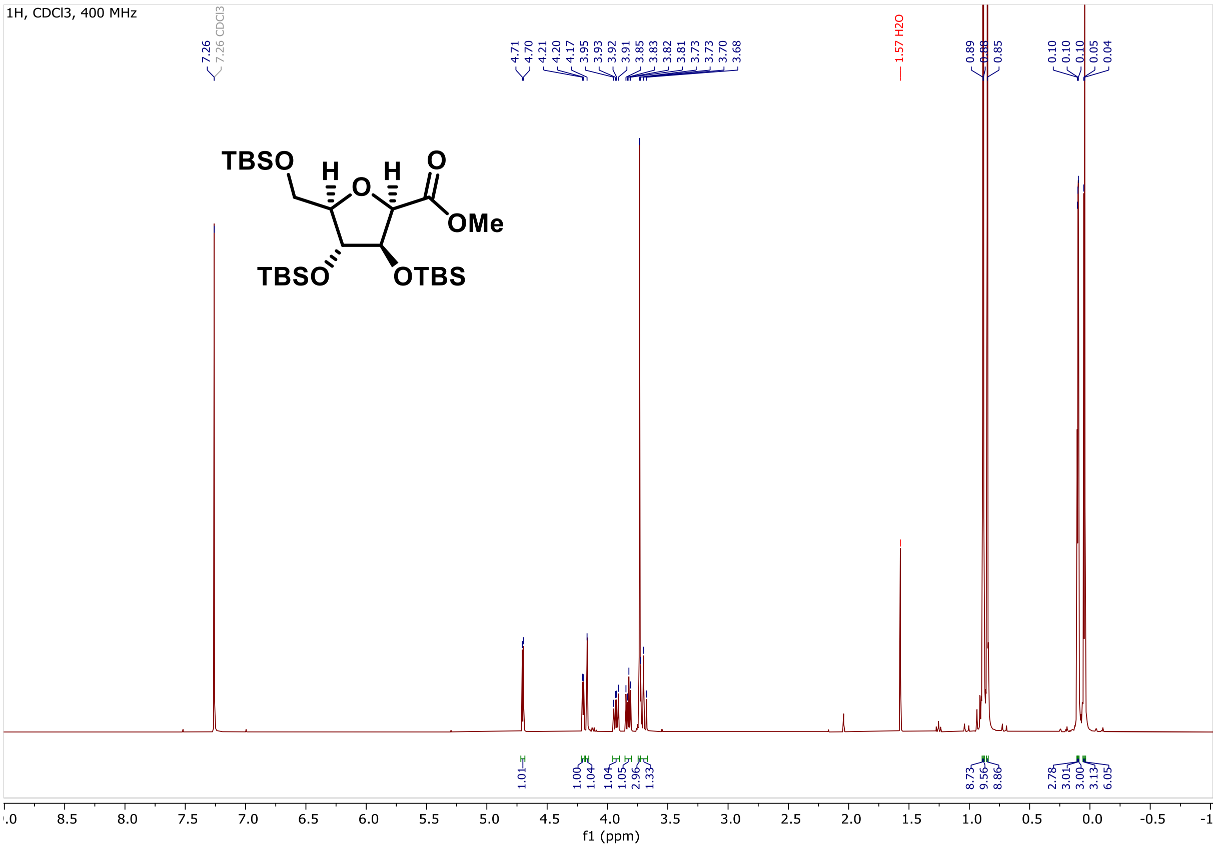

1H, CDCl3, 400 MHz
7.26
7.26 CDCl3
4.71
4.70
4.21
4.20
4.17
3.95
3.93
3.92
3.91
3.85
3.83
3.82
3.81
3.73
3.73
3.70
3.68
1.57 H2O
0.89
0.88
0.85
0.10
0.10
0.10
0.05
0.04
TBSO
H
O
H
O
OMe
TBSO
OTBS
1.01
1.00
1.04
1.04
1.05
2.96
1.33
8.73
9.56
8.86
2.78
3.01
3.00
3.13
6.05
f1 (ppm)

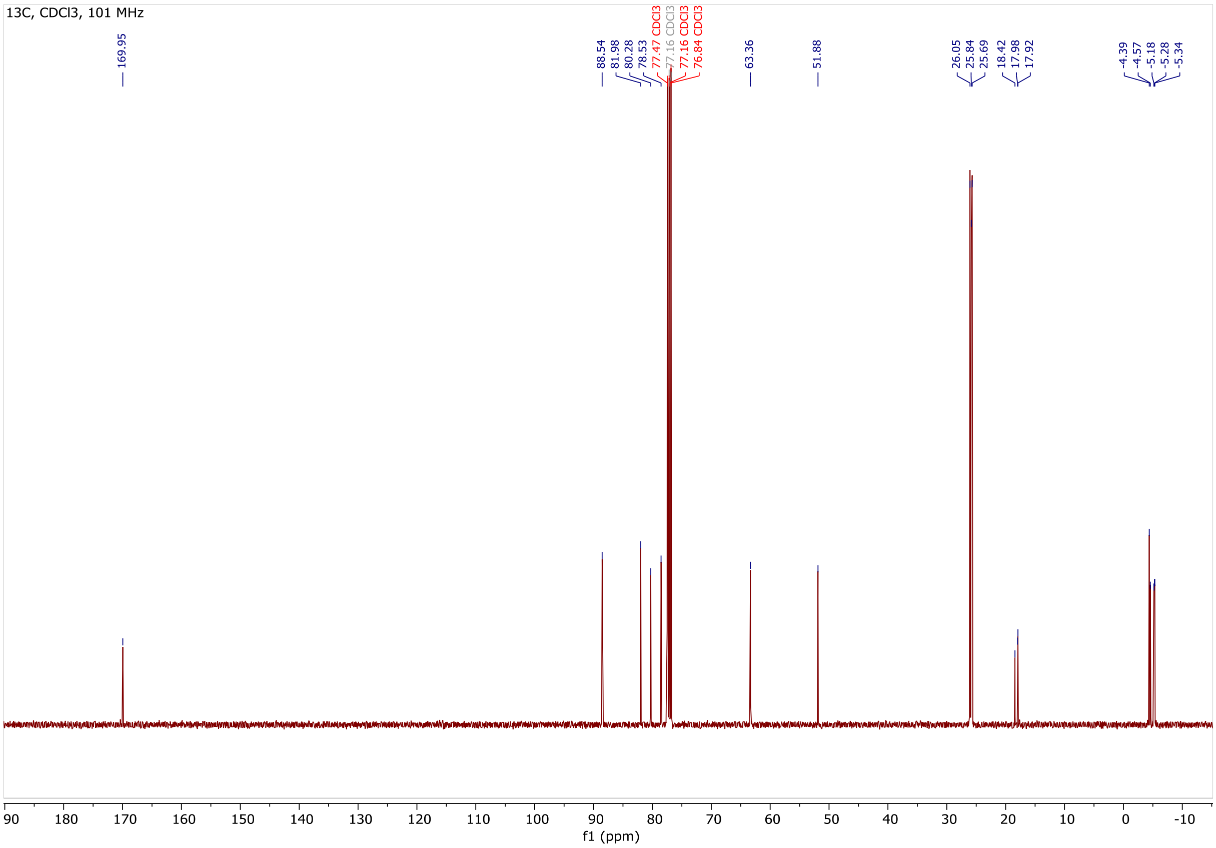

13C, CDCl3, 101 MHz
169.95
88.54
81.98
80.28
78.53
77.47 CDCl3
77.16 CDCl3
77.16 CDCl3
76.84 CDCl3
63.36
51.88
26.05
25.84
25.69
18.42
17.98
17.92
-4.39
-4.57
-5.18
-5.28
-5.34
f1 (ppm)

Compound S1

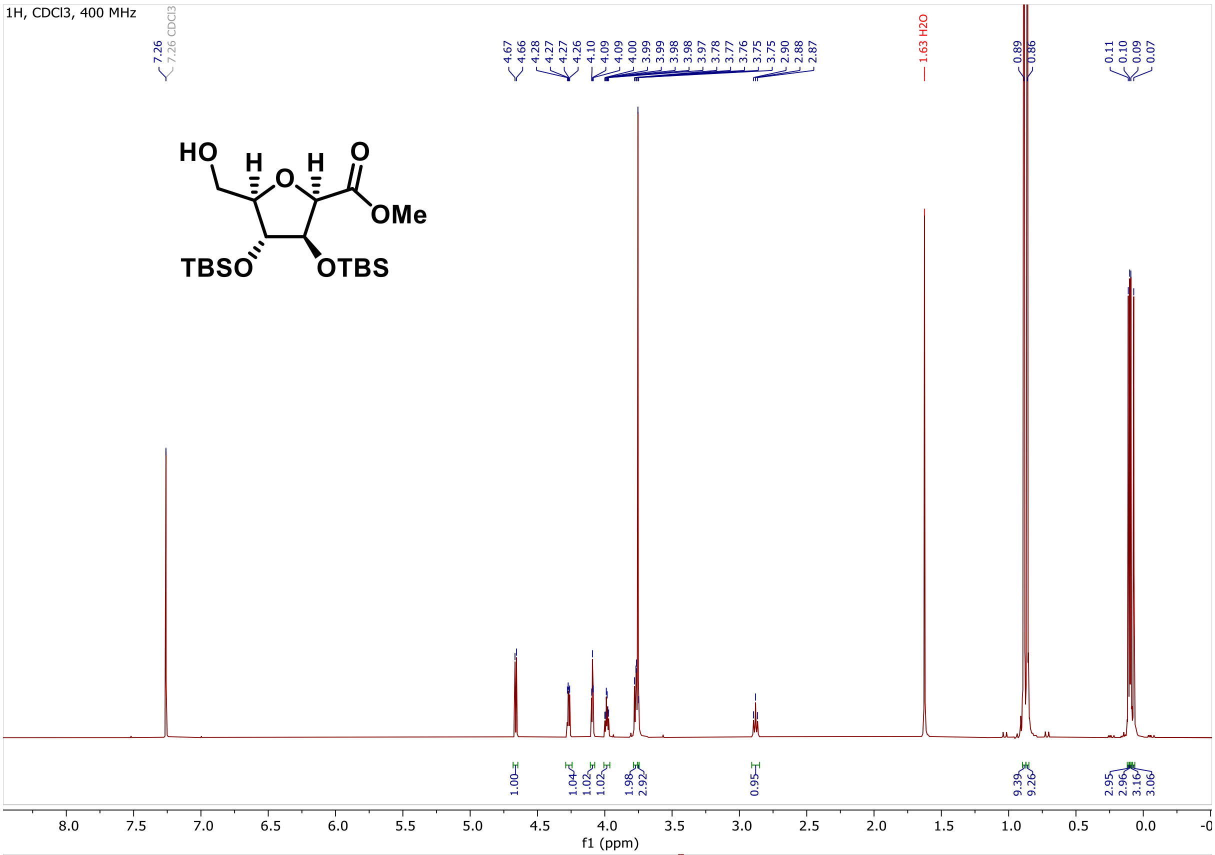

1H, CDCl3, 400 MHz
7.26
7.26 CDCl3
4.67
4.66
4.28
4.27
4.27
4.26
4.10
4.09
4.09
4.00
3.99
3.99
3.98
3.98
3.97
3.78
3.77
3.76
3.75
3.75
2.90
2.88
2.87
1.63 H2O
0.89
0.86
0.11
0.10
0.09
0.07
HO
H
O
H
O
OMe
TBSO
OTBS
1.00
1.04
1.02
1.02
1.98
2.92
0.95
9.39
9.26
2.95
2.96
3.16
3.06
8.0
7.5
7.0
6.5
6.0
5.5
5.0
4.5
4.0
3.5
3.0
2.5
2.0
1.5
1.0
0.5
0.0
f1 (ppm)

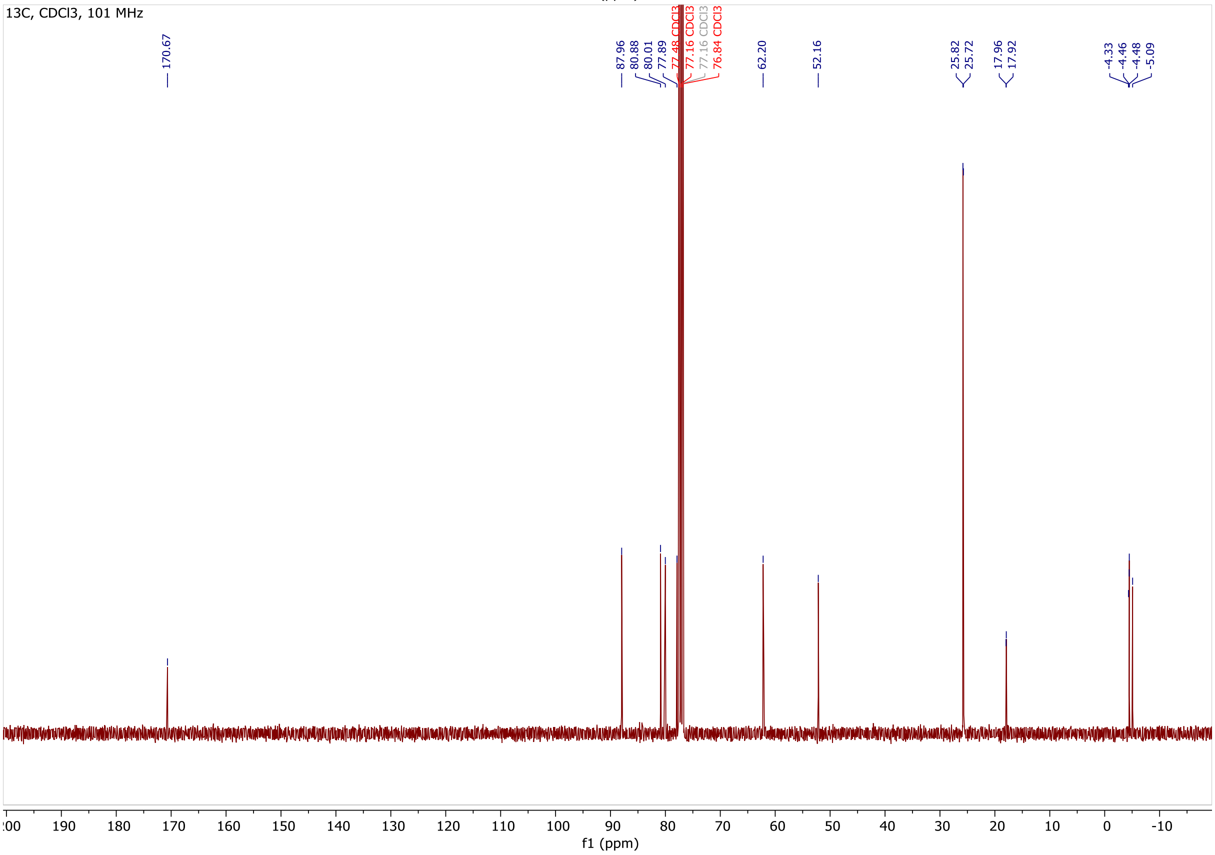

13C, CDCl3, 101 MHz
170.67
87.96
80.88
80.01
77.89
77.48 CDCl3
77.16 CDCl3
77.16 CDCl3
76.84 CDCl3
62.20
52.16
25.82
25.72
17.96
17.92
-4.33
-4.46
-4.48
-5.09
190
180
170
160
150
140
130
120
110
100
90
80
70
60
50
40
30
20
10
0
-10
f1 (ppm)

Compound 6

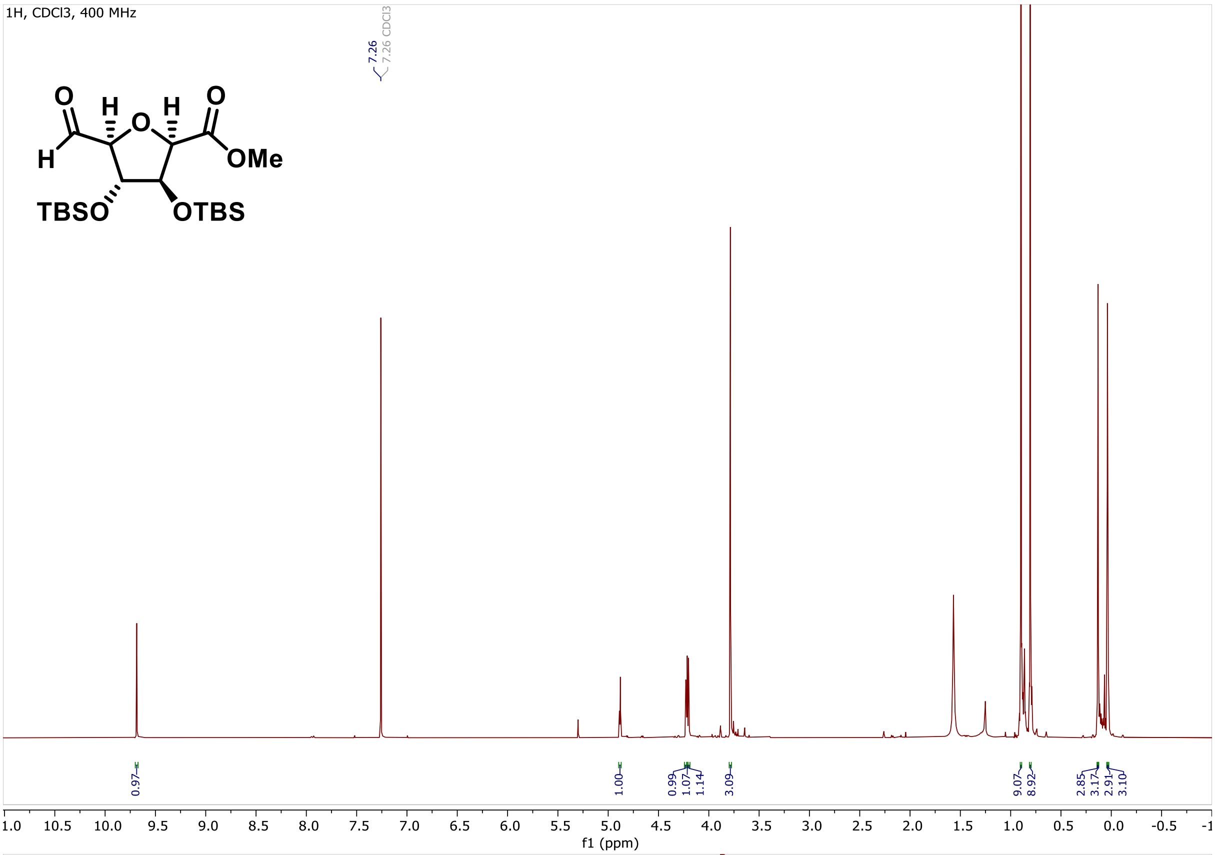
1H, CDCl3, 400 MHz
7.26
7.26 CDCl3
TBSO
OTBS
OMe
0.97
1.00
0.99
1.07
1.14
3.09
9.07
8.92
2.85
3.17
2.91
3.10
f1 (ppm)

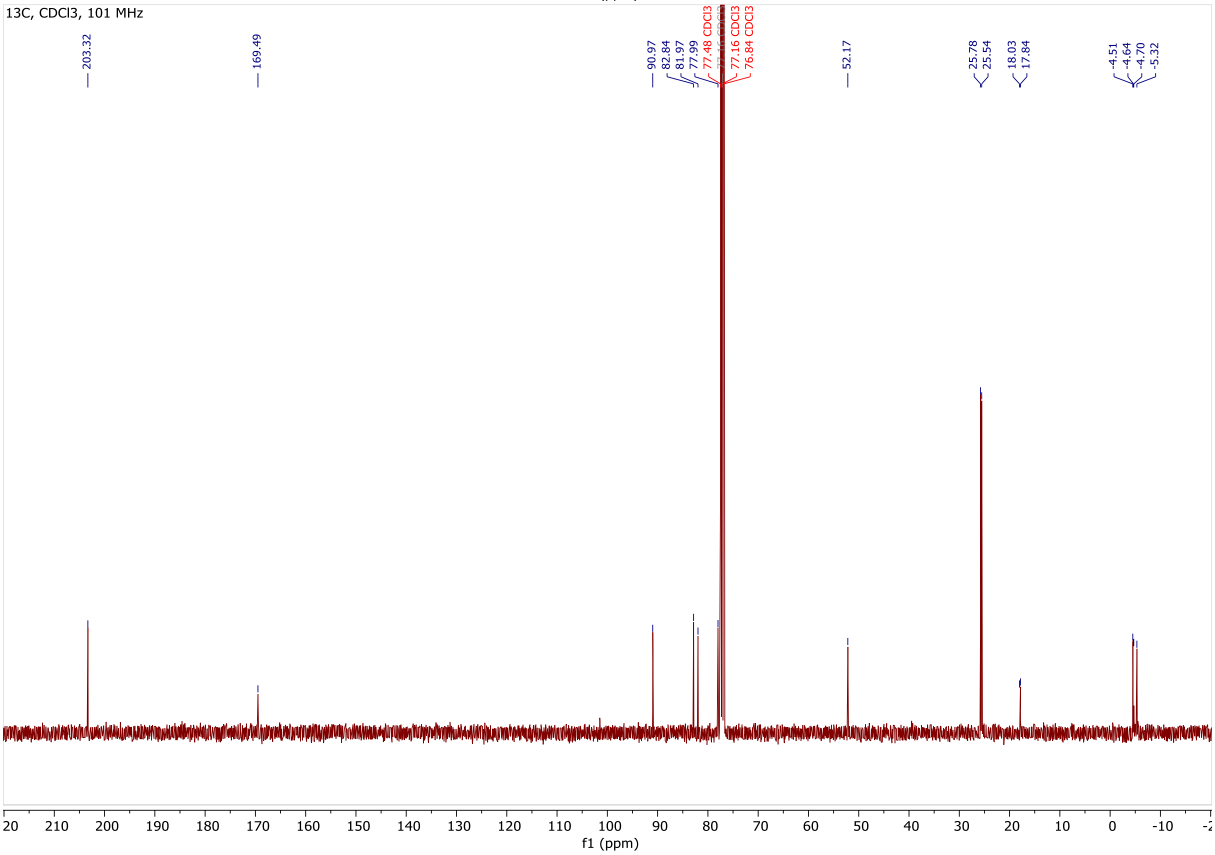
13C, CDCl3, 101 MHz
203.32
169.49
90.97
82.84
81.97
77.99
77.48 CDCl3
77.16 CDCl3
76.84 CDCl3
52.17
25.78
25.54
18.03
17.84
-4.51
-4.64
-4.70
-5.32
f1 (ppm)

Compound 8

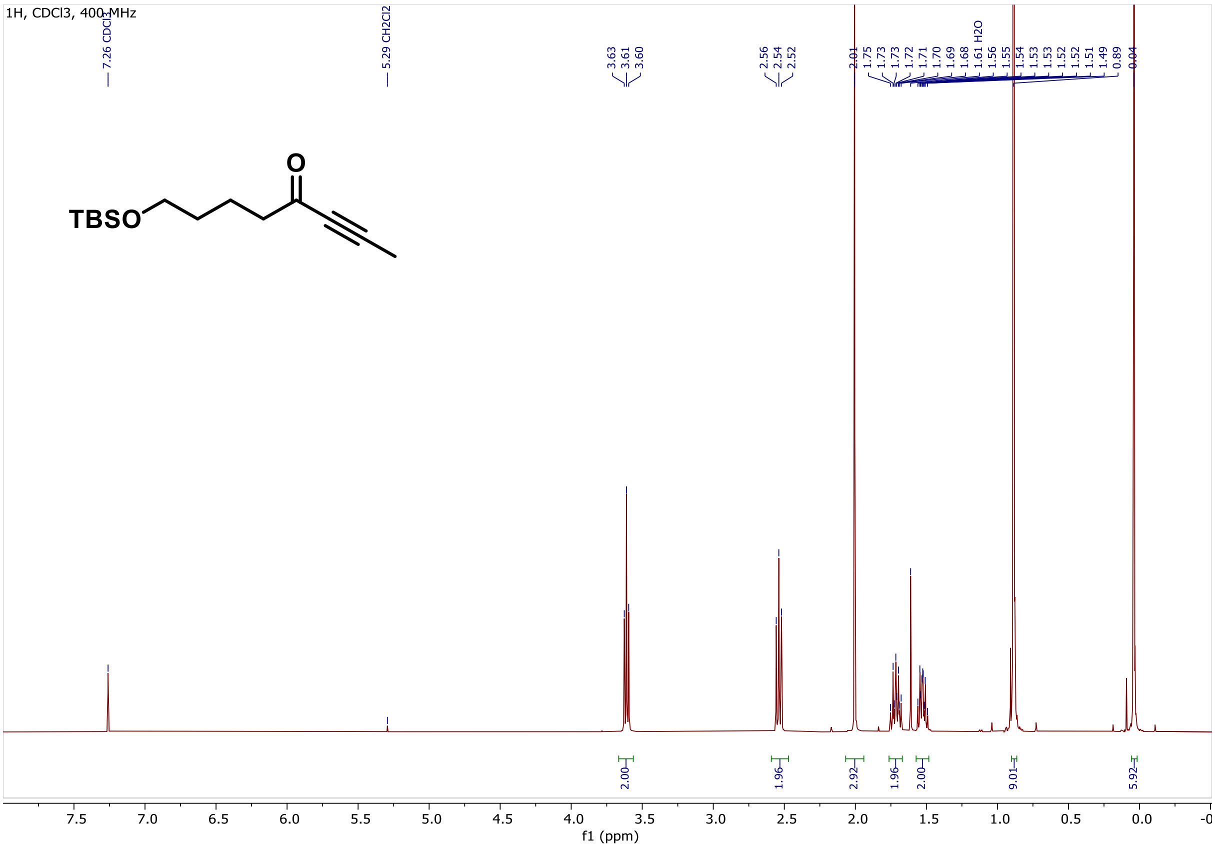
1H, CDCl3, 400 MHz
7.26 CDCl3
5.29 CH2Cl2
3.63
3.61
3.60
2.56
2.54
2.52
2.01
1.75
1.73
1.73
1.72
1.71
1.70
1.69
1.68
1.61 H2O
1.56
1.55
1.54
1.53
1.53
1.52
1.52
1.51
1.49
0.89
0.04
TBSO
O
2.00
1.96
2.92
1.96
2.00
9.01
5.92
7.5
7.0
6.5
6.0
5.5
5.0
4.5
4.0
3.5
3.0
2.5
2.0
1.5
1.0
0.5
0.0
f1 (ppm)

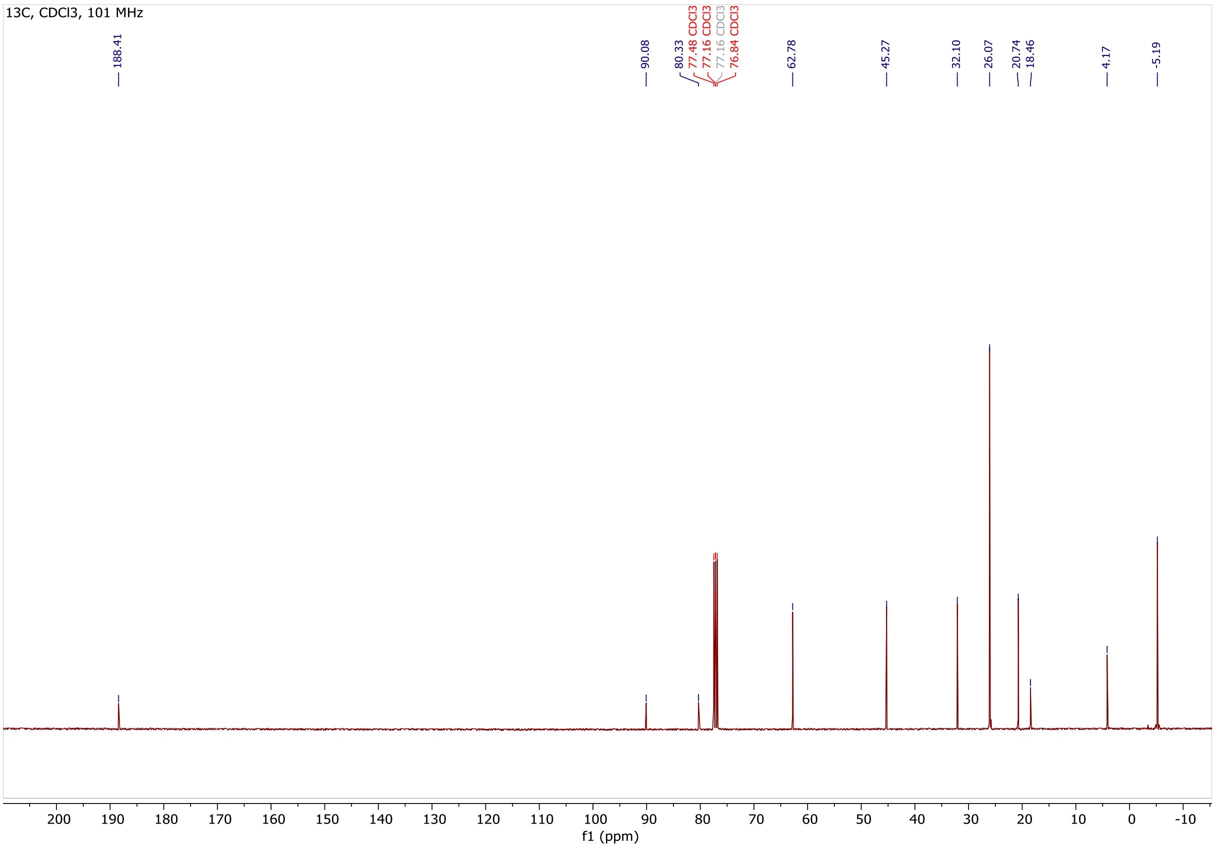
13C, CDCl3, 101 MHz
188.41
90.08
80.33
77.48 CDCl3
77.16 CDCl3
77.16 CDCl3
76.84 CDCl3
62.78
45.27
32.10
26.07
20.74
18.46
-4.17
-5.19
200
190
180
170
160
150
140
130
120
110
100
90
80
70
60
50
40
30
20
10
0
-10
f1 (ppm)

Compound 9

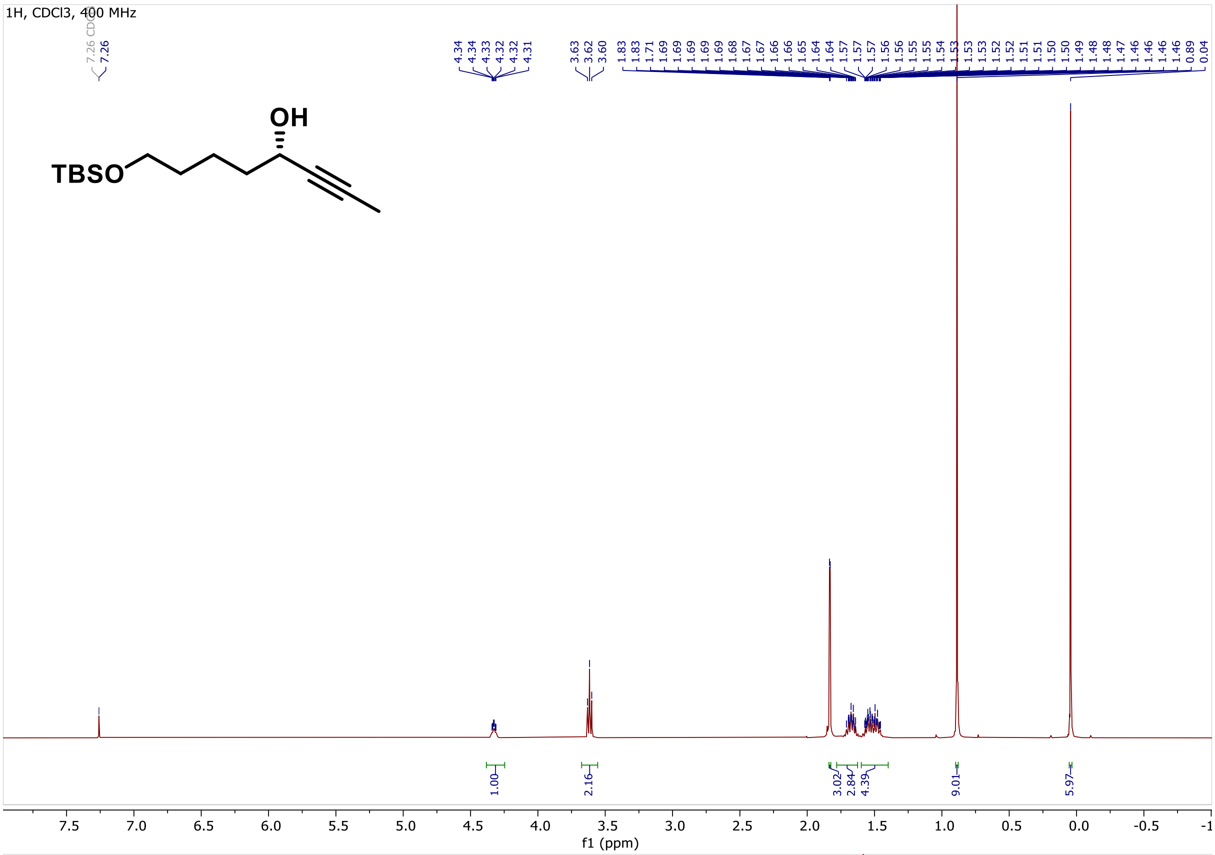
1H, CDCl3, 400 MHz
7.26 CDCl3
7.26
4.34 4.34 4.33 4.32 4.32 4.31
3.63 3.62 3.60
1.83 1.83 1.71 1.69 1.69 1.69 1.69 1.68 1.67 1.67 1.66 1.66 1.65 1.64 1.64 1.57 1.57 1.57 1.56 1.56 1.55 1.55 1.54 1.53 1.53 1.53 1.52 1.52 1.51 1.51 1.50 1.50 1.49 1.48 1.48 1.47 1.46 1.46 1.46 0.89 0.04
OH
TBSO
1.00
2.16
3.02
2.84
4.39
9.01
5.97
7.5 7.0 6.5 6.0 5.5 5.0 4.5 4.0 3.5 3.0 2.5 2.0 1.5 1.0 0.5 0.0 -0.5 -1
f1 (ppm)

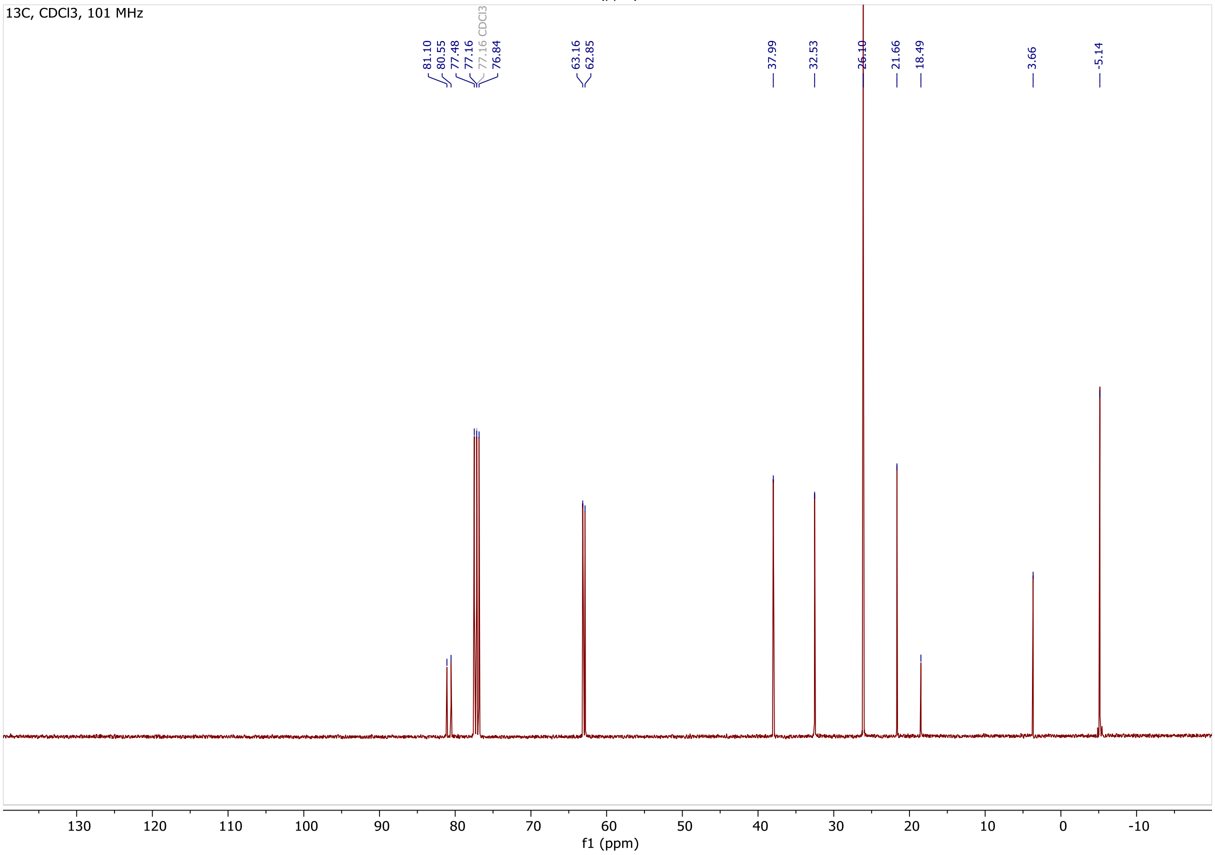
13C, CDCl3, 101 MHz
81.10
80.55
77.48
77.16
77.16 CDCl3
76.84
63.16
62.85
37.99
32.53
26.10
21.66
18.49
3.66
-5.14
130 120 110 100 90 80 70 60 50 40 30 20 10 0 -10
f1 (ppm)

Compound S2

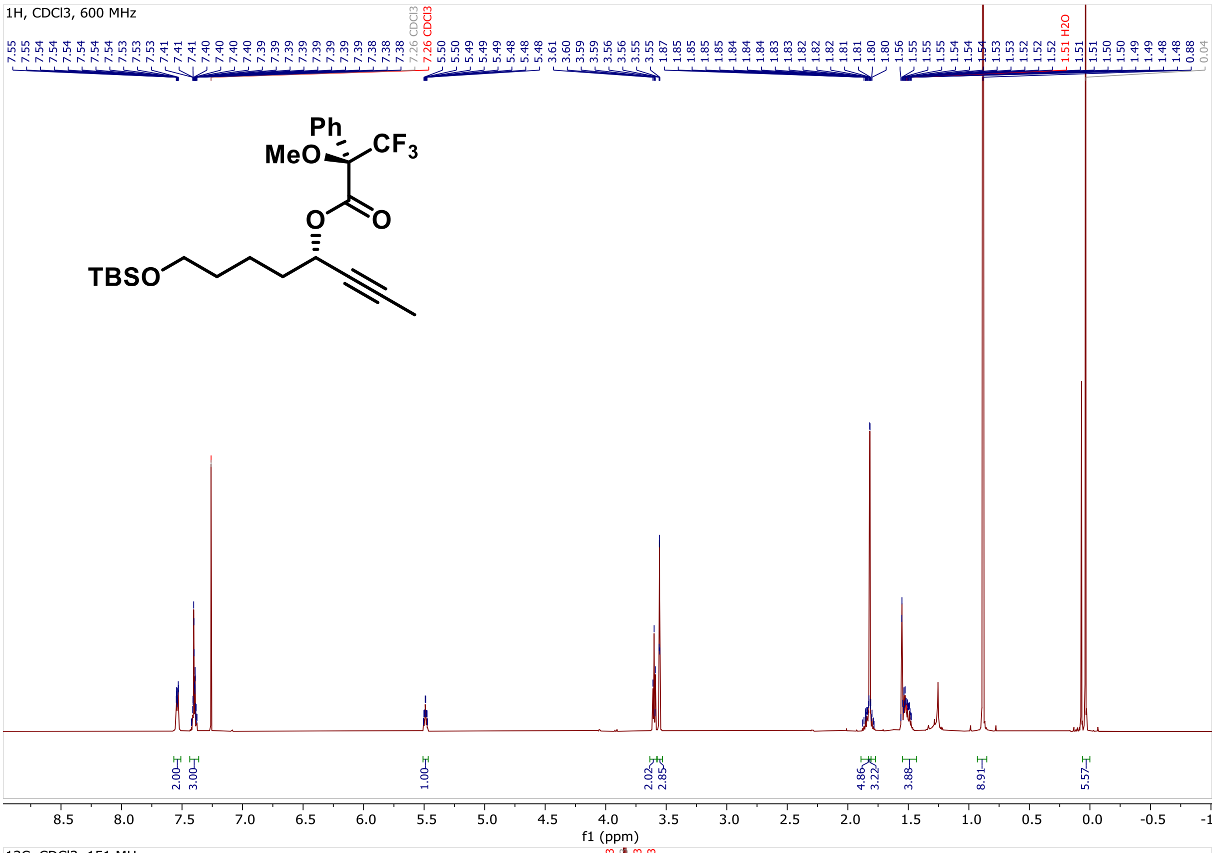

1H, CDCl3, 600 MHz
Ph
MeO
CF3
O
O
TBSO
f1 (ppm)

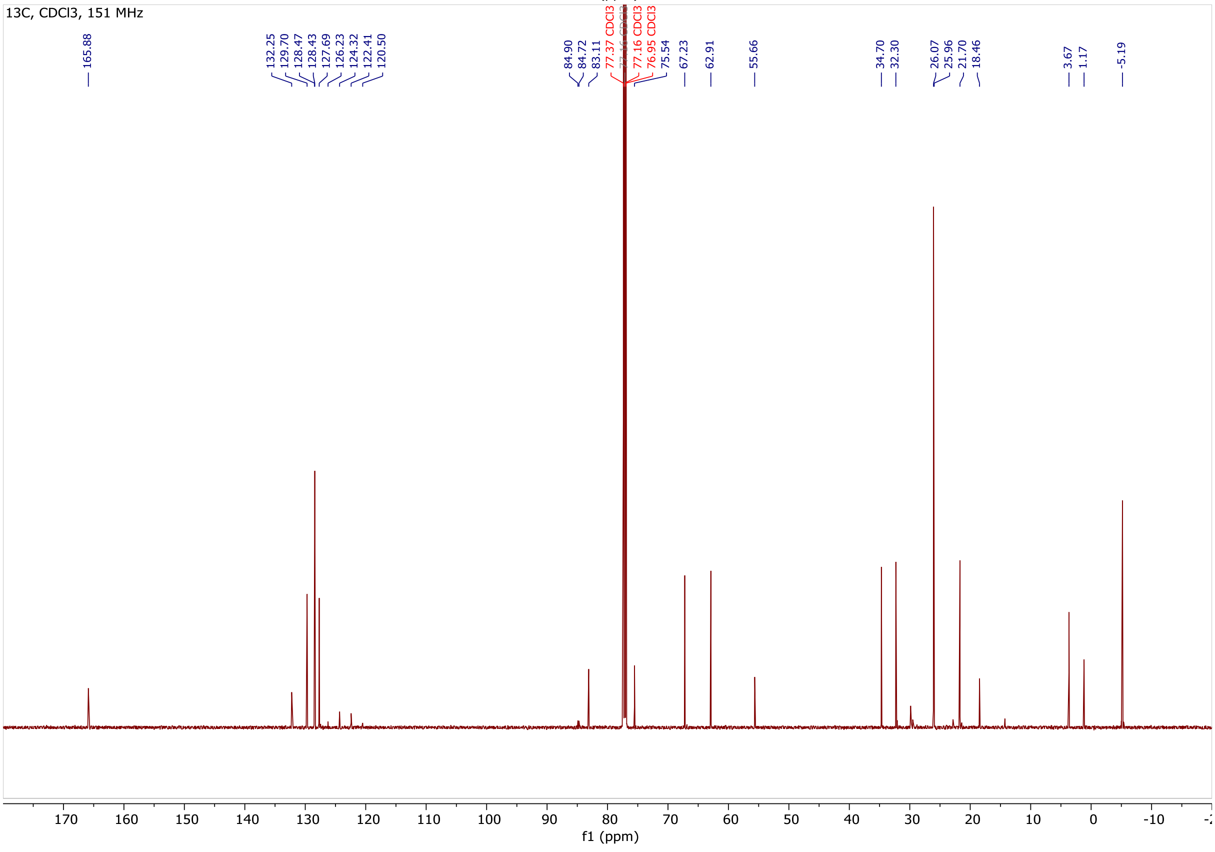

13C, CDCl3, 151 MHz
f1 (ppm)

Compound S3

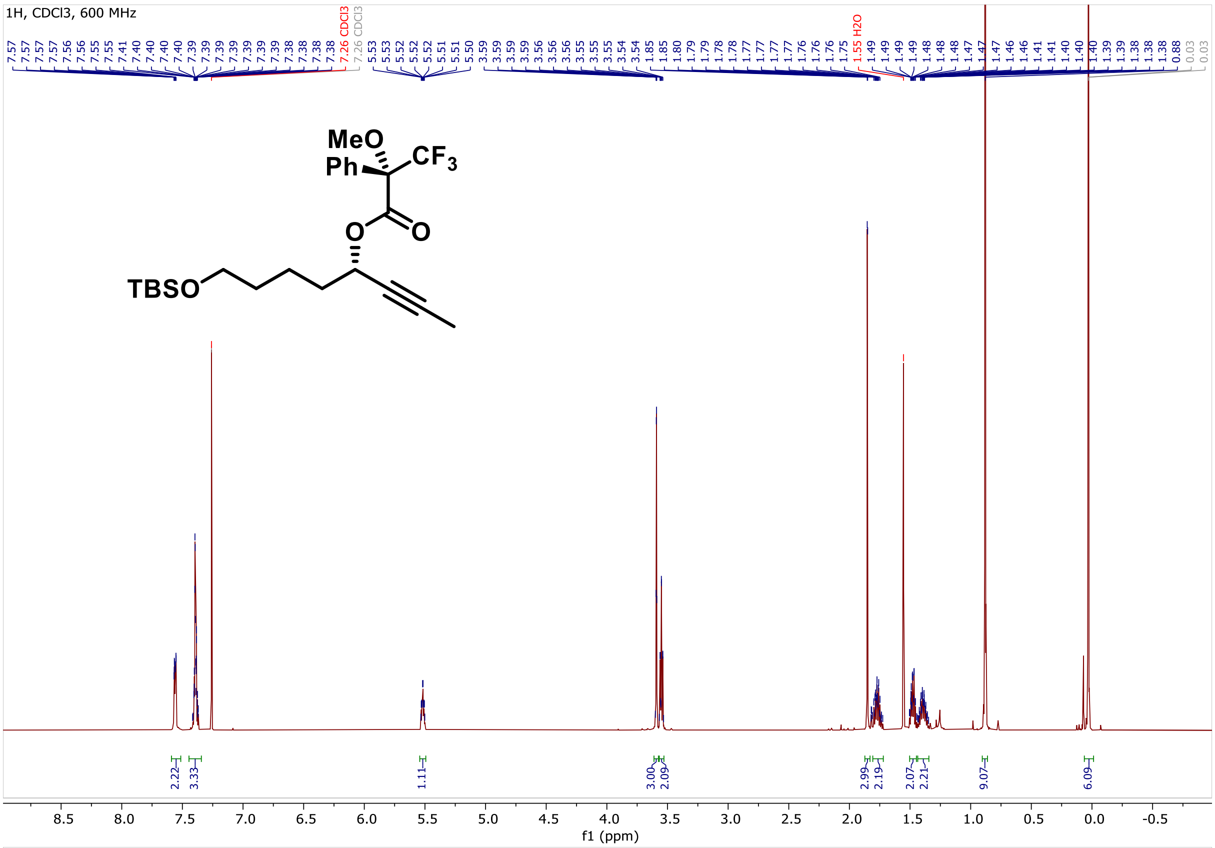
1H, CDCl3, 600 MHz
MeO
Ph
CF3
O
O
TBSO
f1 (ppm)

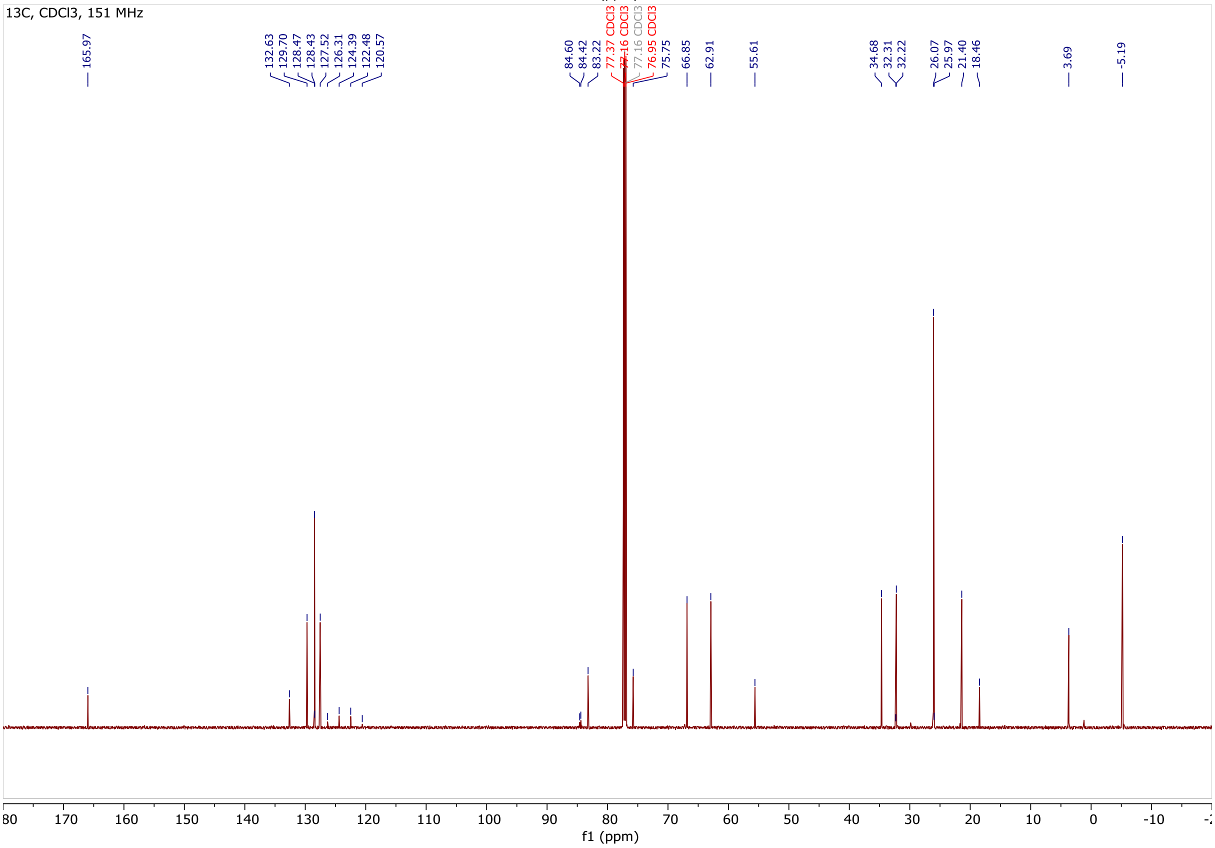
13C, CDCl3, 151 MHz
165.97
132.63
129.70
128.47
128.43
127.52
126.31
124.39
122.48
120.57
84.60
84.42
83.22
77.37 CDCl3
77.16 CDCl3
77.16 CDCl3
76.95 CDCl3
75.75
66.85
62.91
55.61
34.68
32.31
32.22
26.07
25.97
21.40
18.46
3.69
-5.19
f1 (ppm)

Compound S4

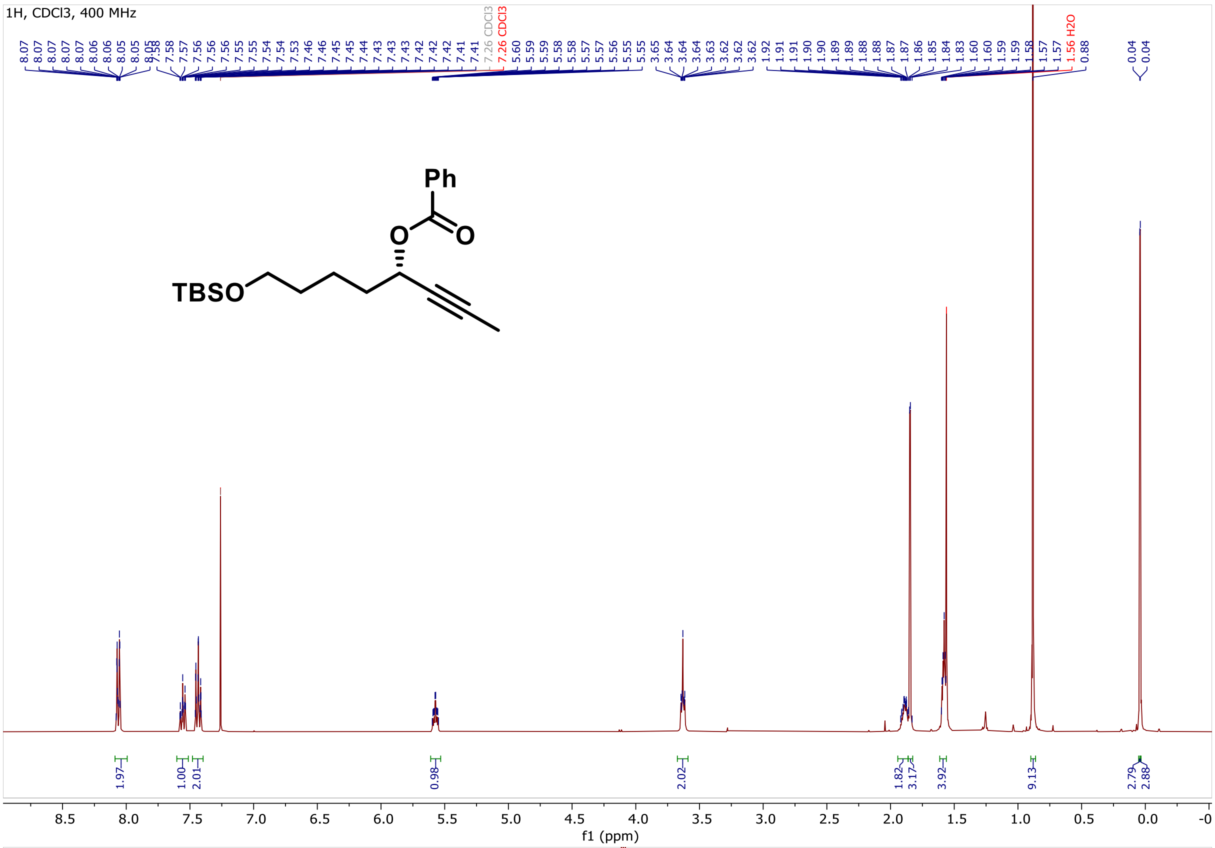
1H, CDCl3, 400 MHz
Ph
O
O
TBSO
1.97
1.00
2.01
0.98
2.02
1.82
3.17
3.92
9.13
2.79
2.88
f1 (ppm)

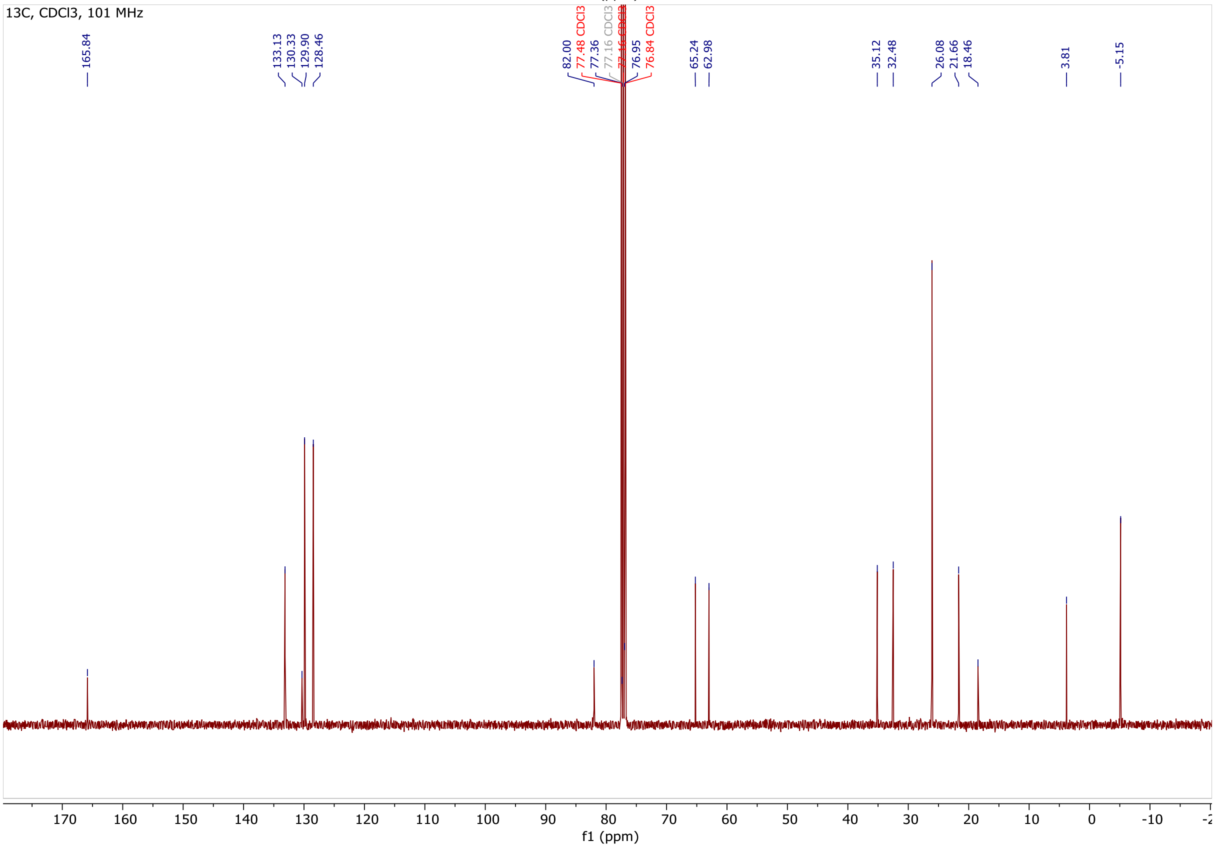
13C, CDCl3, 101 MHz
165.84
133.13
130.33
129.90
128.46
82.00
77.48 CDCl3
77.36
77.16 CDCl3
76.95
76.84 CDCl3
65.24
62.98
35.12
32.48
26.08
21.66
18.46
3.81
-5.15
f1 (ppm)

Compound 10

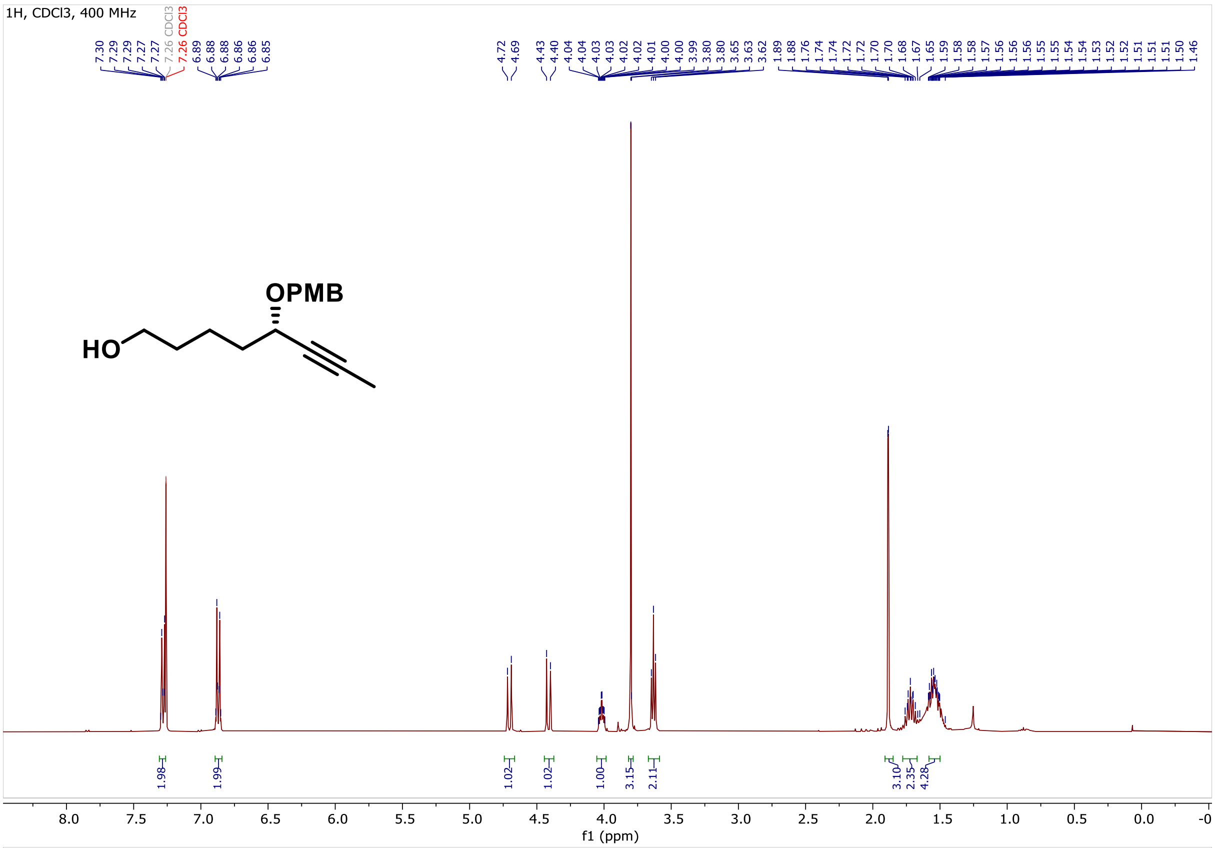

1H, CDCl3, 400 MHz
7.30
7.29
7.29
7.27
7.27
7.26 CDCl3
7.26 CDCl3
6.89
6.88
6.88
6.88
6.86
6.86
6.85
4.72
4.69
4.43
4.40
4.04
4.04
4.03
4.03
4.02
4.02
4.01
4.00
4.00
3.99
3.80
3.65
3.63
3.62
1.89
1.88
1.76
1.74
1.74
1.72
1.72
1.70
1.70
1.68
1.67
1.65
1.59
1.58
1.58
1.57
1.56
1.56
1.56
1.55
1.55
1.54
1.54
1.53
1.52
1.52
1.51
1.51
1.51
1.50
1.46
OPMB
HO
1.98
1.99
1.02
1.02
1.00
3.15
2.11
3.10
2.35
4.28
8.0
7.5
7.0
6.5
6.0
5.5
5.0
4.5
4.0
3.5
3.0
2.5
2.0
1.5
1.0
0.5
0.0
-0
f1 (ppm)

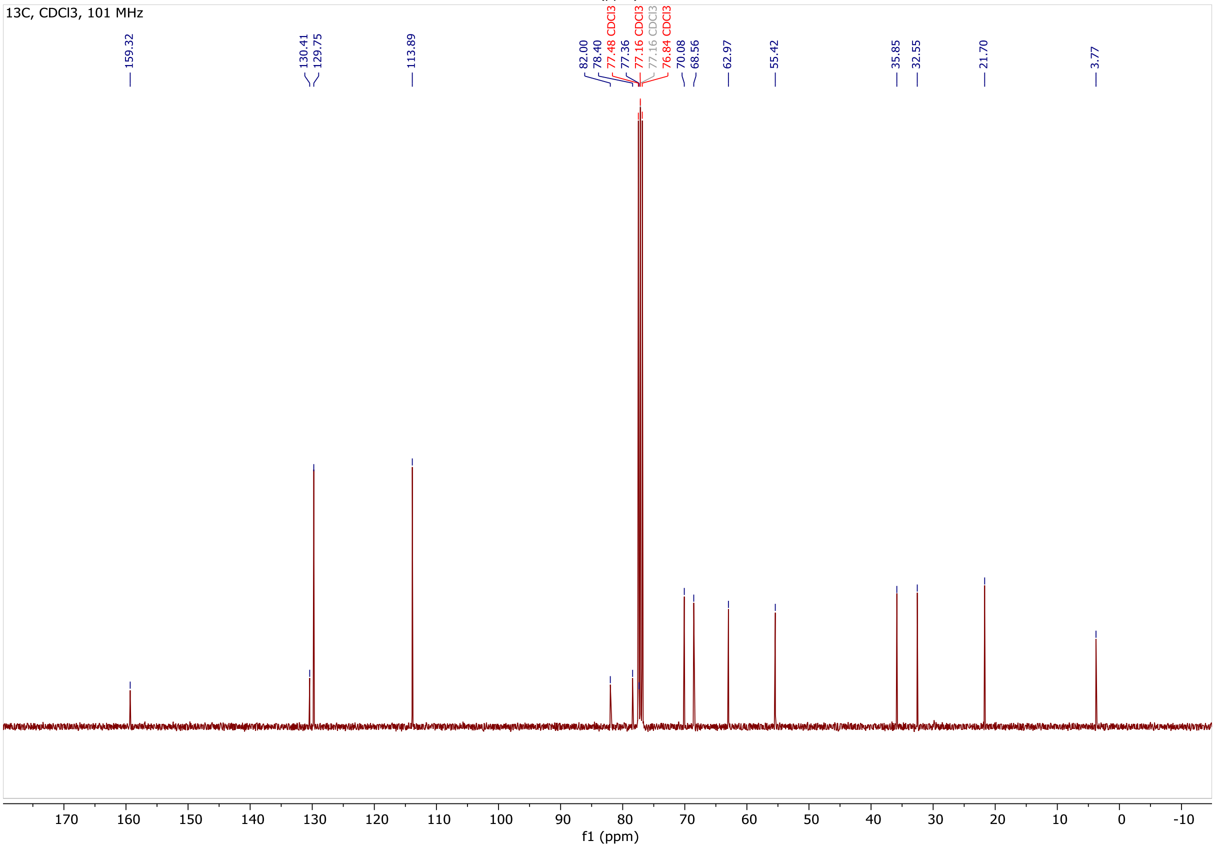

13C, CDCl3, 101 MHz
159.32
130.41
129.75
113.89
82.00
78.40
77.48 CDCl3
77.36
77.16 CDCl3
77.16 CDCl3
76.84 CDCl3
70.08
68.56
62.97
55.42
35.85
32.55
21.70
3.77
170
160
150
140
130
120
110
100
90
80
70
60
50
40
30
20
10
0
-10
f1 (ppm)

Compound 11

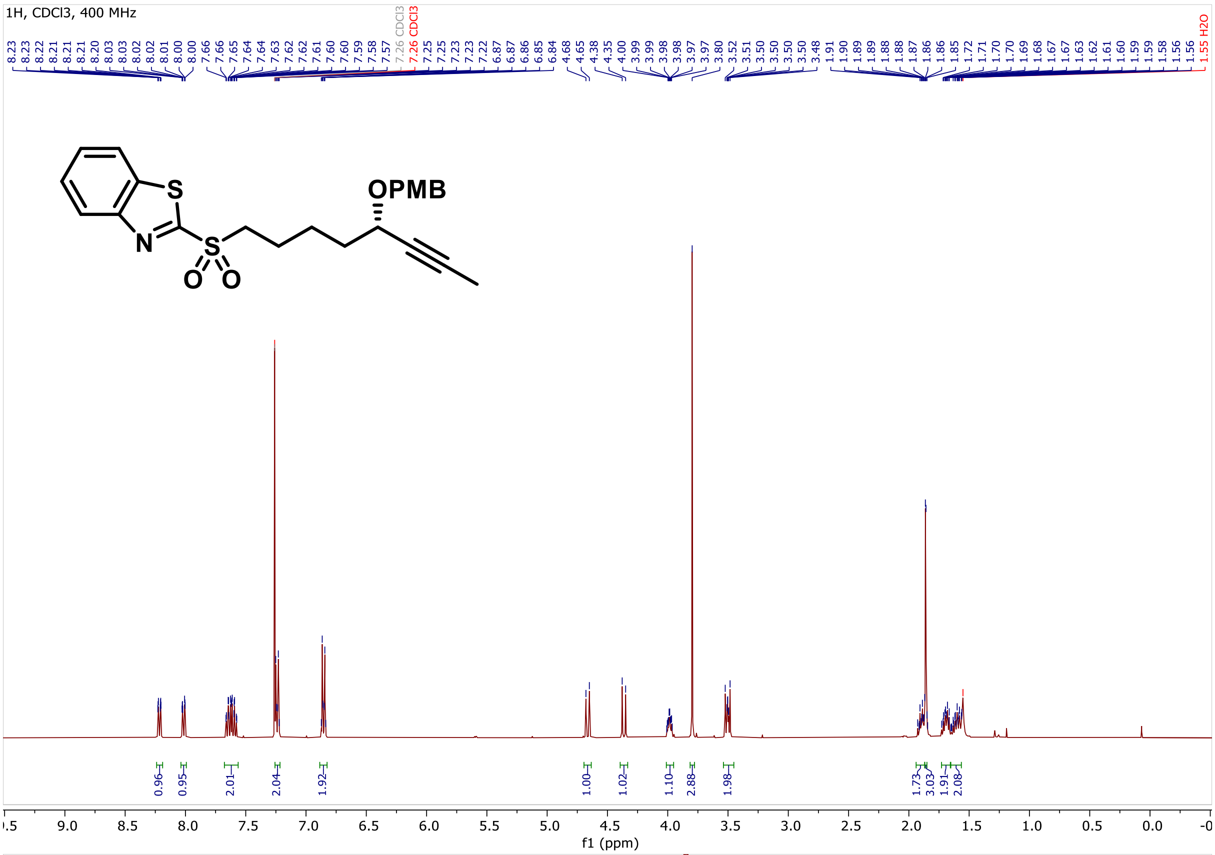
1H, CDCl3, 400 MHz
8.23 8.23 8.22 8.21 8.21 8.21 8.20 8.03 8.03 8.03 8.02 8.02 8.01 8.00 8.00 7.66 7.65 7.64 7.64 7.63 7.62 7.62 7.61 7.60 7.60 7.59 7.58 7.57 7.26 CDCl3 7.26 CDCl3 7.25 7.25 7.23 7.23 7.22 6.87 6.87 6.86 6.85 6.84 4.68 4.65 4.38 4.35 4.00 3.99 3.99 3.98 3.98 3.97 3.97 3.80 3.52 3.51 3.50 3.50 3.50 3.50 3.48 1.91 1.90 1.89 1.89 1.88 1.88 1.87 1.86 1.86 1.85 1.72 1.71 1.70 1.70 1.69 1.68 1.67 1.67 1.63 1.62 1.61 1.60 1.59 1.59 1.58 1.56 1.56 1.55 H2O
OPMB
S
N
S
O O
0.96 0.95 2.01 2.04 1.92 1.00 1.02 1.10 2.88 1.98 1.73 3.03 1.91 2.08
f1 (ppm)

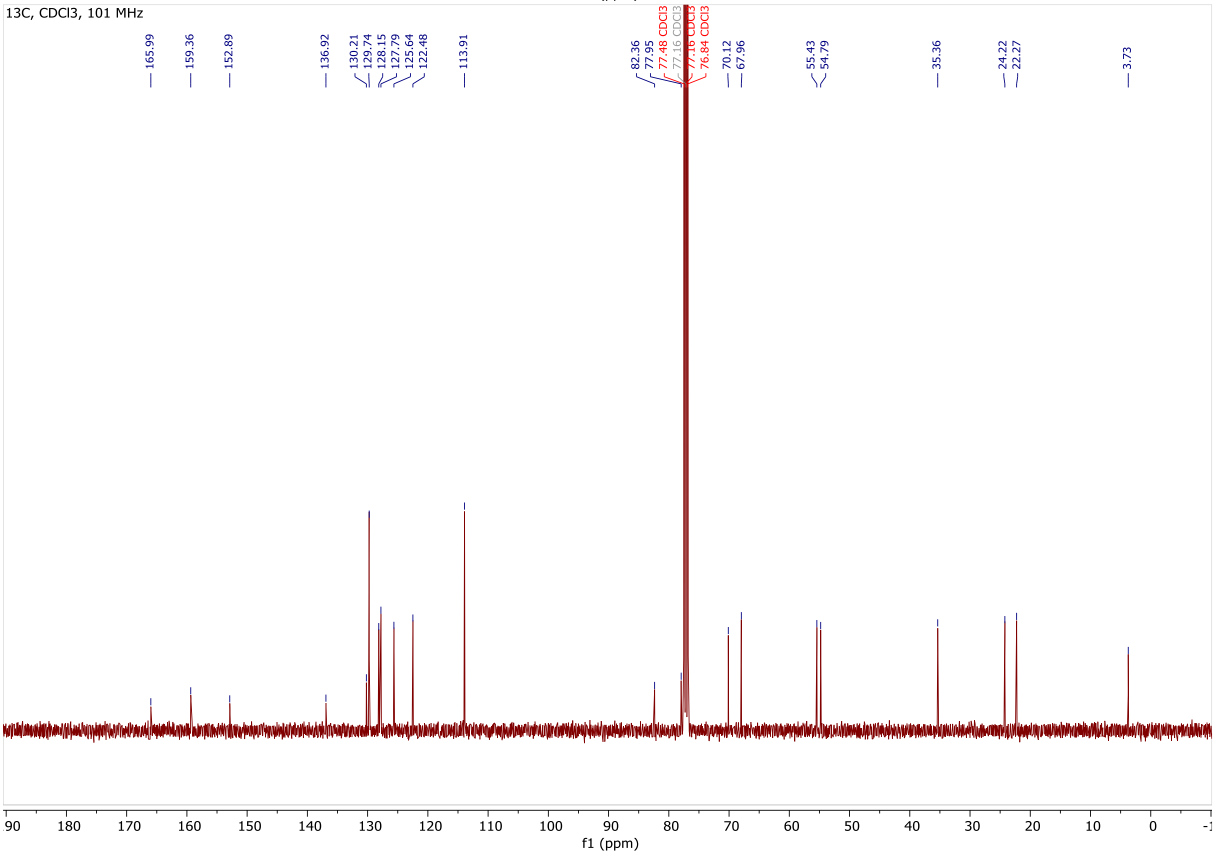
13C, CDCl3, 101 MHz
165.99 159.36 152.89 136.92 130.21 129.74 128.15 127.79 125.64 122.48 113.91 82.36 77.95 77.48 CDCl3 77.16 CDCl3 77.16 CDCl3 76.84 CDCl3 70.12 67.96 55.43 54.79 35.36 24.22 22.27 3.73
f1 (ppm)

Compound S5

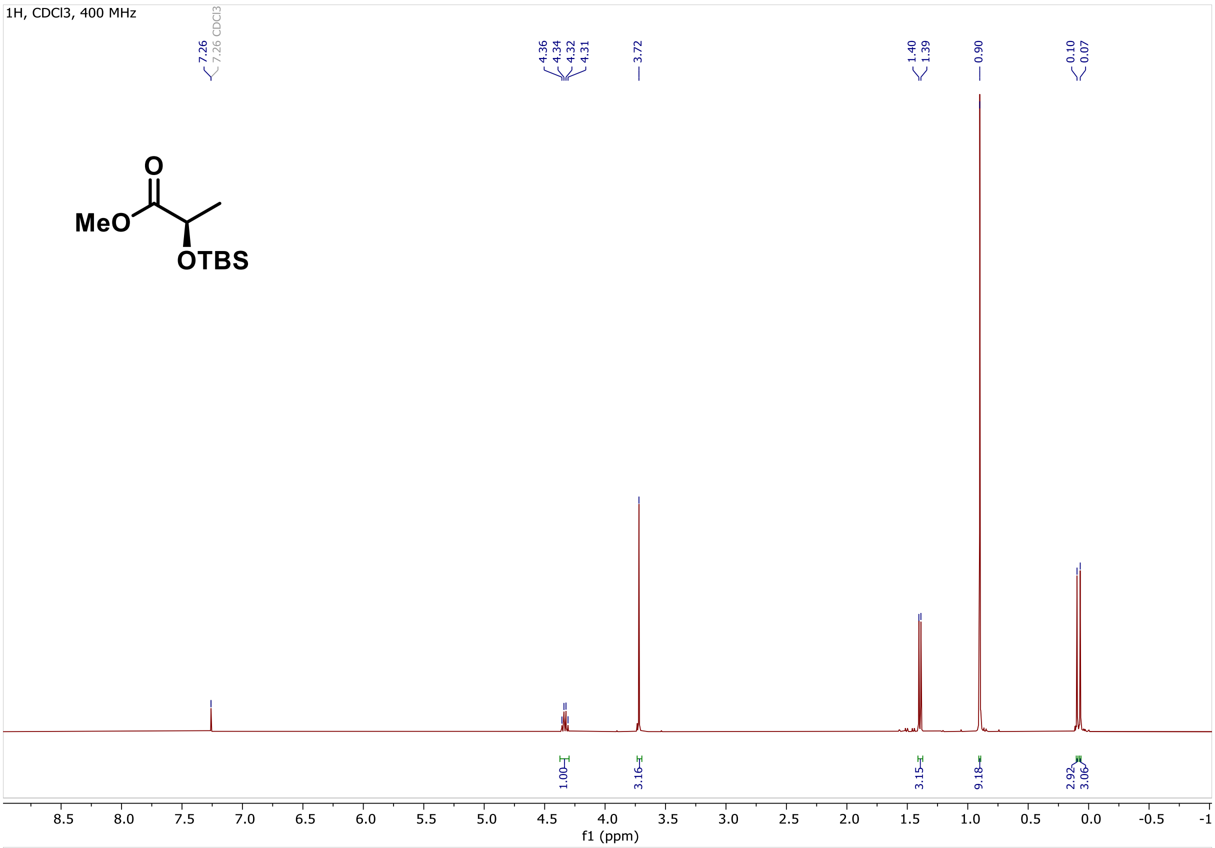

1H, CDCl3, 400 MHz
7.26
7.26 CDCl3
4.36
4.34
4.32
4.31
3.72
1.40
1.39
0.90
0.10
0.07
MeO
O
OTBS
1.00
3.16
3.15
9.18
2.92
3.06
8.5
8.0
7.5
7.0
6.5
6.0
5.5
5.0
4.5
4.0
3.5
3.0
2.5
2.0
1.5
1.0
0.5
0.0
-0.5
-1
f1 (ppm)

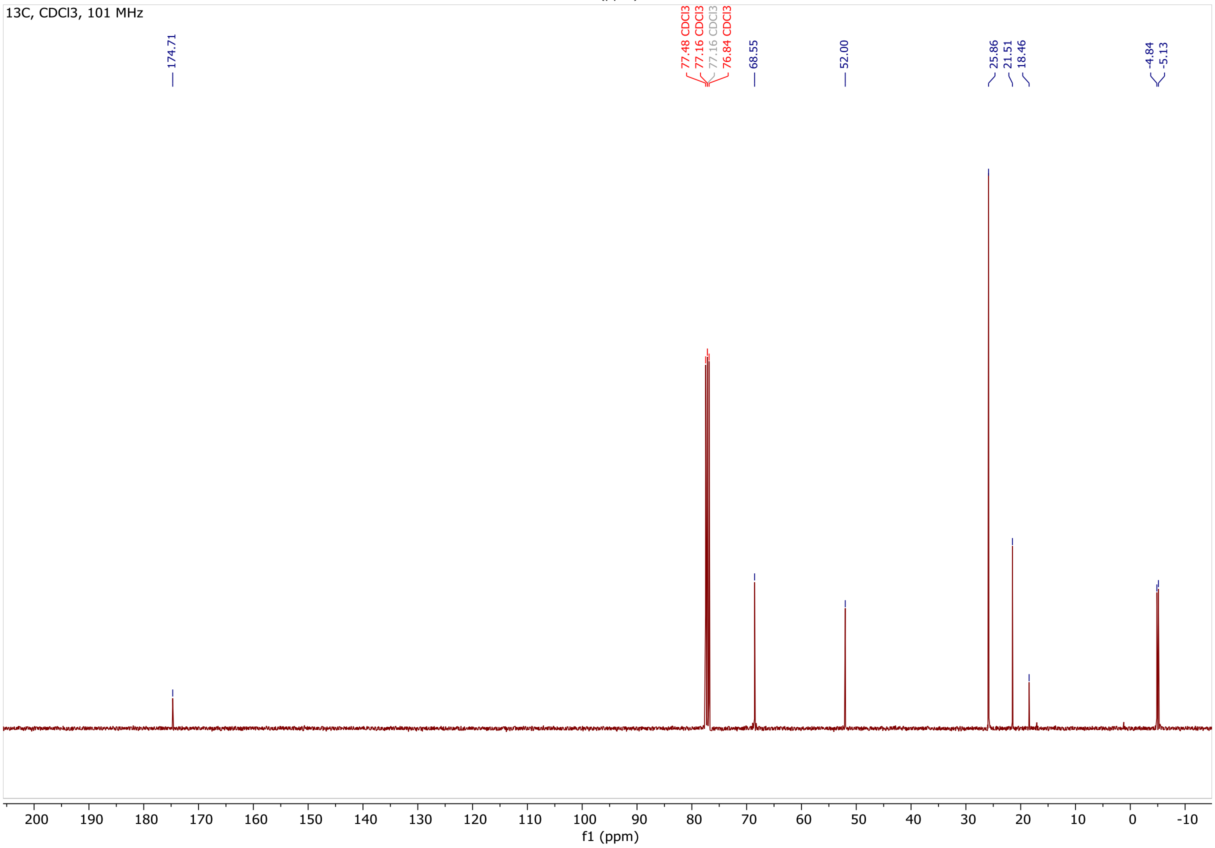

13C, CDCl3, 101 MHz
174.71
77.48 CDCl3
77.16 CDCl3
77.16 CDCl3
76.84 CDCl3
68.55
52.00
25.86
21.51
18.46
-4.84
-5.13
200
190
180
170
160
150
140
130
120
110
100
90
80
70
60
50
40
30
20
10
0
-10
f1 (ppm)

Compound 14

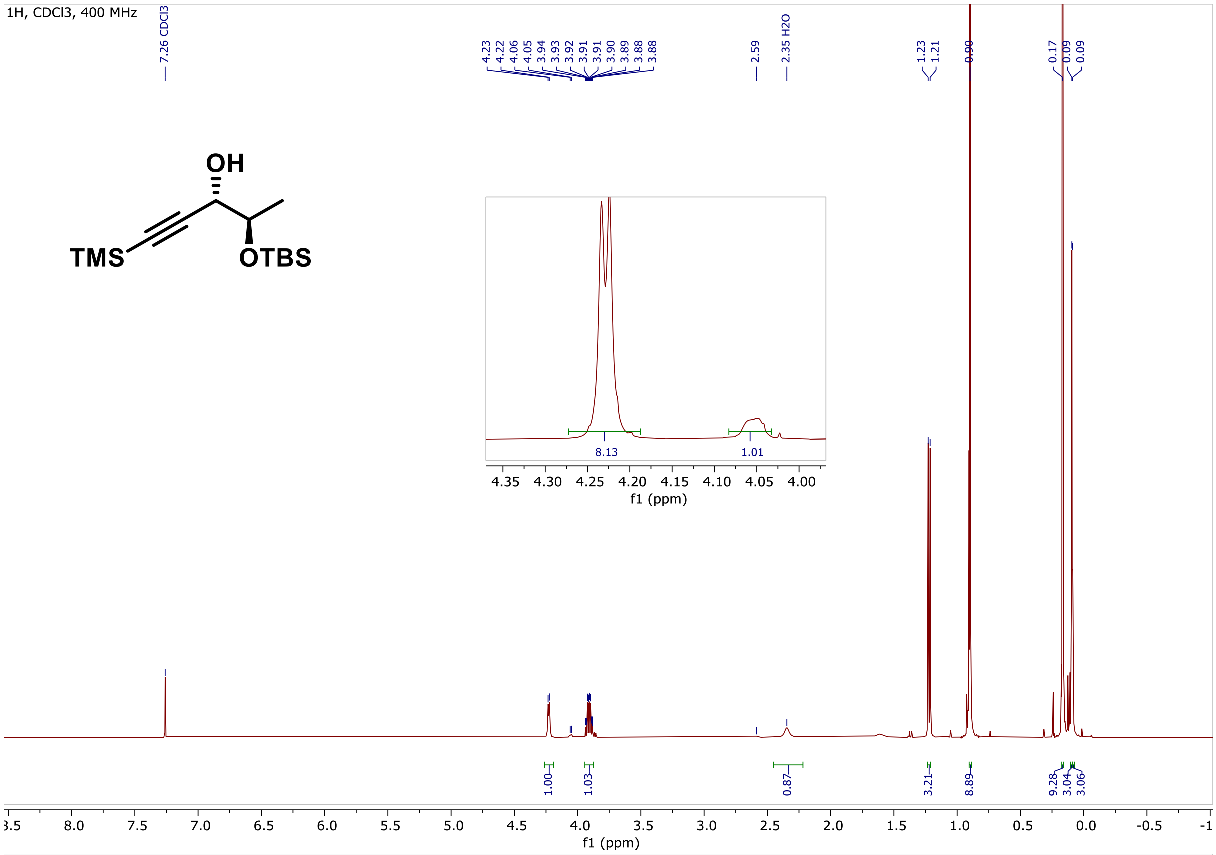
1H, CDCl3, 400 MHz
7.26 CDCl3
4.23
4.22
4.06
4.05
3.94
3.93
3.92
3.91
3.91
3.90
3.89
3.88
3.88
2.59
2.35 H2O
1.23
1.21
0.90
0.17
0.09
0.09
OH
TMS
OTBS
8.13
1.01
f1 (ppm)
1.00
1.03
0.87
3.21
8.89
9.28
3.04
3.06
f1 (ppm)

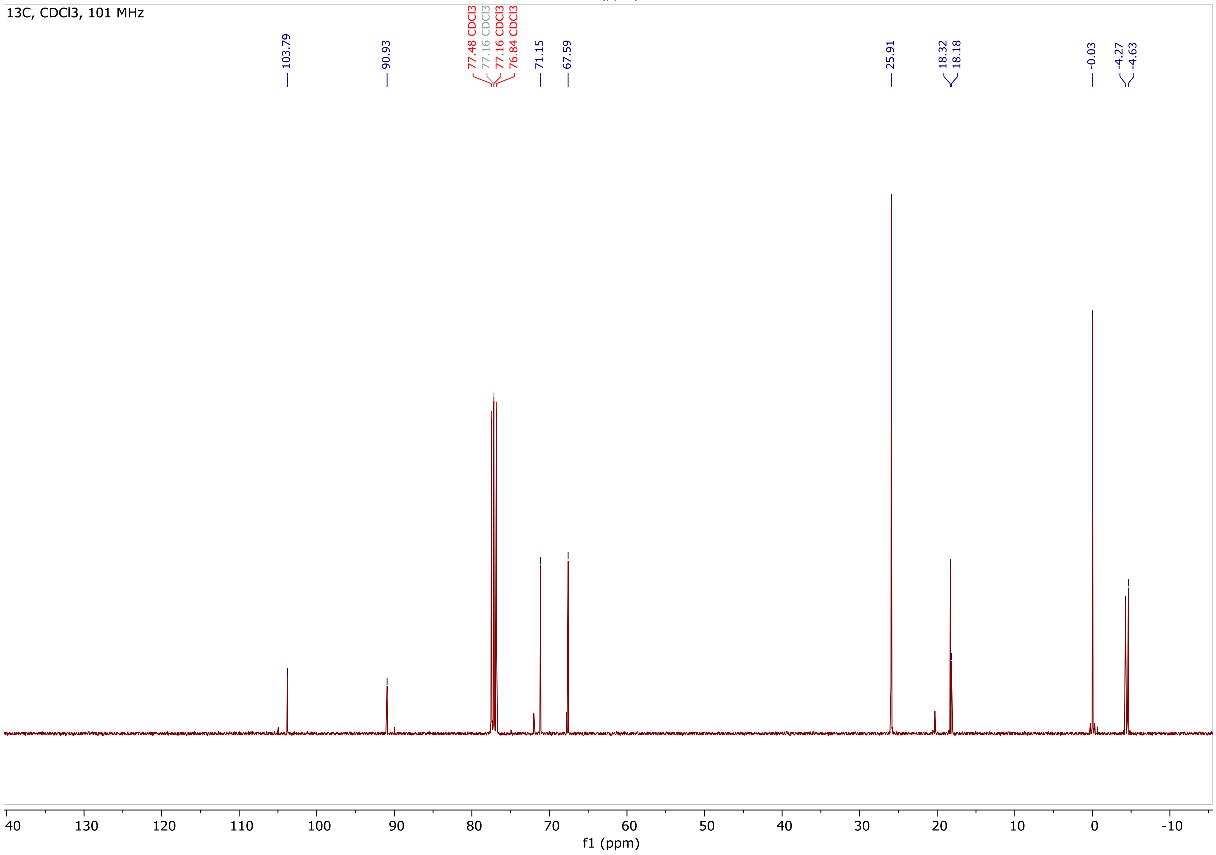
13C, CDCl3, 101 MHz
103.79
90.93
77.48 CDCl3
77.16 CDCl3
77.16 CDCl3
76.84 CDCl3
71.15
67.59
25.91
18.32
18.18
-0.03
-4.27
-4.63
f1 (ppm)

Compound 15

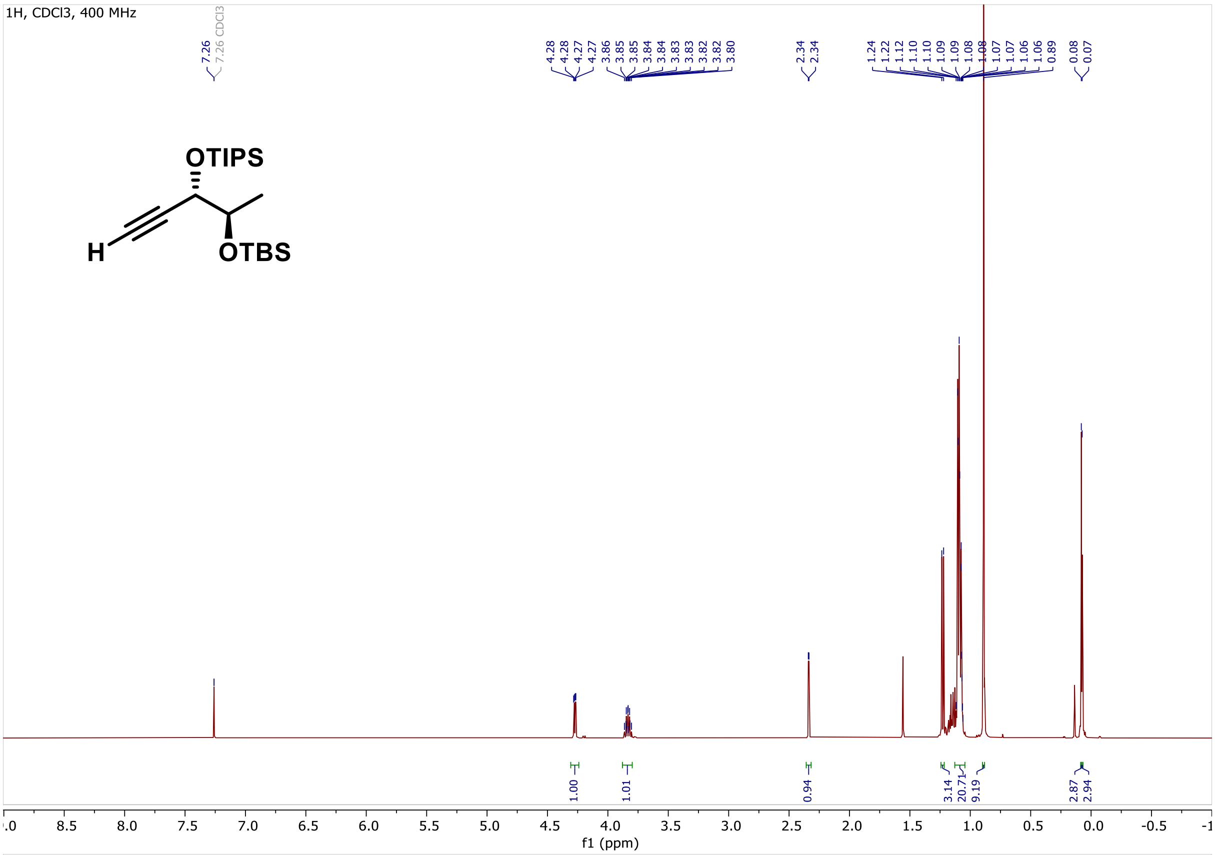
1H, CDCl3, 400 MHz
7.26
7.26 CDCl3
4.28 4.28 4.27 4.27
3.86 3.85 3.85 3.84 3.84 3.83 3.83 3.82 3.82 3.80
2.34 2.34
1.24 1.22 1.12 1.10 1.09 1.09 1.08 1.08 1.07 1.07 1.06 1.06 0.89
0.08 0.07
OTIPS
H
OTBS
1.00
1.01
0.94
3.14
20.71
9.19
2.87
2.94
f1 (ppm)

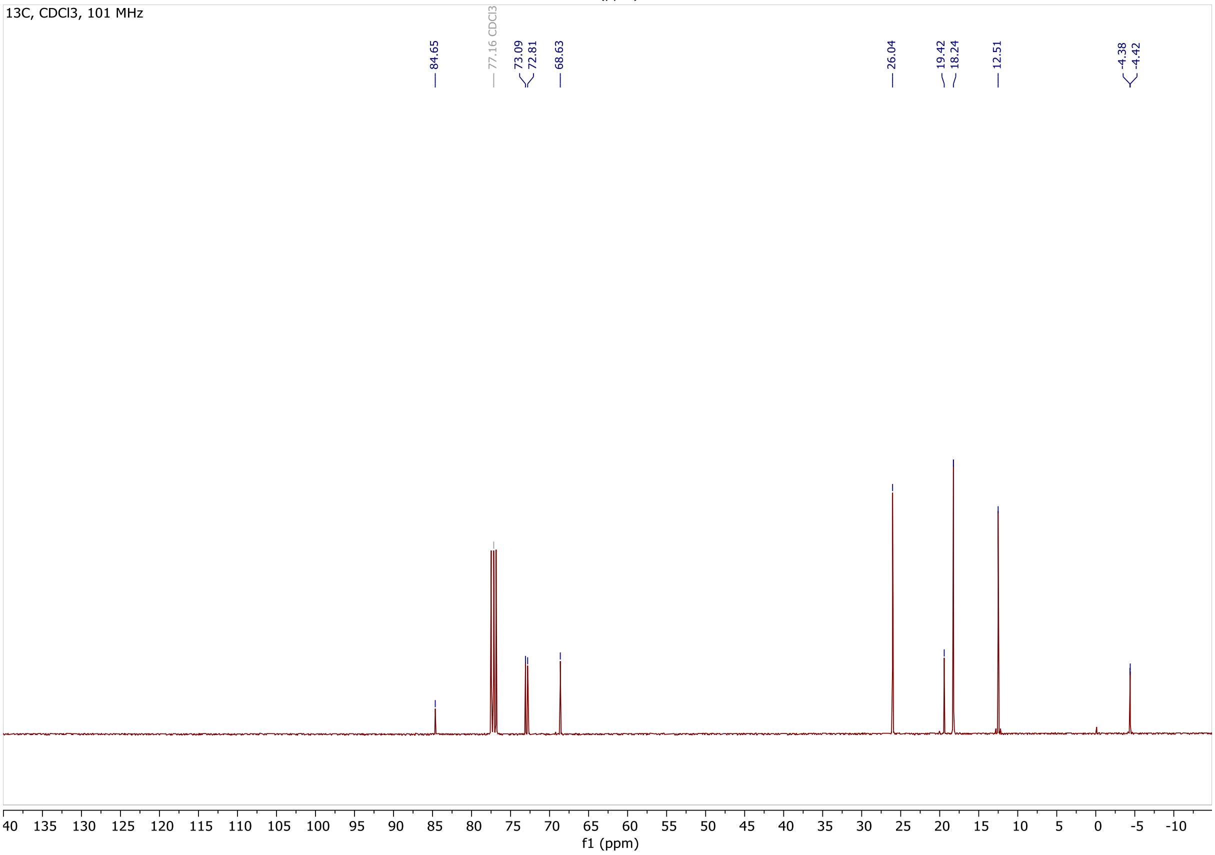
13C, CDCl3, 101 MHz
84.65
77.16 CDCl3
73.09
72.81
68.63
26.04
19.42
18.24
12.51
-4.38
-4.42
f1 (ppm)

Compound 16

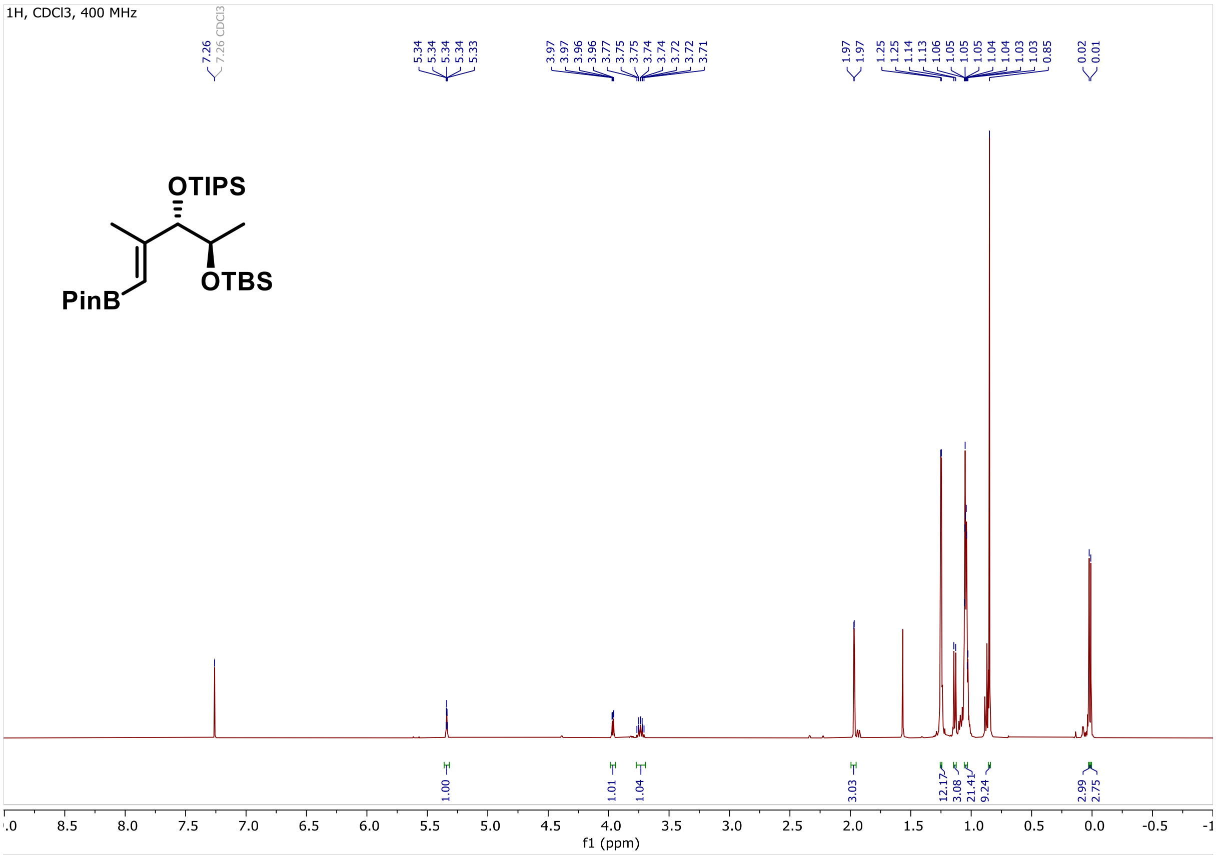
1H, CDCl3, 400 MHz
OTIPS
OTBS
PinB
f1 (ppm)

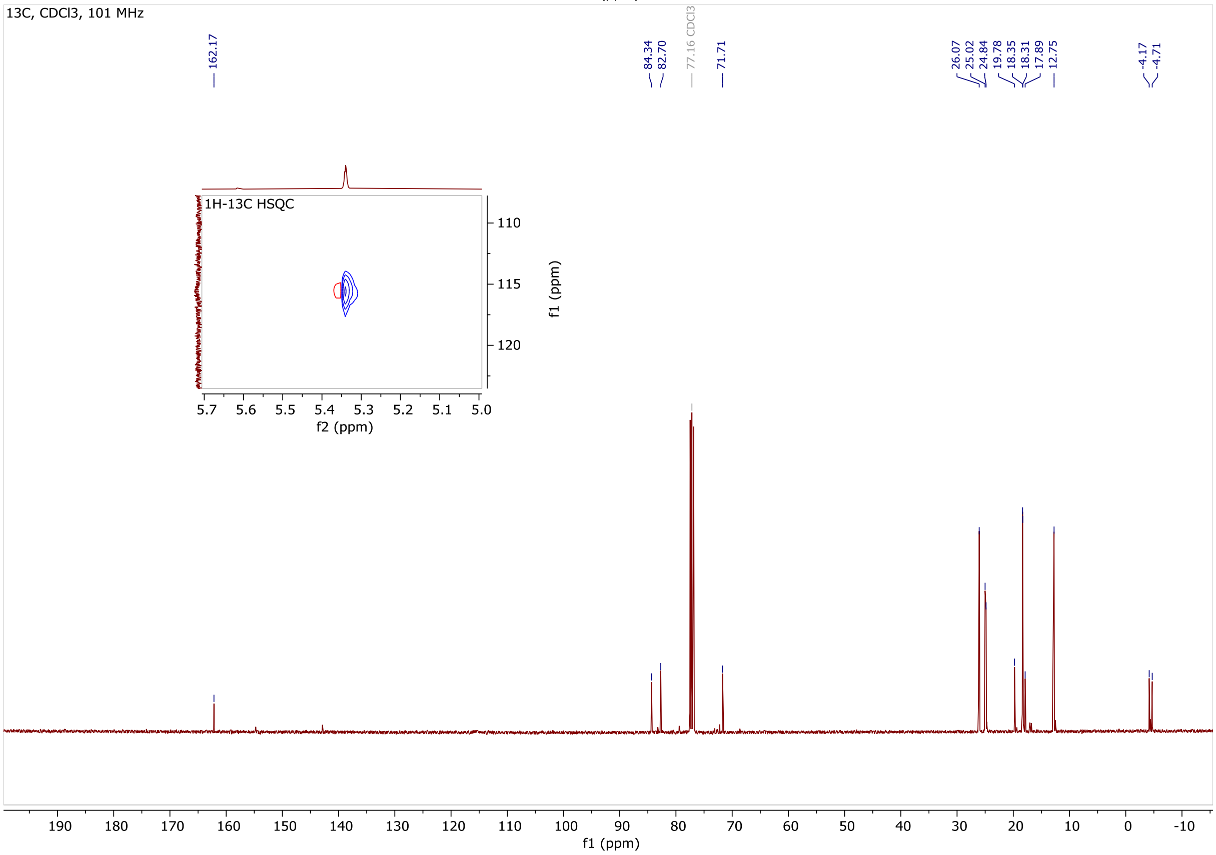
13C, CDCl3, 101 MHz
1H-13C HSQC
f2 (ppm)
f1 (ppm)

Compund S6

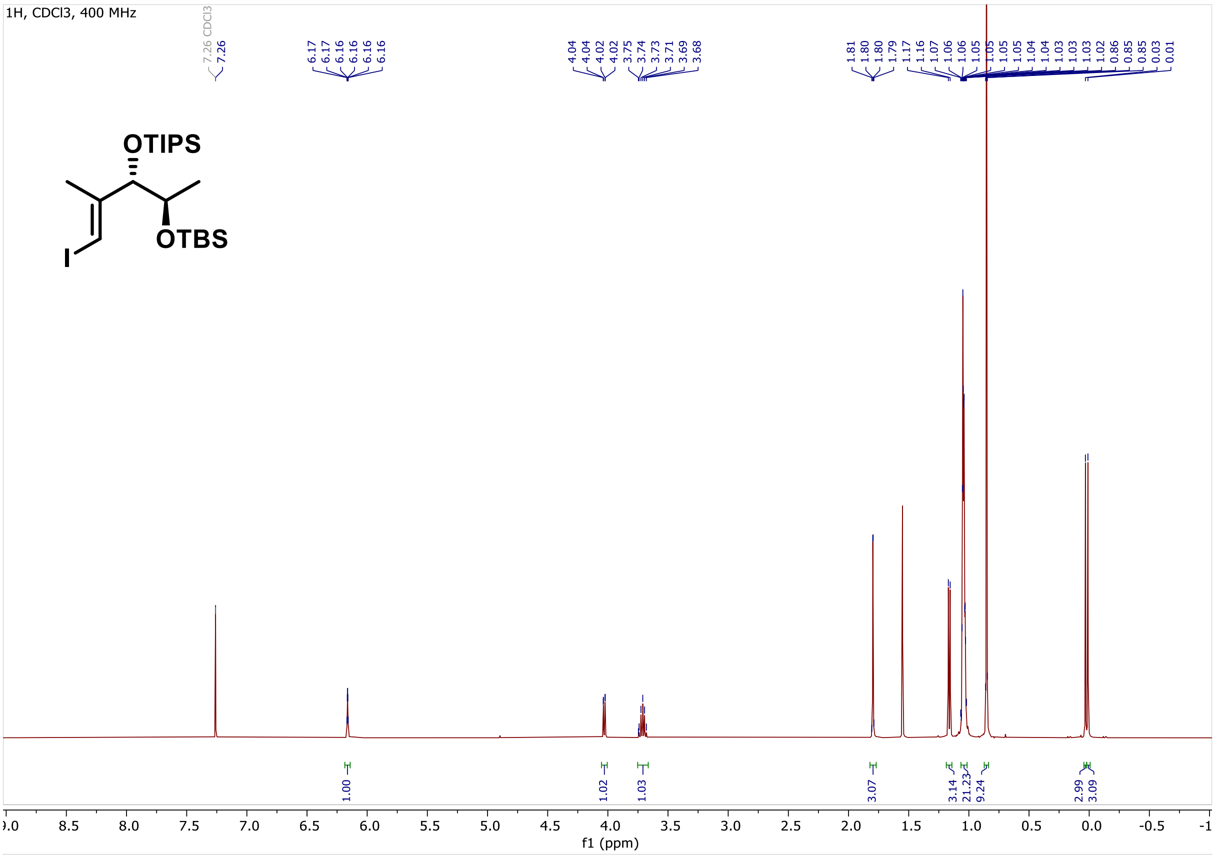
1H, CDCl3, 400 MHz
OTIPS
OTBS
I
7.26 CDCl3
7.26
6.17 6.17 6.16 6.16 6.16 6.16
4.04 4.04 4.02 4.02 3.75 3.74 3.73 3.71 3.69 3.68
1.81 1.80 1.80 1.79 1.17 1.16 1.07 1.06 1.06 1.05 1.05 1.05 1.05 1.04 1.04 1.03 1.03 1.03 1.02 0.86 0.85 0.85 0.03 0.01
1.00
1.02
1.03
3.07
3.14
21.23
9.24
2.99
3.09
f1 (ppm)

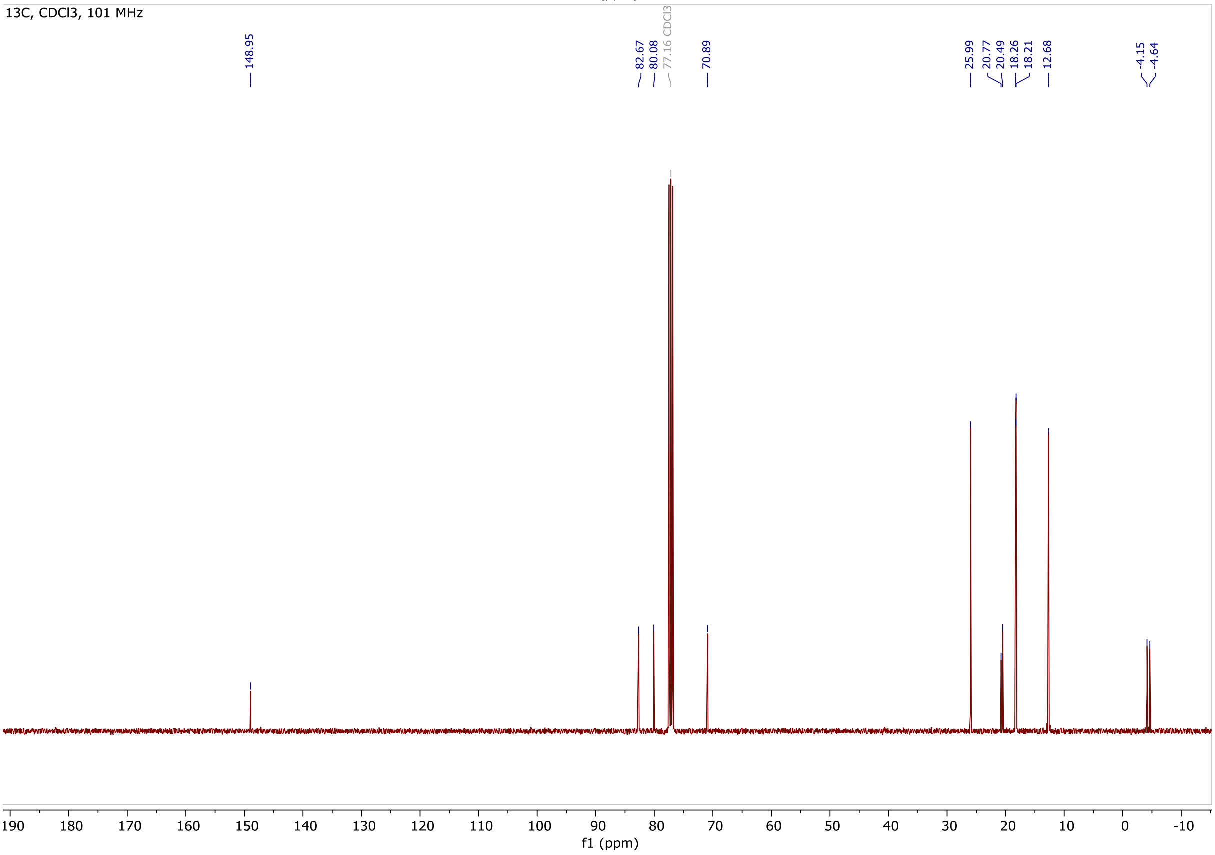
13C, CDCl3, 101 MHz
148.95
82.67
80.08
77.16 CDCl3
70.89
25.99
20.77
20.49
18.26
18.21
12.68
-4.15
-4.64
f1 (ppm)

Compound 17

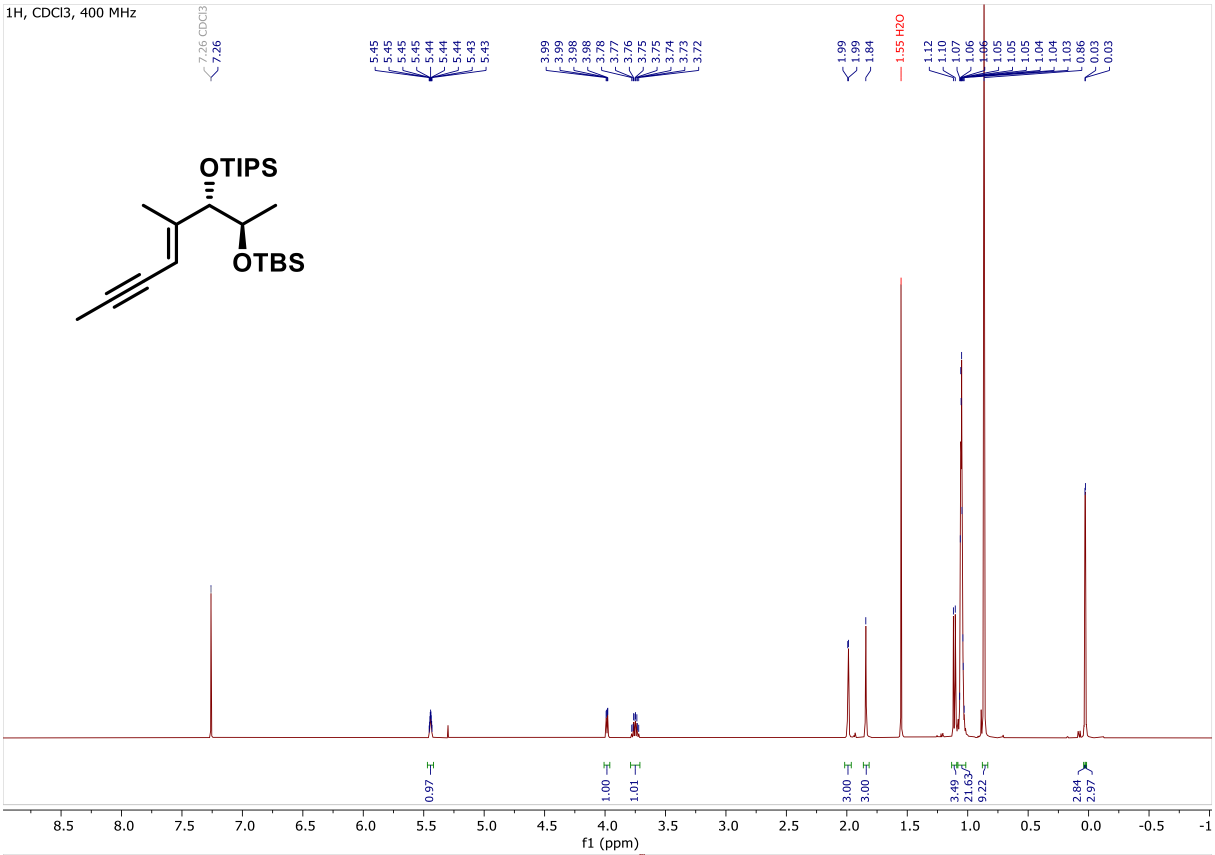

1H, CDCl3, 400 MHz
OTIPS
OTBS
7.26 CDCl3
7.26
5.45
5.45
5.45
5.45
5.44
5.44
5.44
5.43
5.43
3.99
3.99
3.98
3.98
3.78
3.77
3.76
3.75
3.75
3.74
3.73
3.72
1.99
1.99
1.84
1.55 H2O
1.12
1.10
1.07
1.06
1.06
1.05
1.05
1.05
1.04
1.04
1.03
0.86
0.03
0.03
0.97
1.00
1.01
3.00
3.00
3.49
21.63
9.22
2.84
2.97
f1 (ppm)

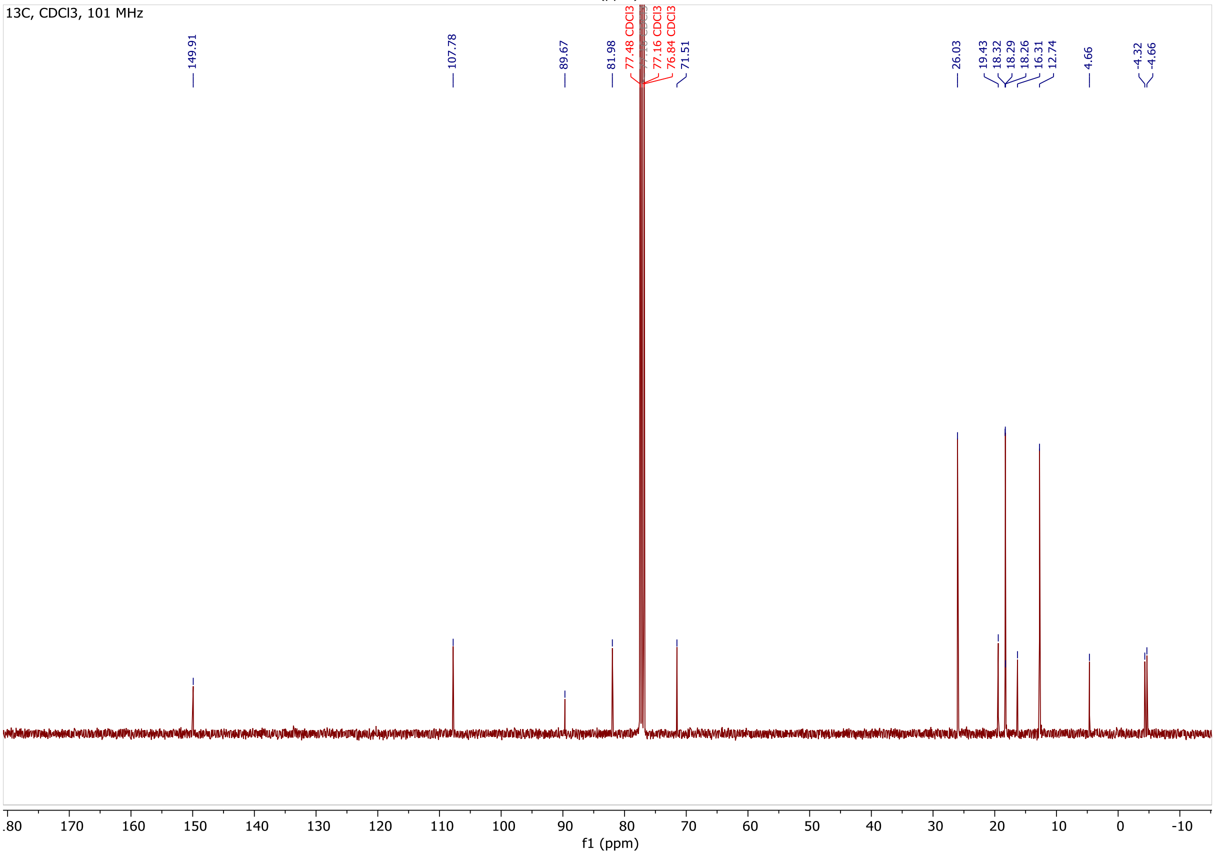

13C, CDCl3, 101 MHz
149.91
107.78
89.67
81.98
77.48 CDCl3
77.16 CDCl3
76.84 CDCl3
71.51
26.03
19.43
18.32
18.29
18.26
16.31
12.74
4.66
-4.32
-4.66
f1 (ppm)

Compound 18

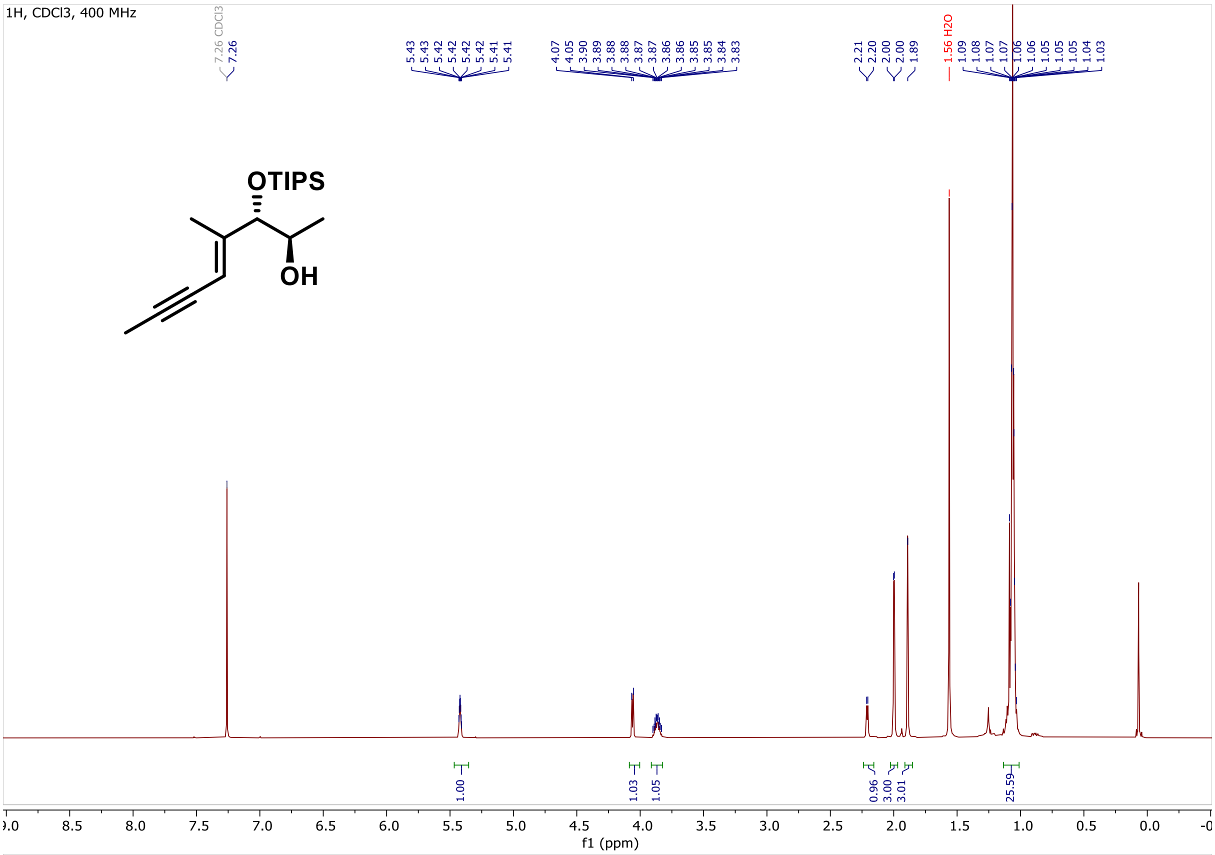
1H, CDCl3, 400 MHz
OTIPS
OH
f1 (ppm)

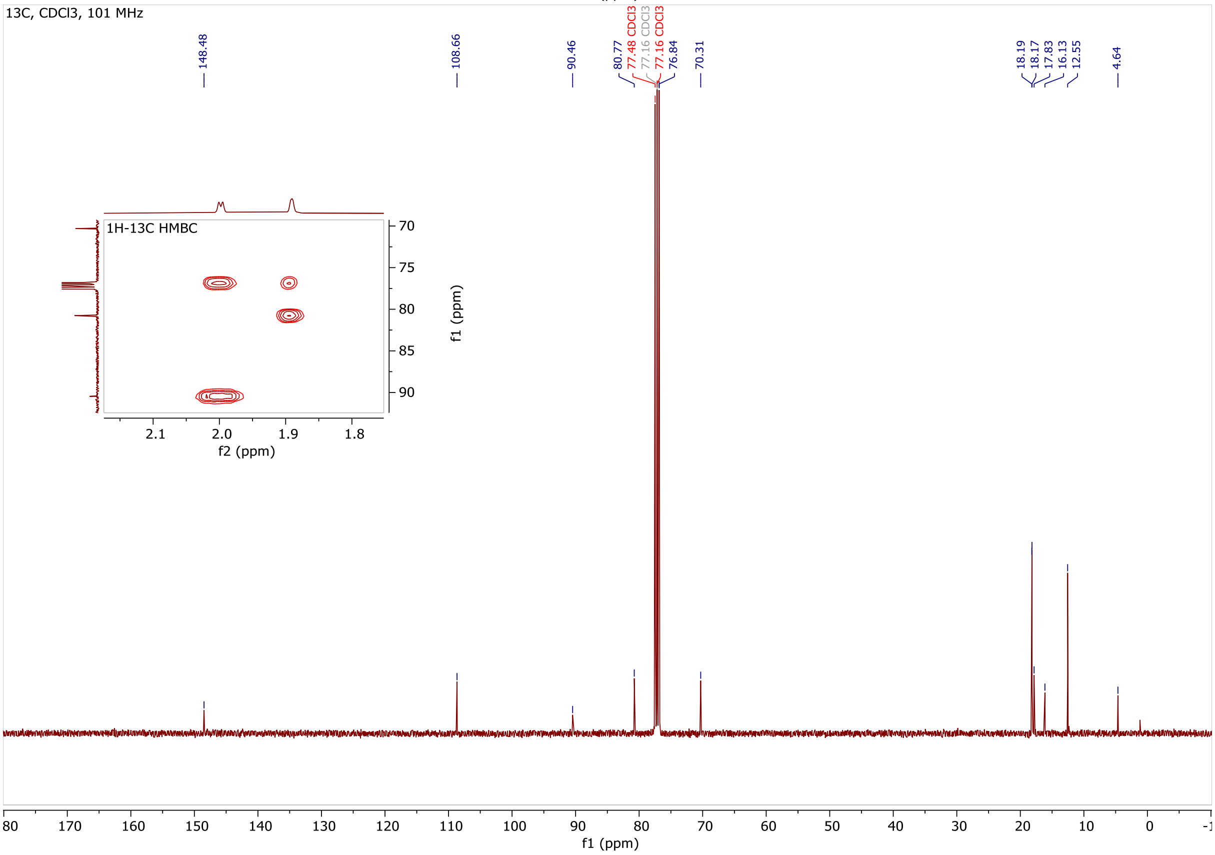
13C, CDCl3, 101 MHz
1H-13C HMBC
f2 (ppm)
f1 (ppm)

Compound 19 (*E*:*Z* ≈ 7:1)

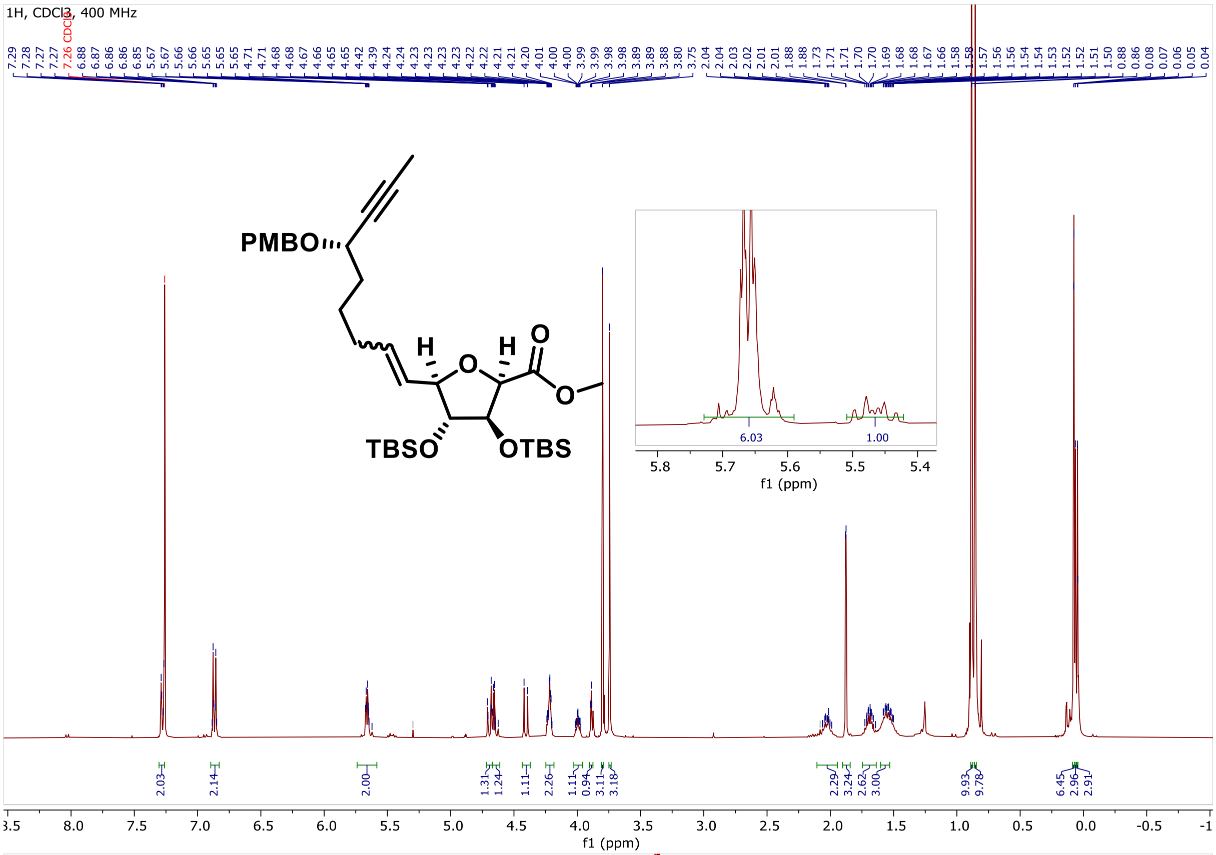

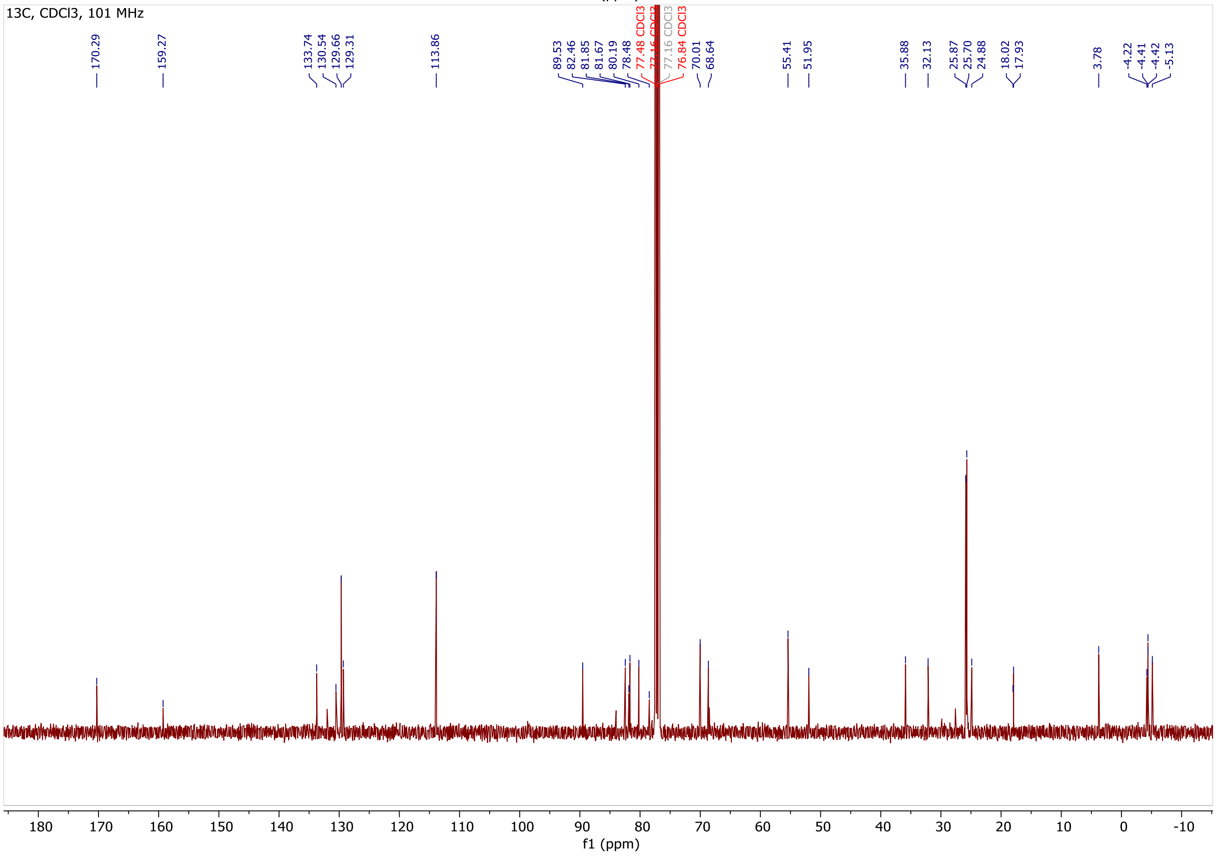

Compound 11-*epi*-19 (*E*:*Z* ≈ 8:1)

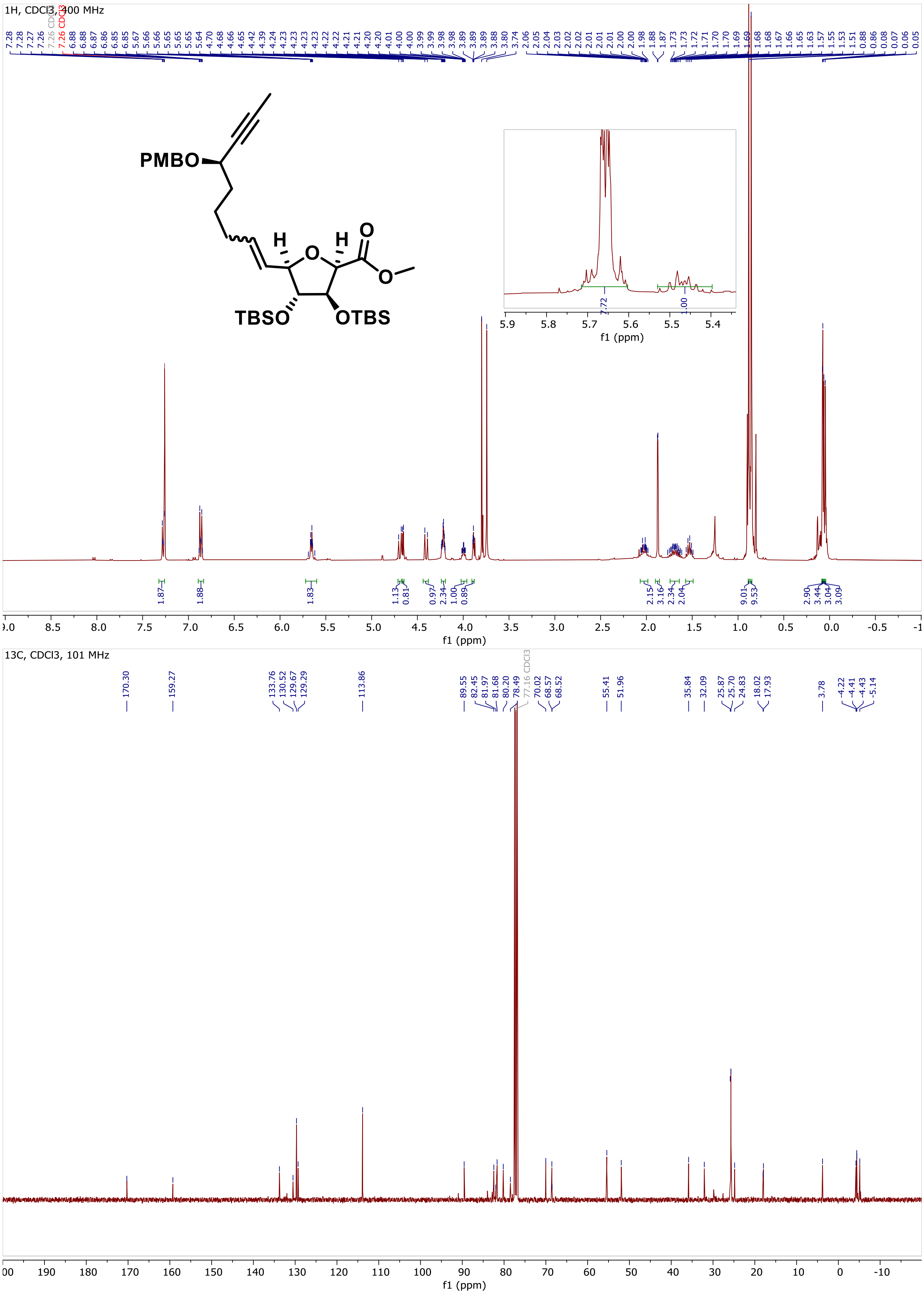

Compound S7 (*E*:*Z* ≈ 6:1)

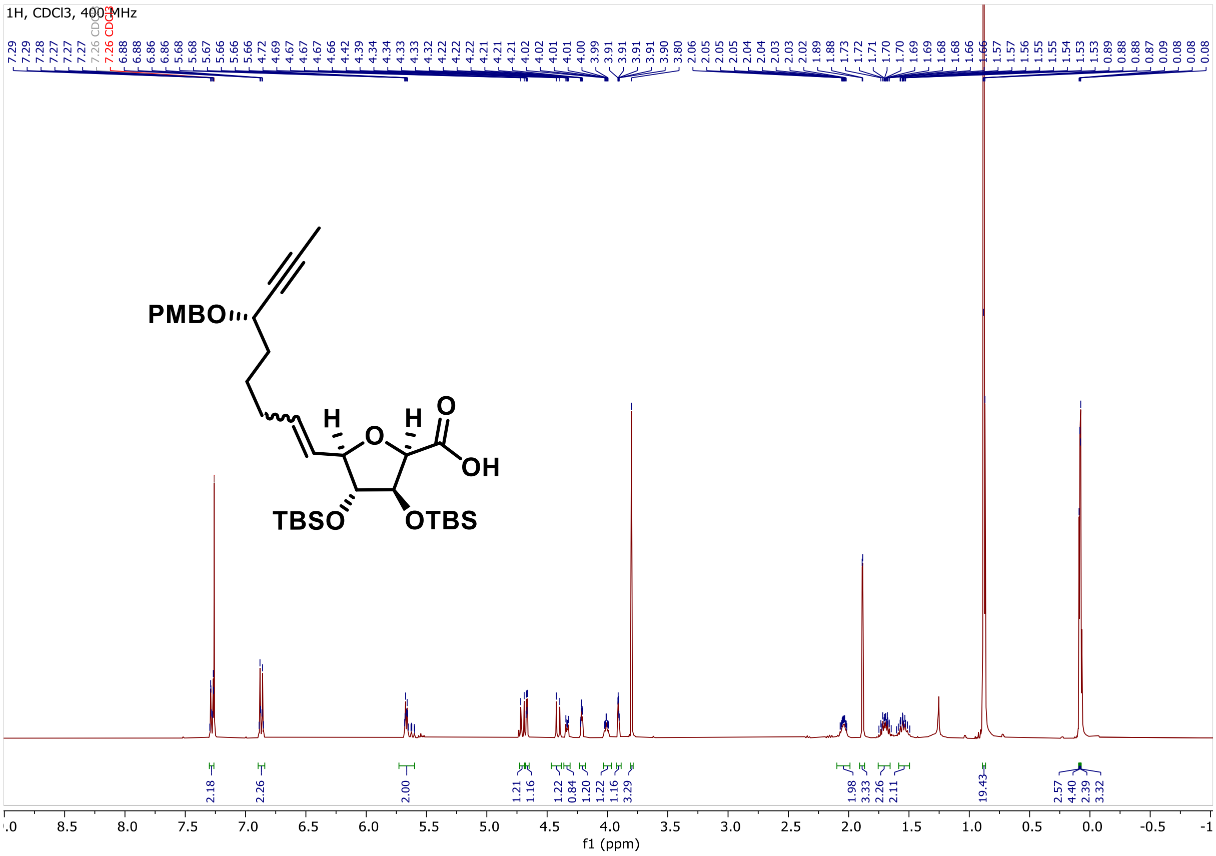

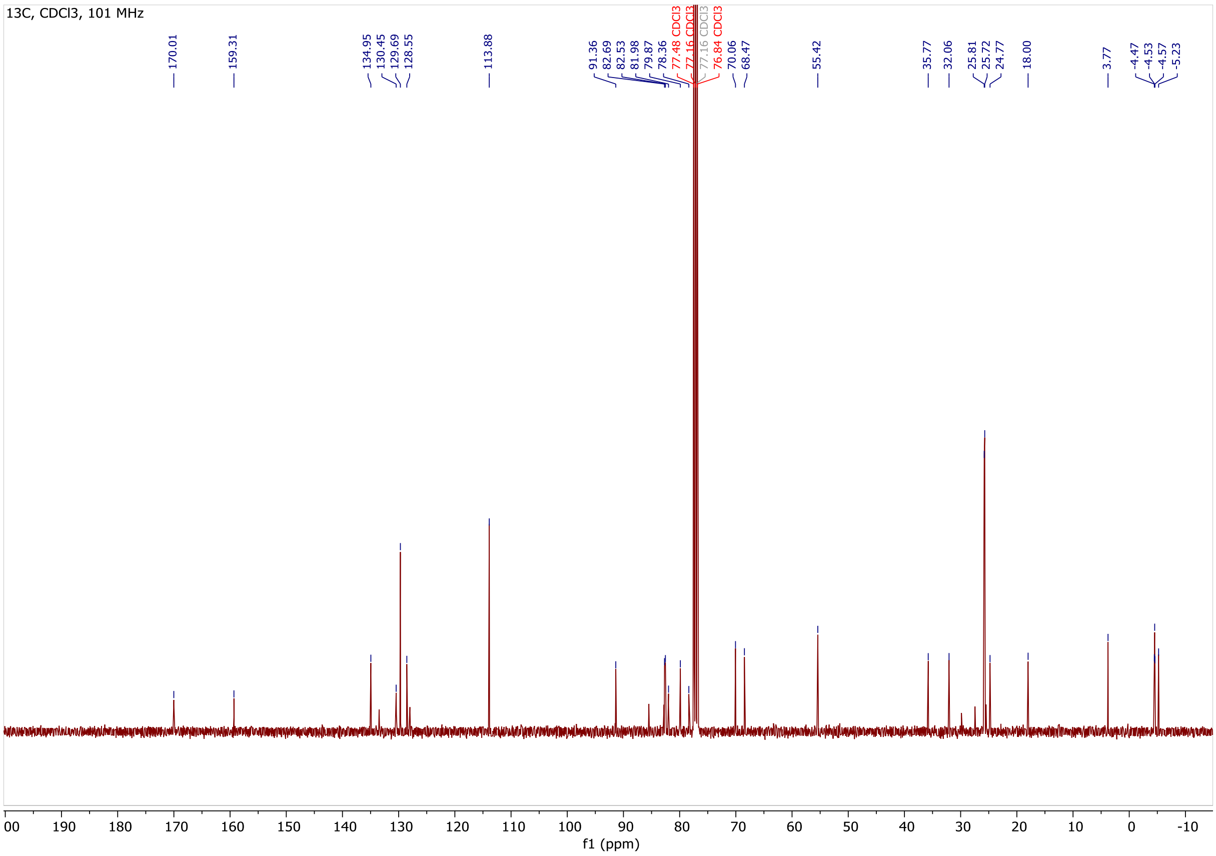

Compound 11-*epi*-S7 (*E*:*Z* ≈ 8:1)

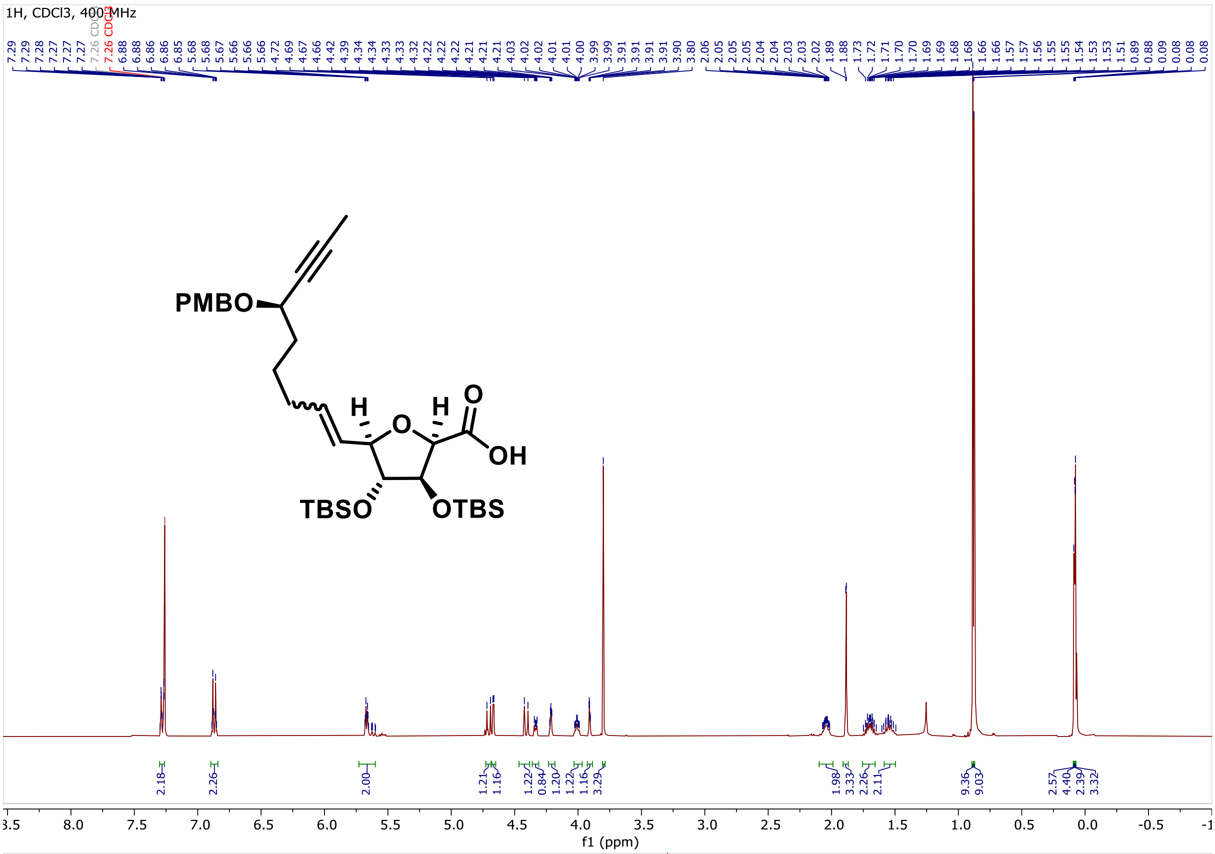

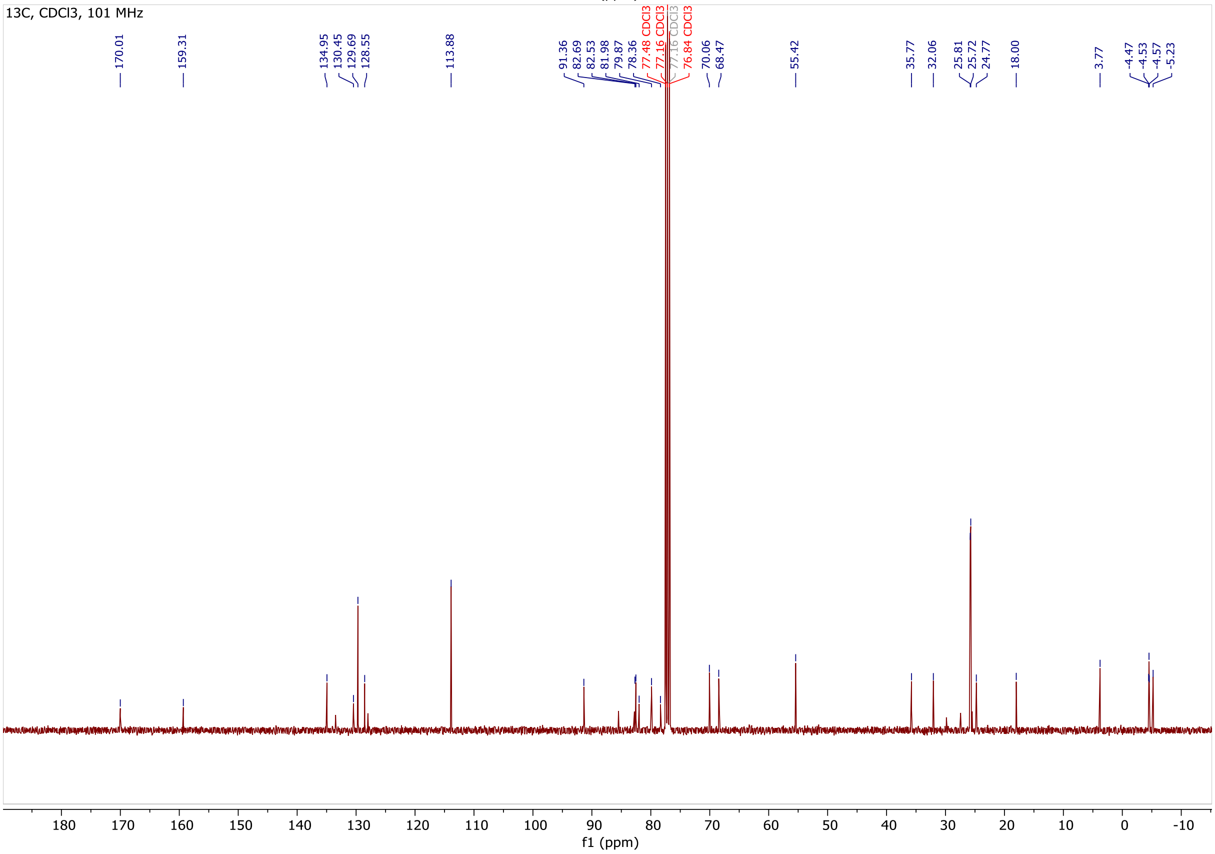

Compound 20 (*E*:*Z* ≈ 6:1)

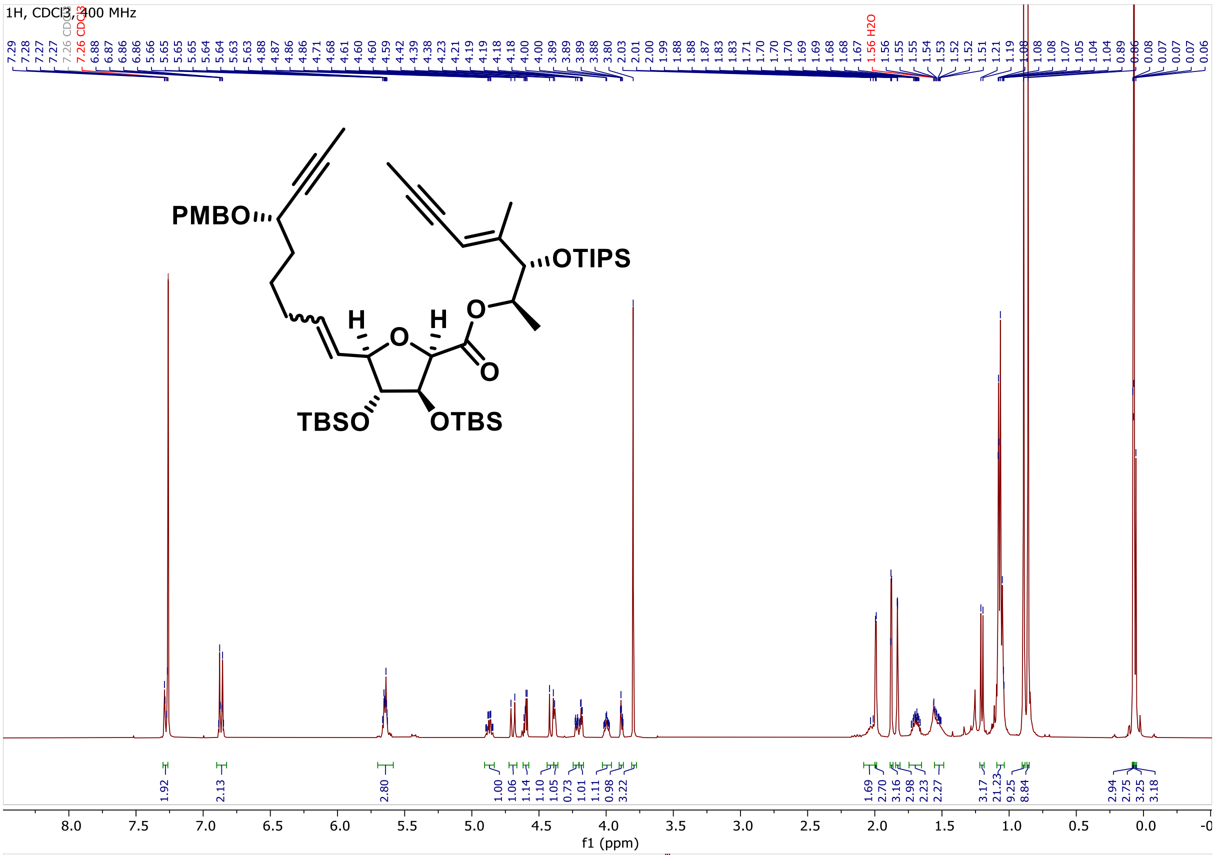

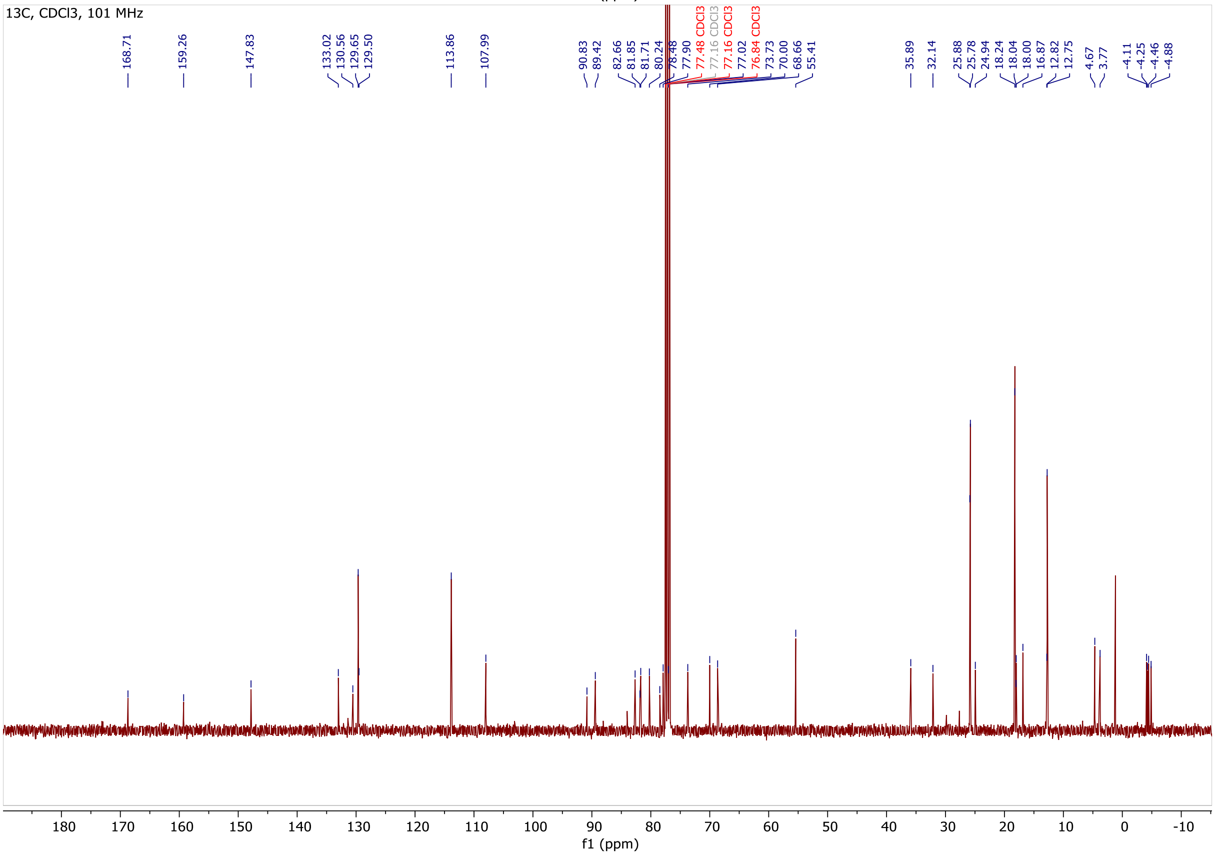

Compound 11-*epi*-20 (*E*:*Z* ≈ 8:1)

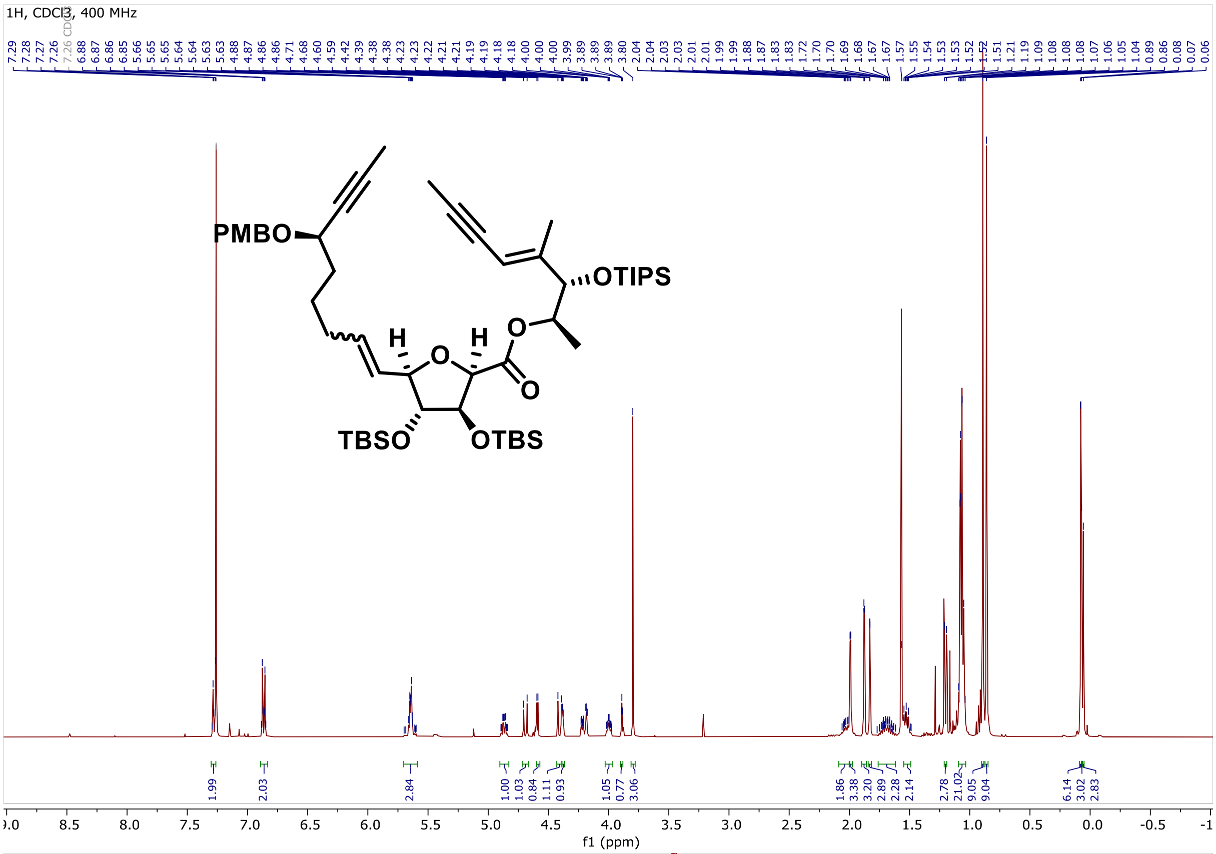

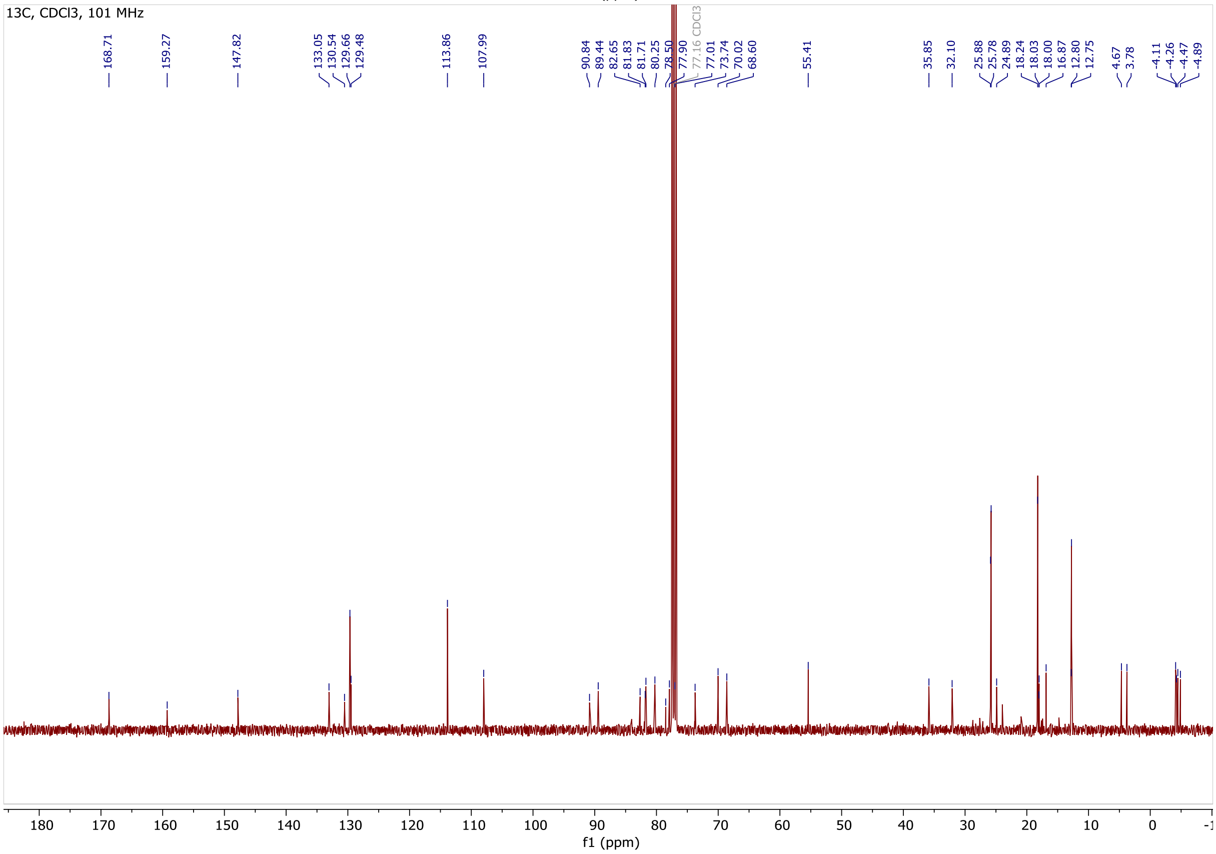

Compound 21

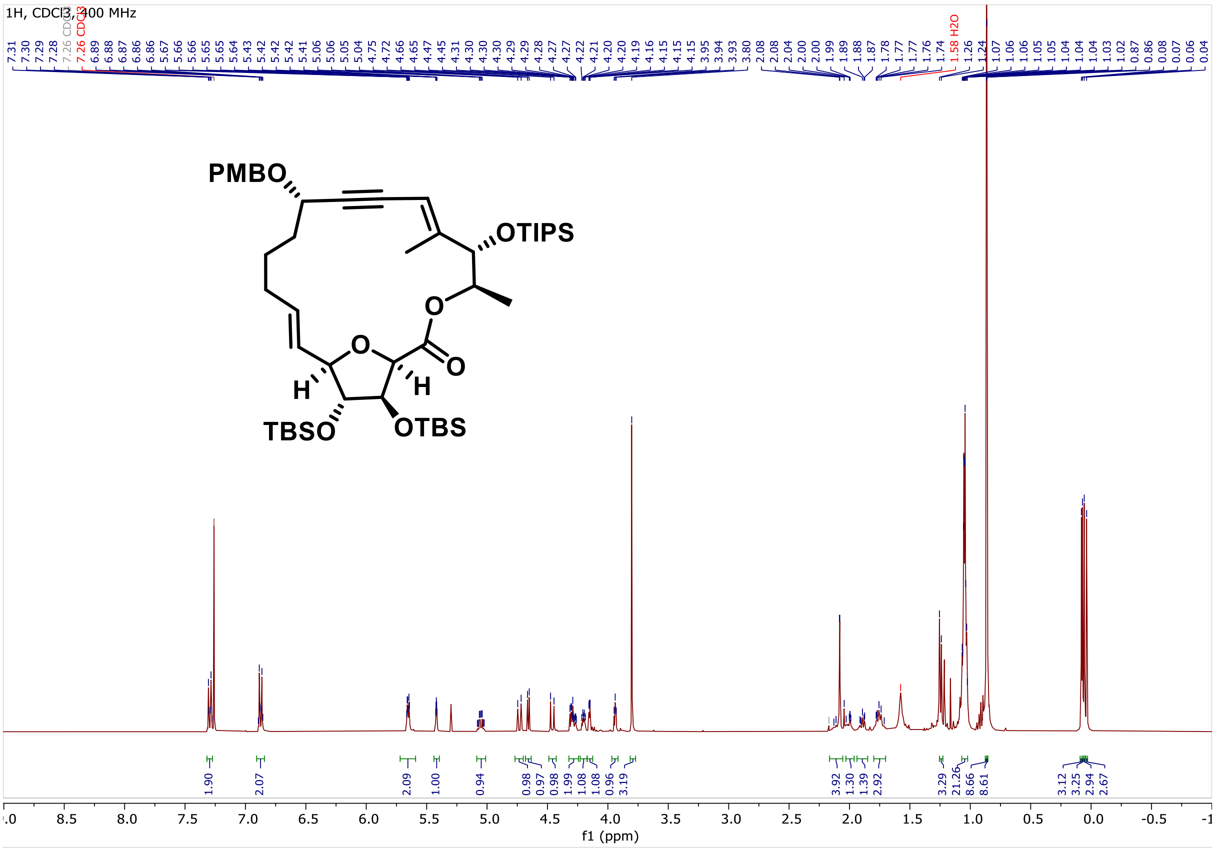

1H, CDCl3, 400 MHz
7.31 7.30 7.29 7.28 7.26 CDCl3 7.26 CDCl3 6.89 6.88 6.87 6.86 6.86 5.67 5.66 5.66 5.65 5.65 5.64 5.43 5.42 5.42 5.41 5.06 5.06 5.05 5.04 4.75 4.72 4.66 4.65 4.47 4.45 4.31 4.30 4.30 4.30 4.29 4.28 4.27 4.22 4.21 4.20 4.20 4.19 4.16 4.15 4.15 4.15 3.95 3.94 3.93 3.80 2.08 2.08 2.04 2.00 2.00 1.99 1.89 1.88 1.87 1.78 1.77 1.77 1.76 1.74 1.58 H2O 1.26 1.24 1.07 1.06 1.06 1.05 1.05 1.04 1.04 1.04 1.03 1.02 0.87 0.86 0.08 0.07 0.06 0.04
PMBO
OTIPS
O
O
O
H
H
TBSO
OTBS
1.90 2.07 2.09 1.00 0.94 0.98 0.97 0.98 1.99 1.08 1.08 0.96 3.19 3.92 1.30 1.39 2.92 3.29 21.26 8.66 8.61 3.12 3.25 2.94 2.67
8.5 8.0 7.5 7.0 6.5 6.0 5.5 5.0 4.5 4.0 3.5 3.0 2.5 2.0 1.5 1.0 0.5 0.0 -0.5
f1 (ppm)

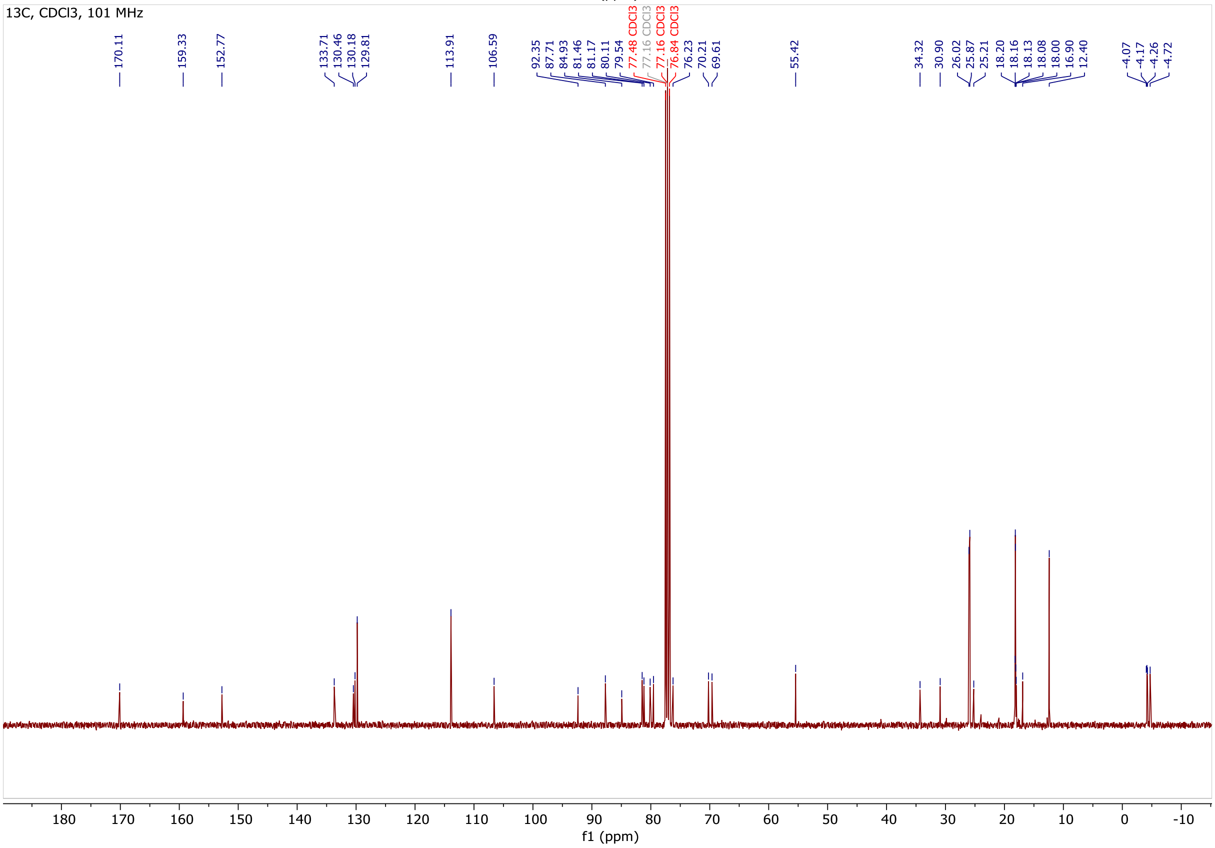

13C, CDCl3, 101 MHz
170.11 159.33 152.77 133.71 130.46 130.18 129.81 113.91 106.59 92.35 87.71 84.93 81.46 81.17 80.11 79.54 77.48 CDCl3 77.16 CDCl3 76.84 CDCl3 76.23 70.21 69.61 55.42 34.32 30.90 26.02 25.87 25.21 18.20 18.16 18.13 18.08 18.00 16.90 12.40 -4.07 -4.17 -4.26 -4.72
180 170 160 150 140 130 120 110 100 90 80 70 60 50 40 30 20 10 0 -10
f1 (ppm)

Compound S8 (Z-isomer)

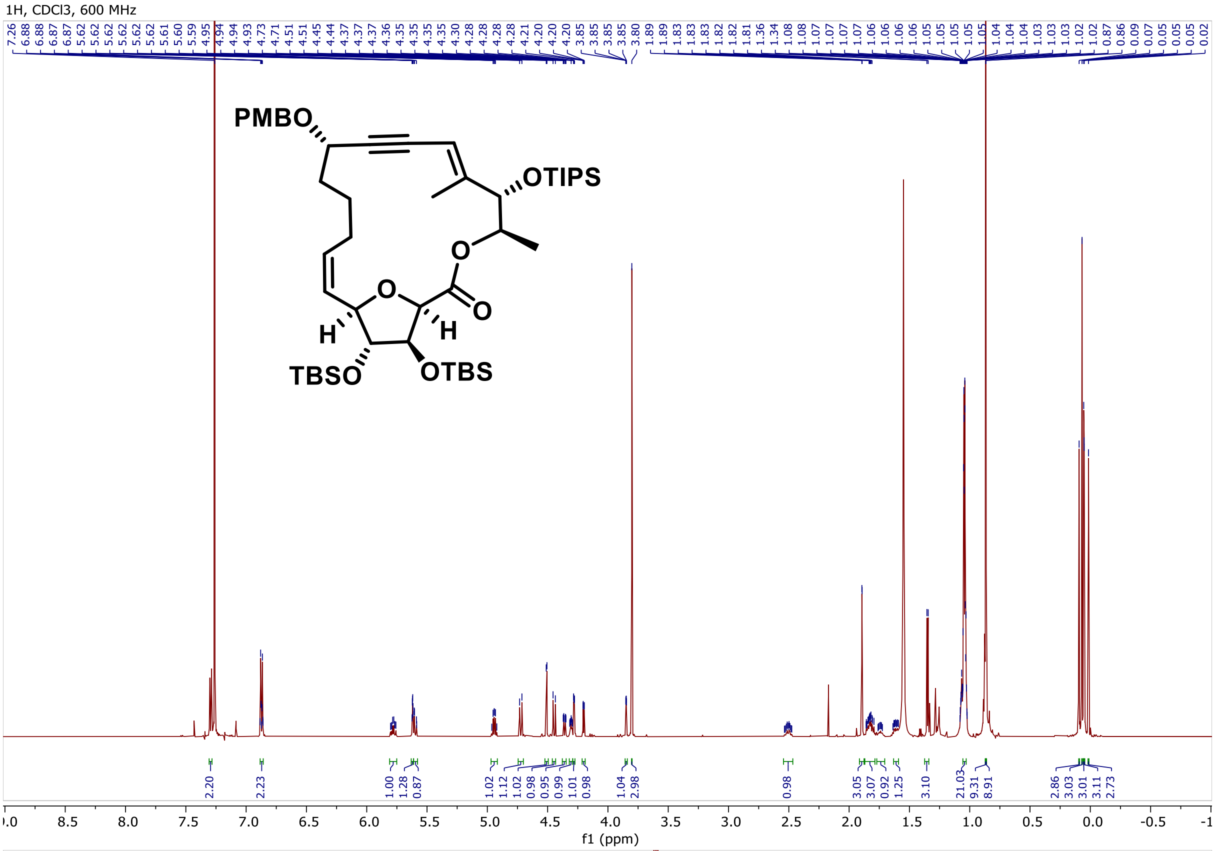

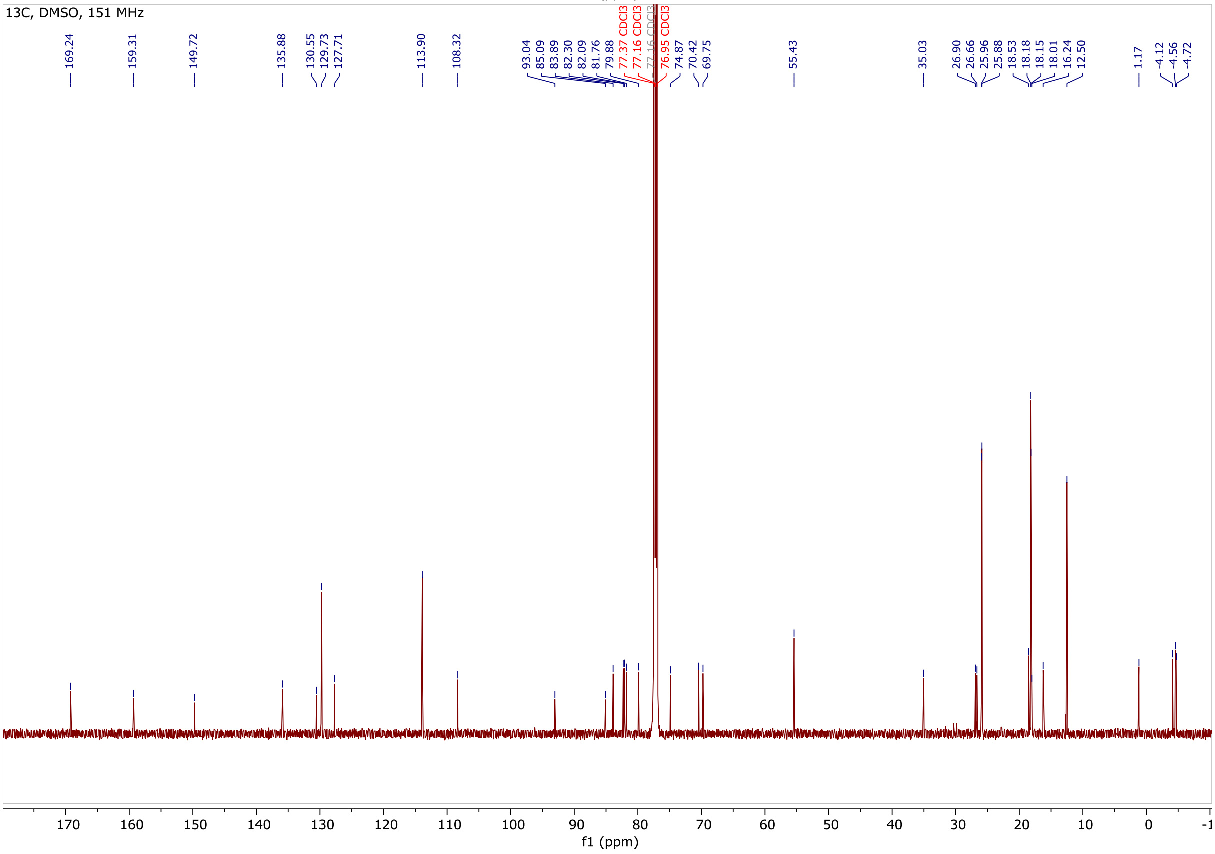

Compound 11-*epi*-21

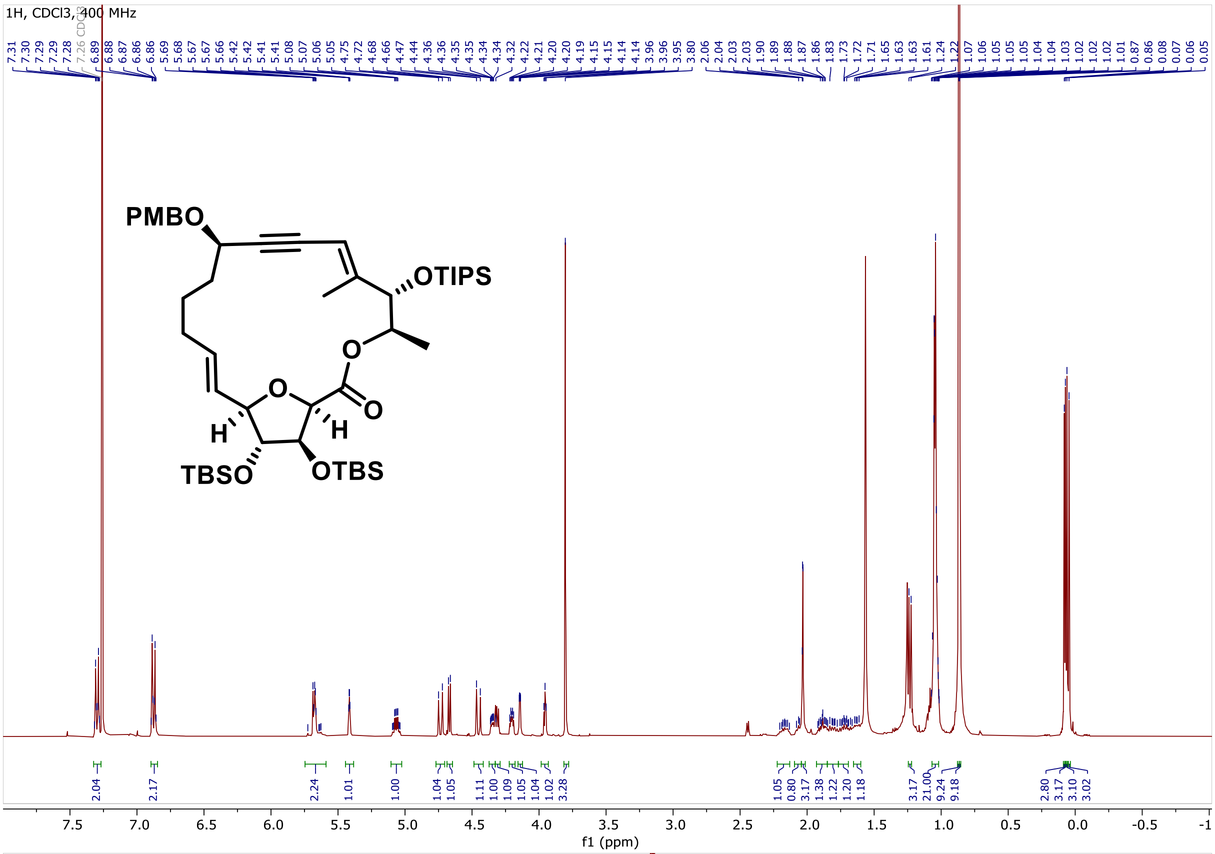

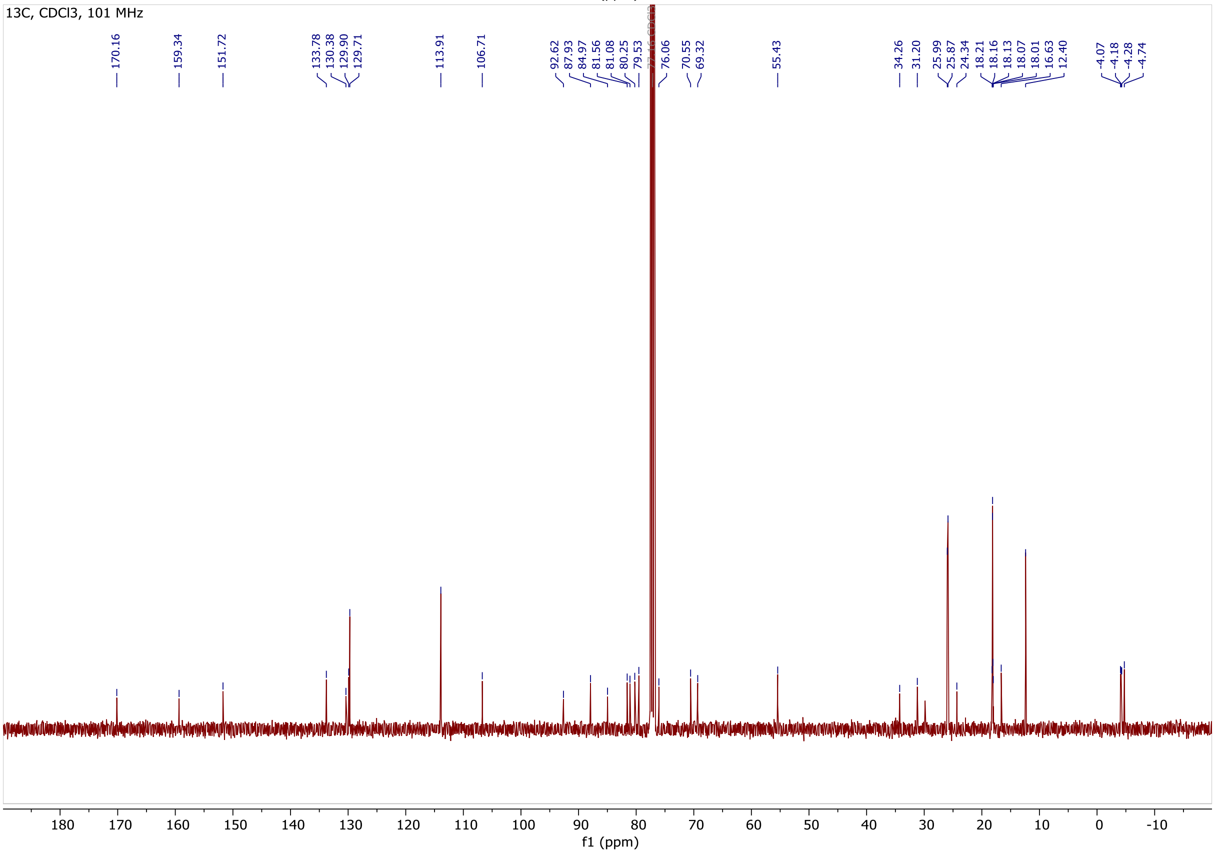

Compund 22 (containing trace of silicon grease)

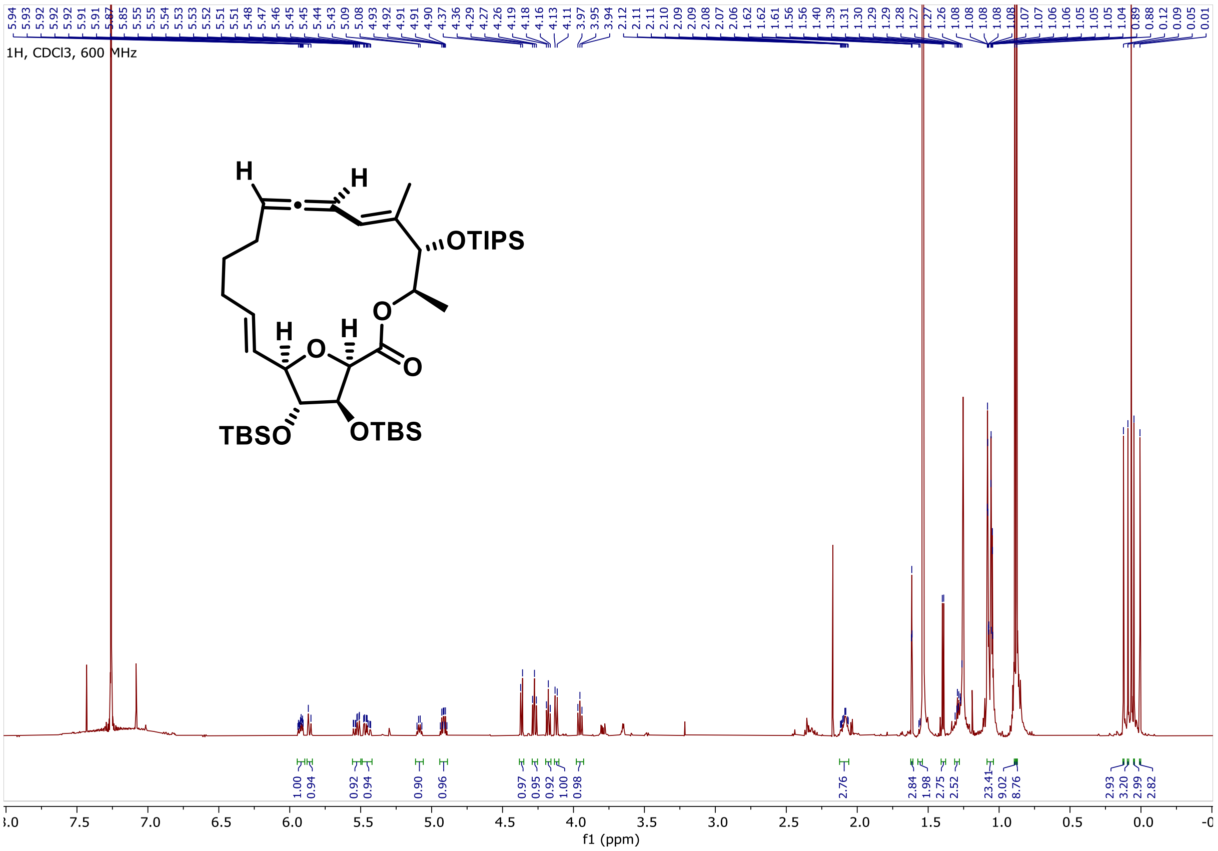

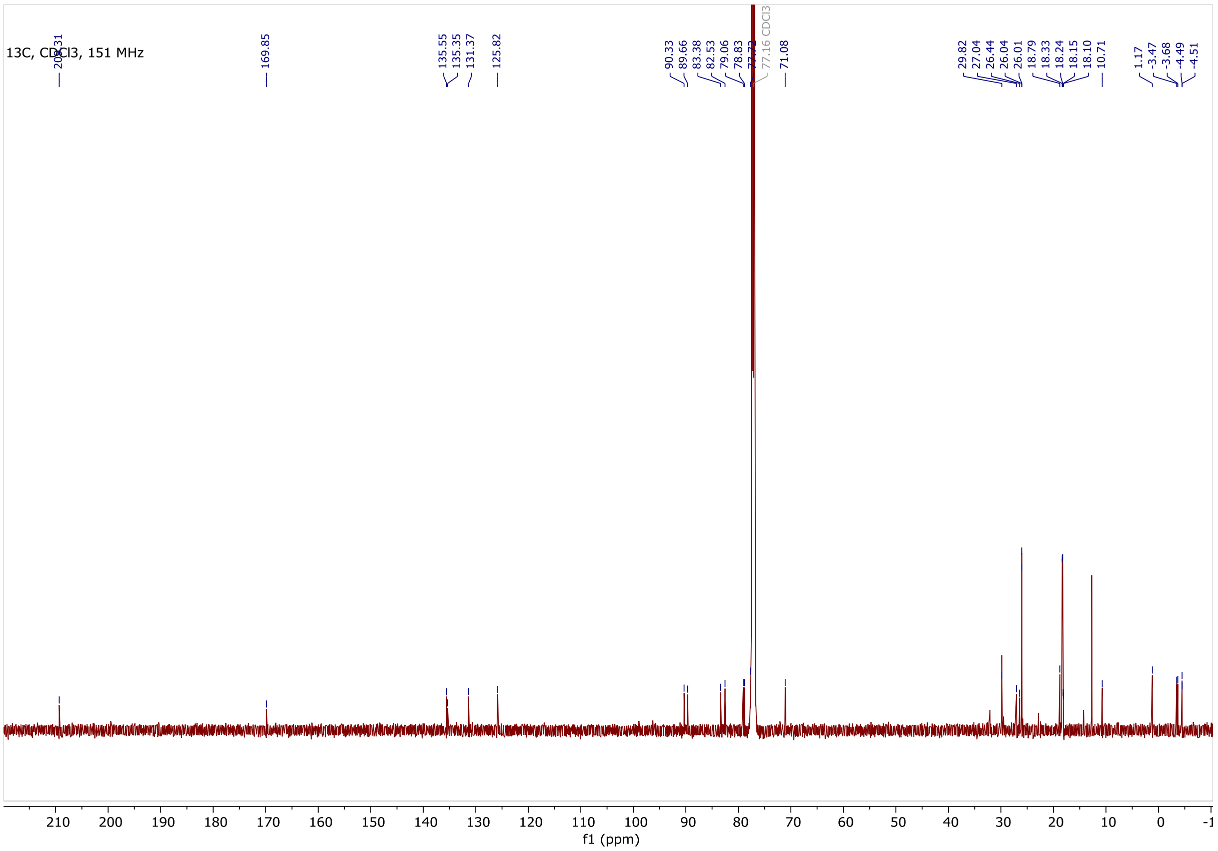

Compound 23

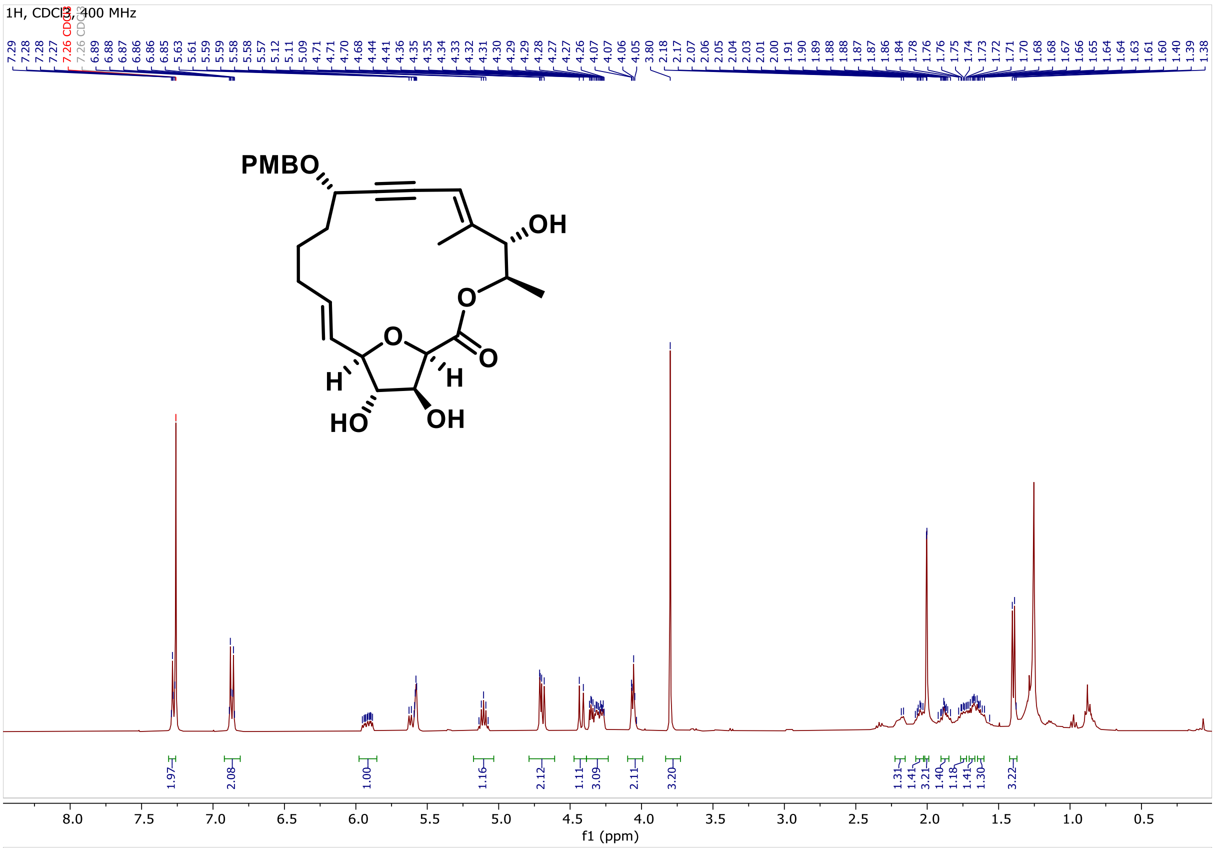
1H, CDCl3, 400 MHz
7.29 7.28 7.28 7.27 7.26 CDCl3 7.26 CDCl3 6.89 6.88 6.87 6.86 6.86 6.85 5.63 5.61 5.59 5.59 5.58 5.58 5.57 5.12 5.11 5.09 4.71 4.71 4.70 4.68 4.44 4.41 4.36 4.35 4.34 4.33 4.32 4.31 4.30 4.29 4.29 4.28 4.27 4.27 4.26 4.07 4.07 4.06 4.05 3.80 2.18 2.17 2.07 2.06 2.05 2.04 2.03 2.01 2.00 1.91 1.90 1.89 1.88 1.88 1.87 1.87 1.86 1.84 1.78 1.76 1.76 1.75 1.74 1.73 1.72 1.71 1.70 1.68 1.68 1.67 1.66 1.65 1.64 1.64 1.63 1.61 1.60 1.40 1.39 1.38
PMBO
OH
O
O
O
H
H
HO
OH
1.97 2.08 1.00 1.16 2.12 1.11 3.09 2.11 3.20 1.31 1.41 3.21 1.40 1.18 1.41 1.30 3.22
8.0 7.5 7.0 6.5 6.0 5.5 5.0 4.5 4.0 3.5 3.0 2.5 2.0 1.5 1.0 0.5
f1 (ppm)

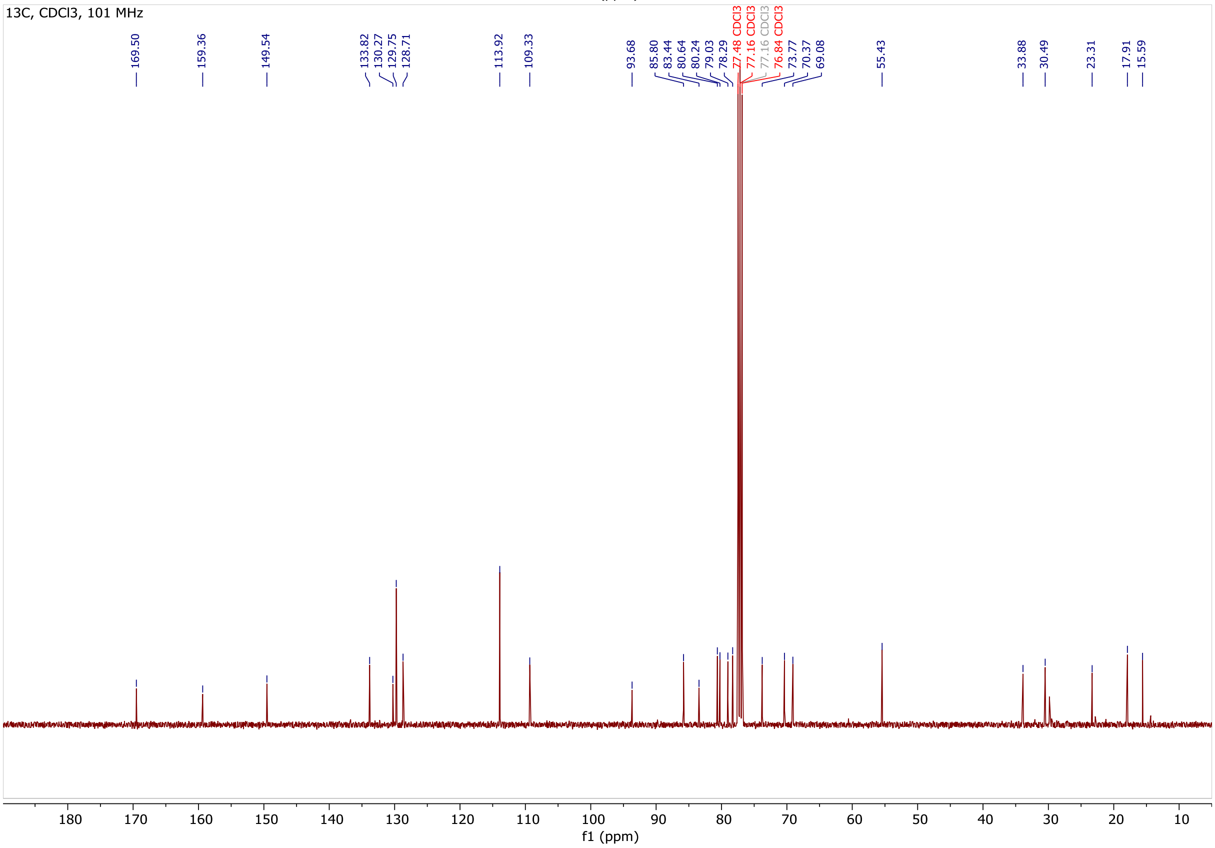
13C, CDCl3, 101 MHz
169.50 159.36 149.54 133.82 130.27 129.75 128.71 113.92 109.33 93.68 85.80 83.44 80.64 80.24 79.03 78.29 77.48 CDCl3 77.16 CDCl3 77.16 CDCl3 76.84 CDCl3 73.77 70.37 69.08 55.43 33.88 30.49 23.31 17.91 15.59
180 170 160 150 140 130 120 110 100 90 80 70 60 50 40 30 20 10
f1 (ppm)

Compound 11-*epi*-23

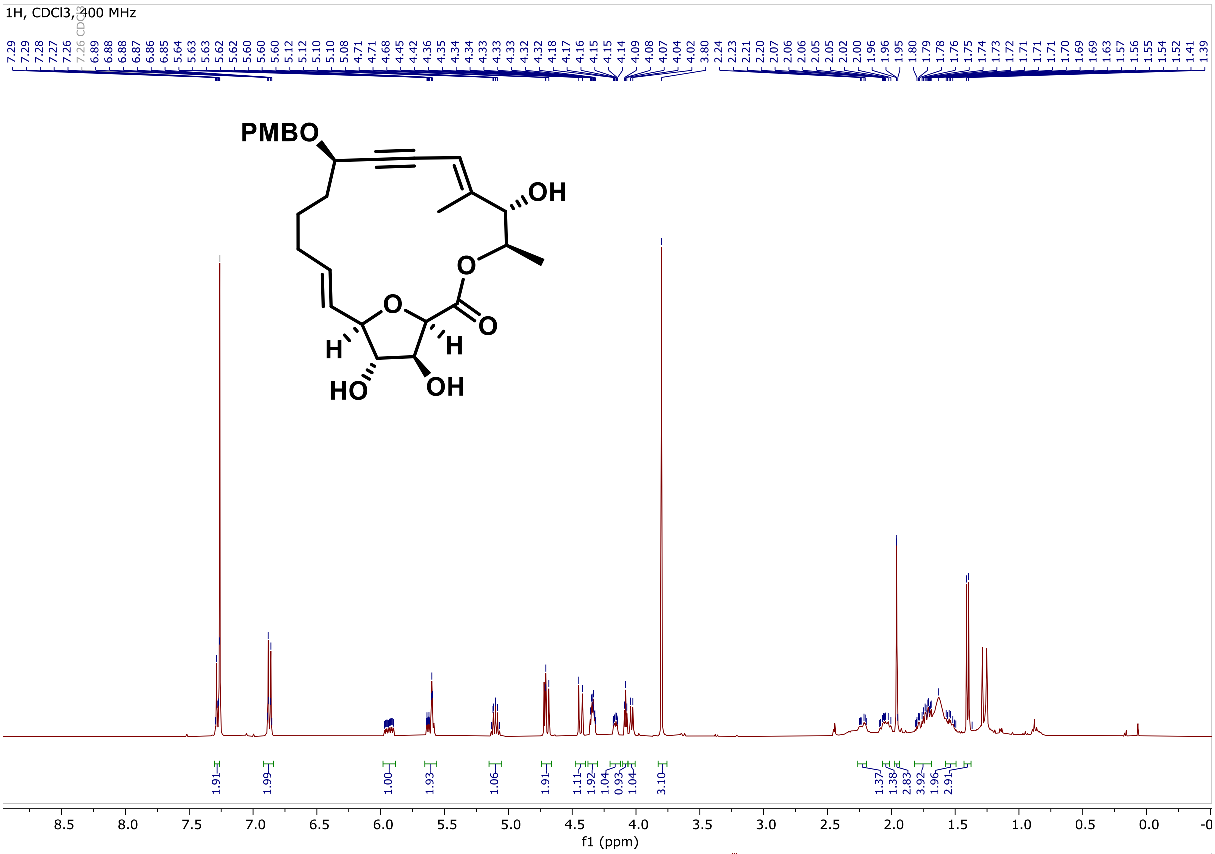

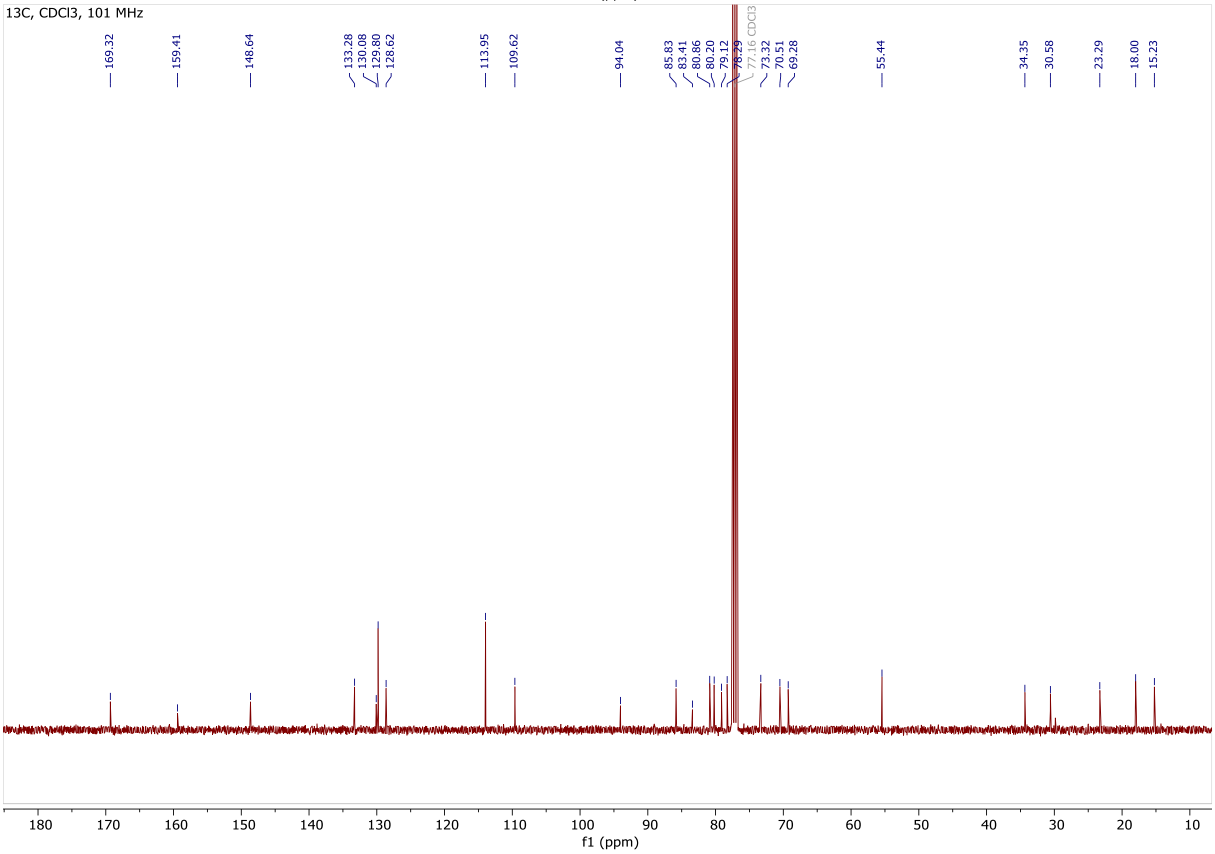

Archangiumide (1)

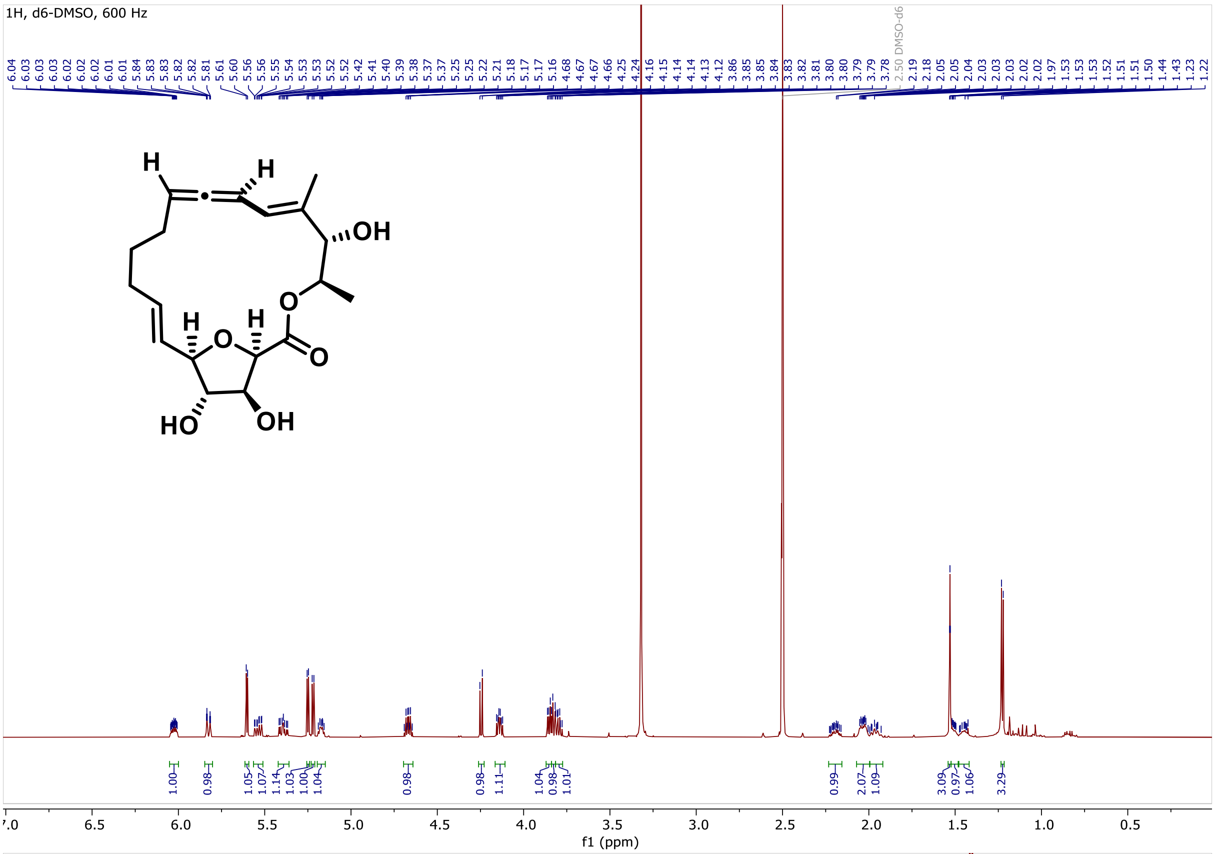

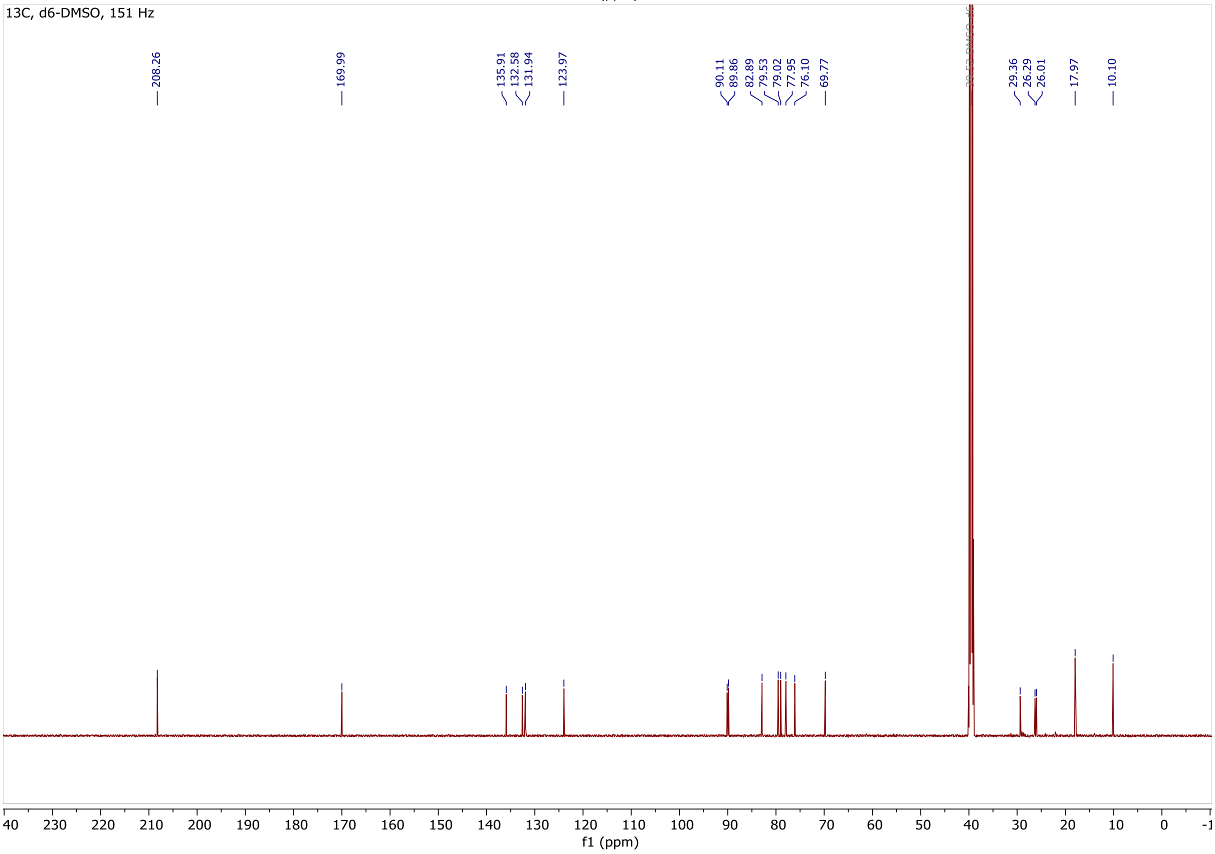

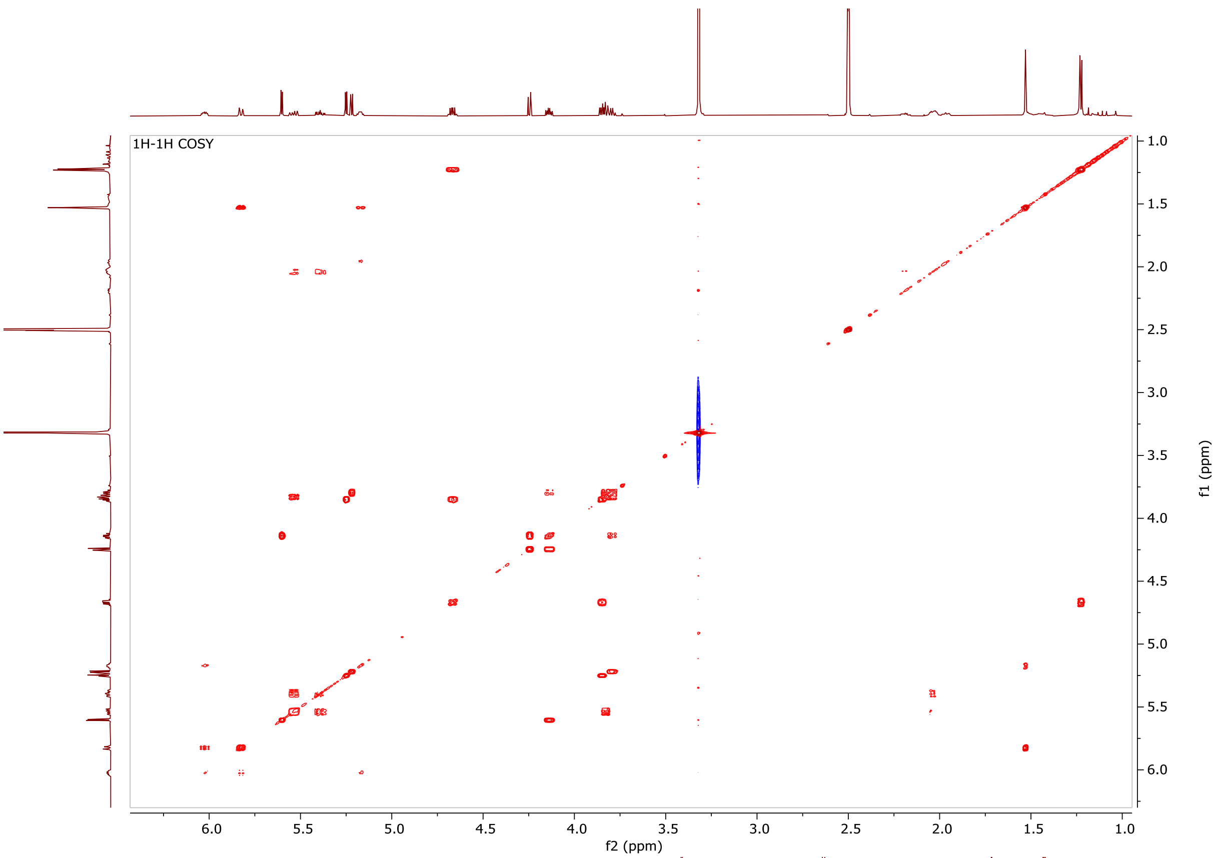

1H-1H COSY
6.0
5.5
5.0
4.5
4.0
3.5
3.0
2.5
2.0
1.5
1.0
f2 (ppm)
f1 (ppm)

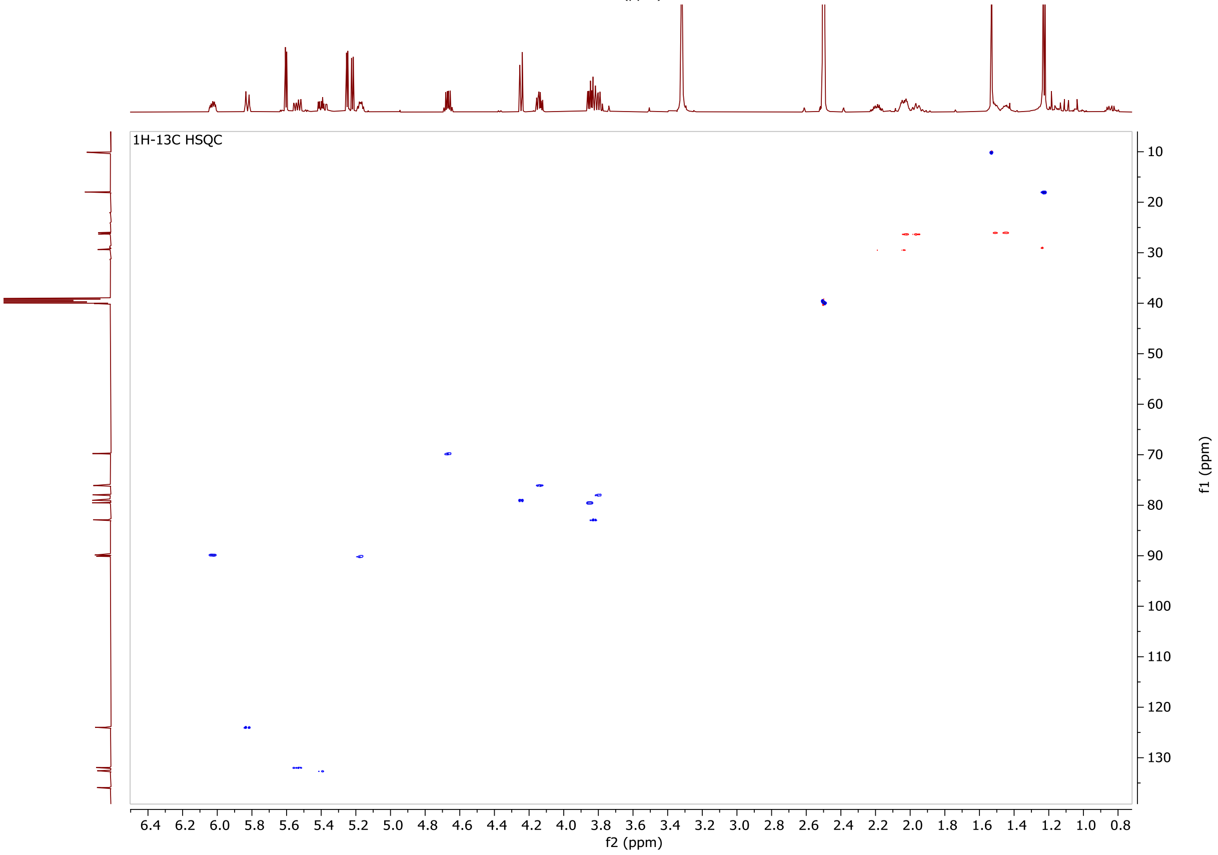

1H-13C HSQC
6.4
6.2
6.0
5.8
5.6
5.4
5.2
5.0
4.8
4.6
4.4
4.2
4.0
3.8
3.6
3.4
3.2
3.0
2.8
2.6
2.4
2.2
2.0
1.8
1.6
1.4
1.2
1.0
0.8
f2 (ppm)
10
20
30
40
50
60
70
80
90
100
110
120
130
f1 (ppm)

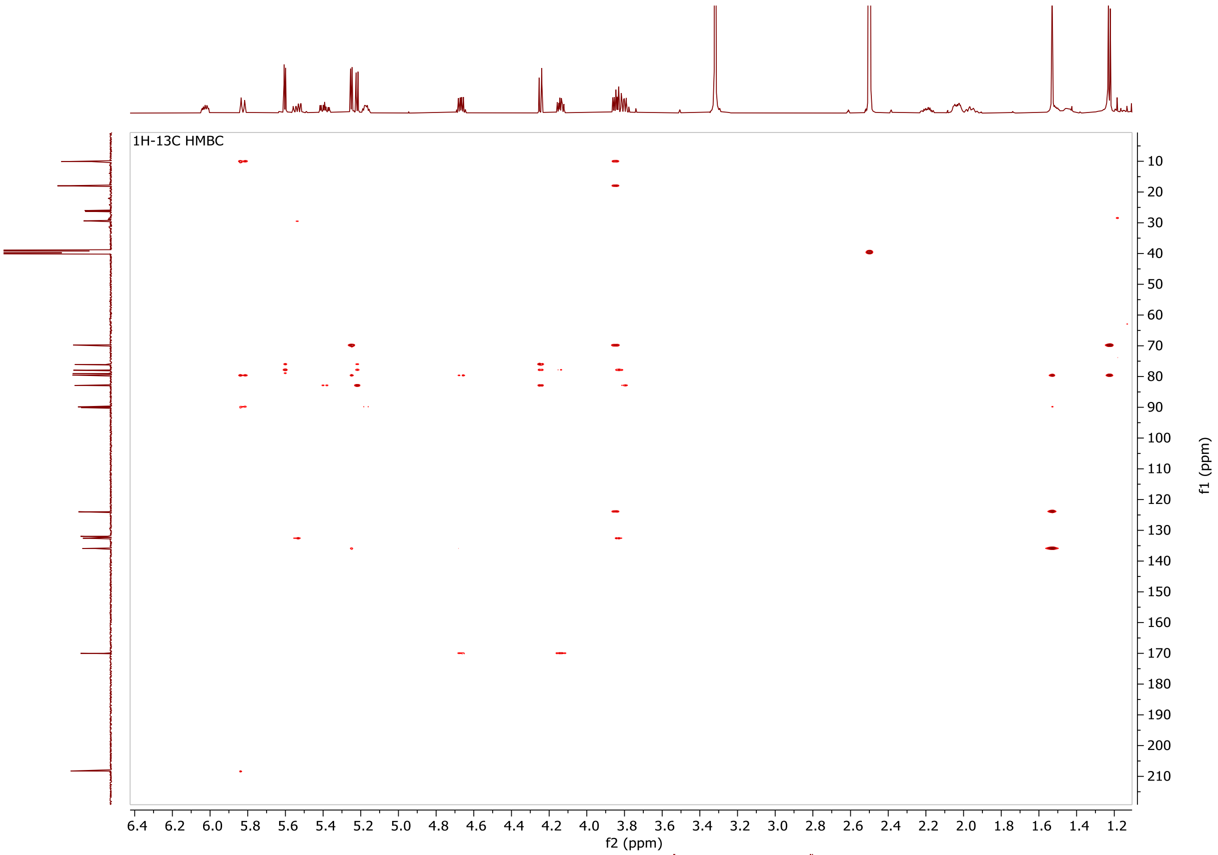
1H-13C HMBC
f2 (ppm)
f1 (ppm)

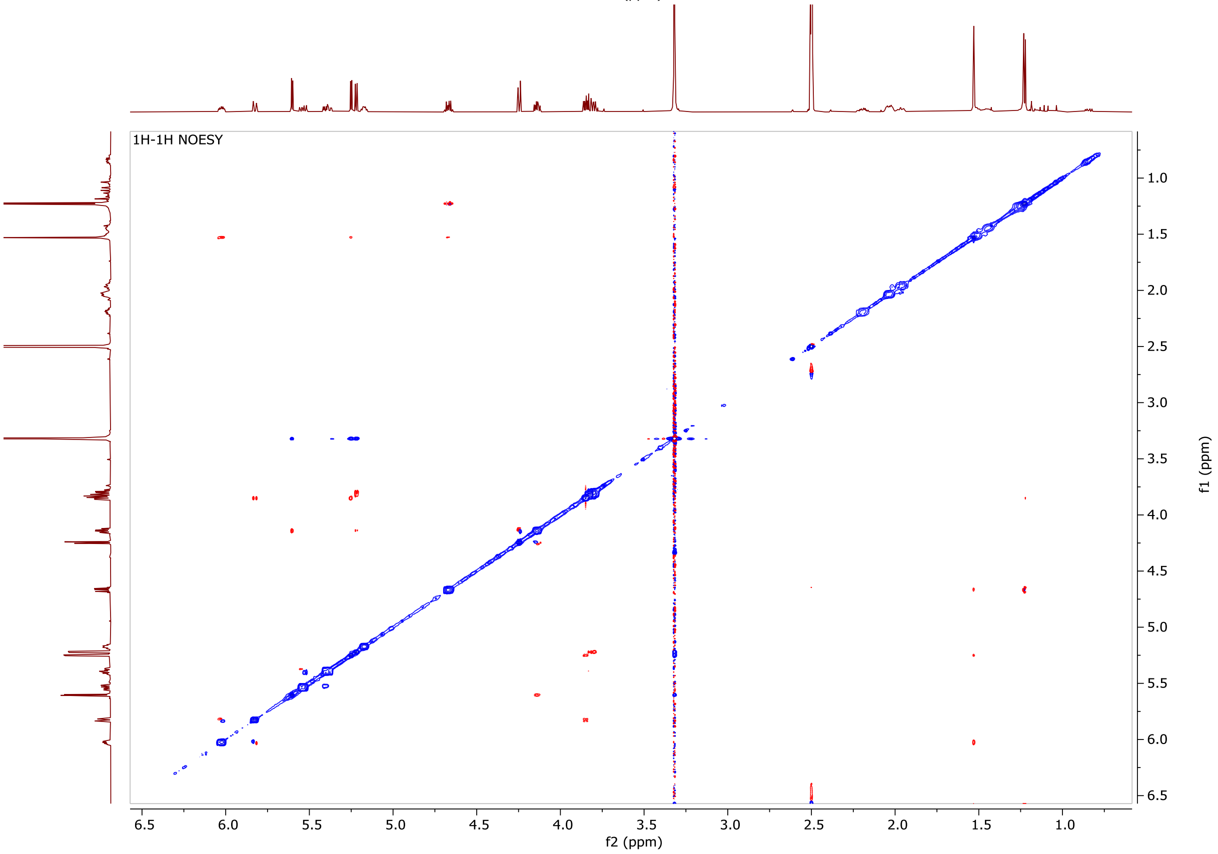
1H-1H NOESY
f2 (ppm)
f1 (ppm)

Compound 27

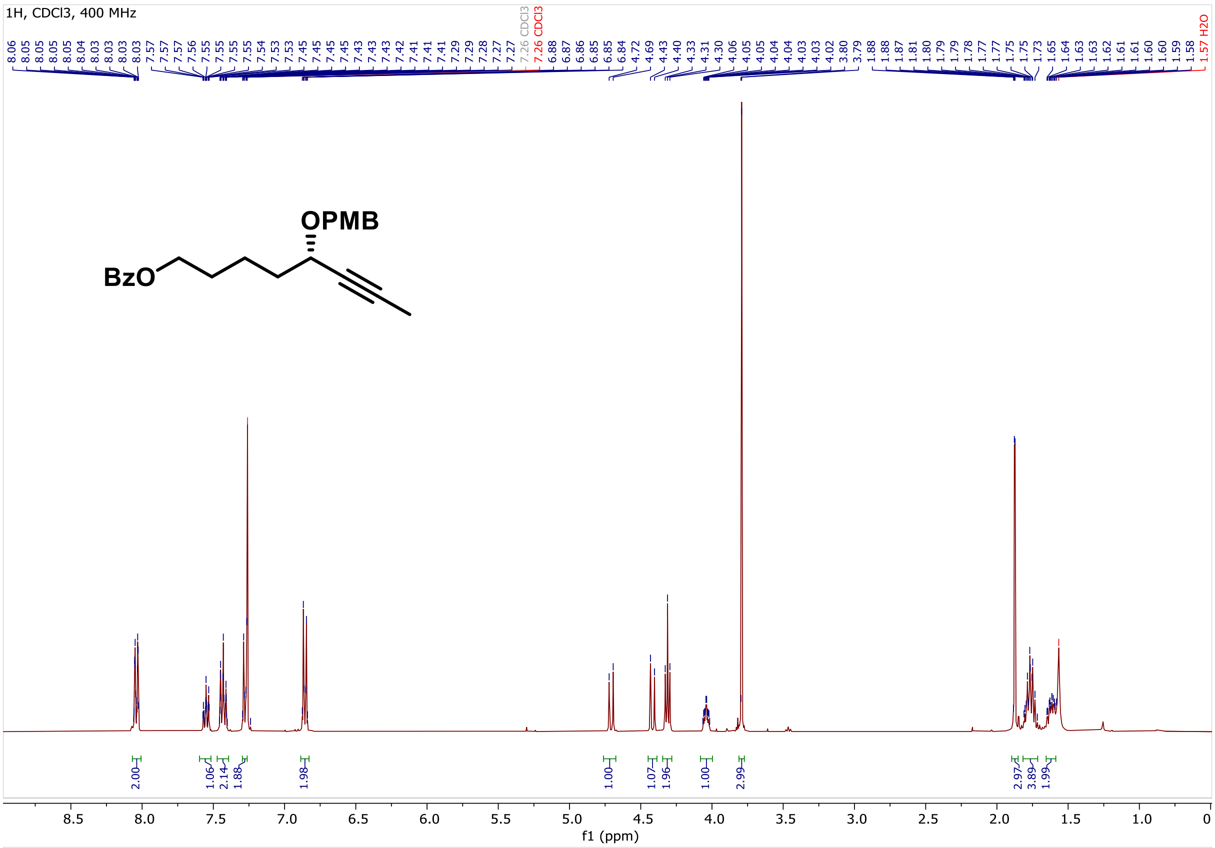
1H, CDCl3, 400 MHz
OPMB
BzO
f1 (ppm)

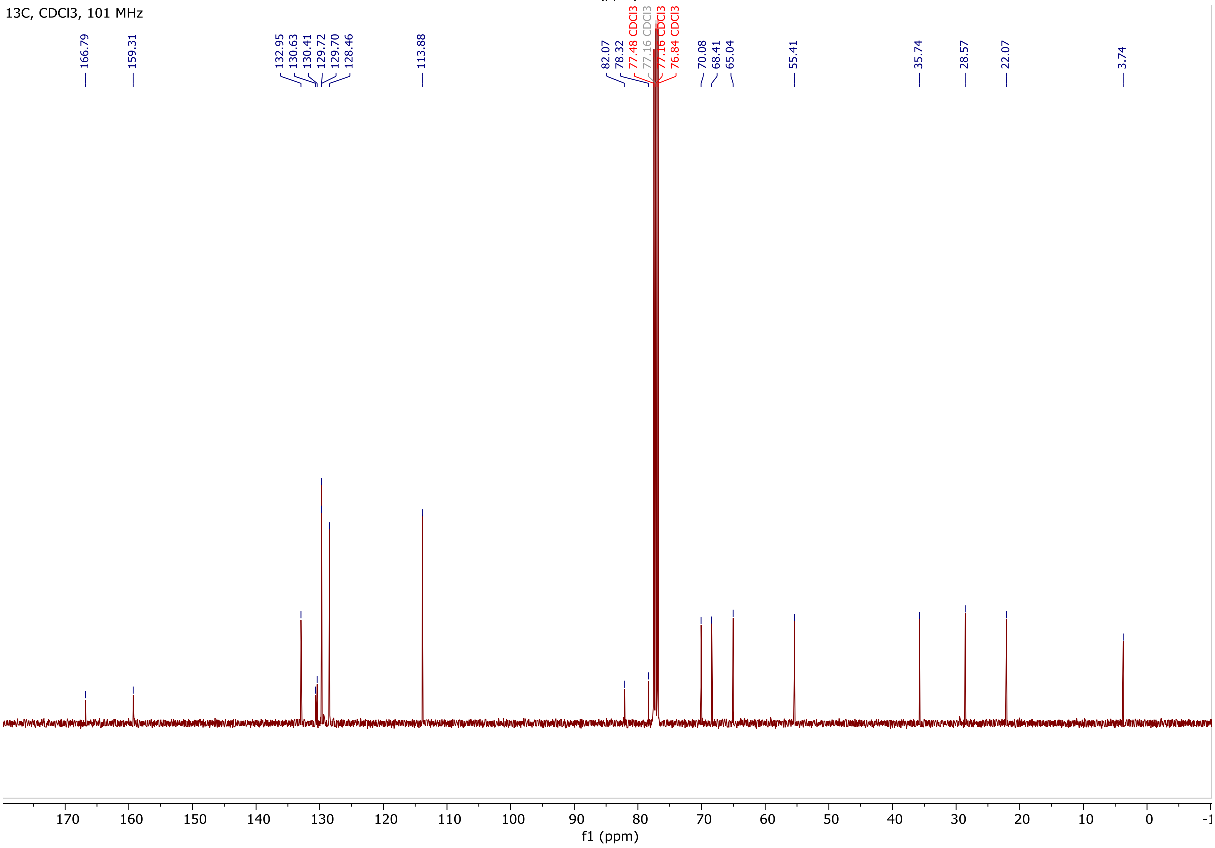
13C, CDCl3, 101 MHz
166.79
159.31
132.95
130.63
130.41
129.72
129.70
128.46
113.88
82.07
78.32
77.48 CDCl3
77.16 CDCl3
76.84 CDCl3
70.08
68.41
65.04
55.41
35.74
28.57
22.07
3.74
f1 (ppm)

Compound 28

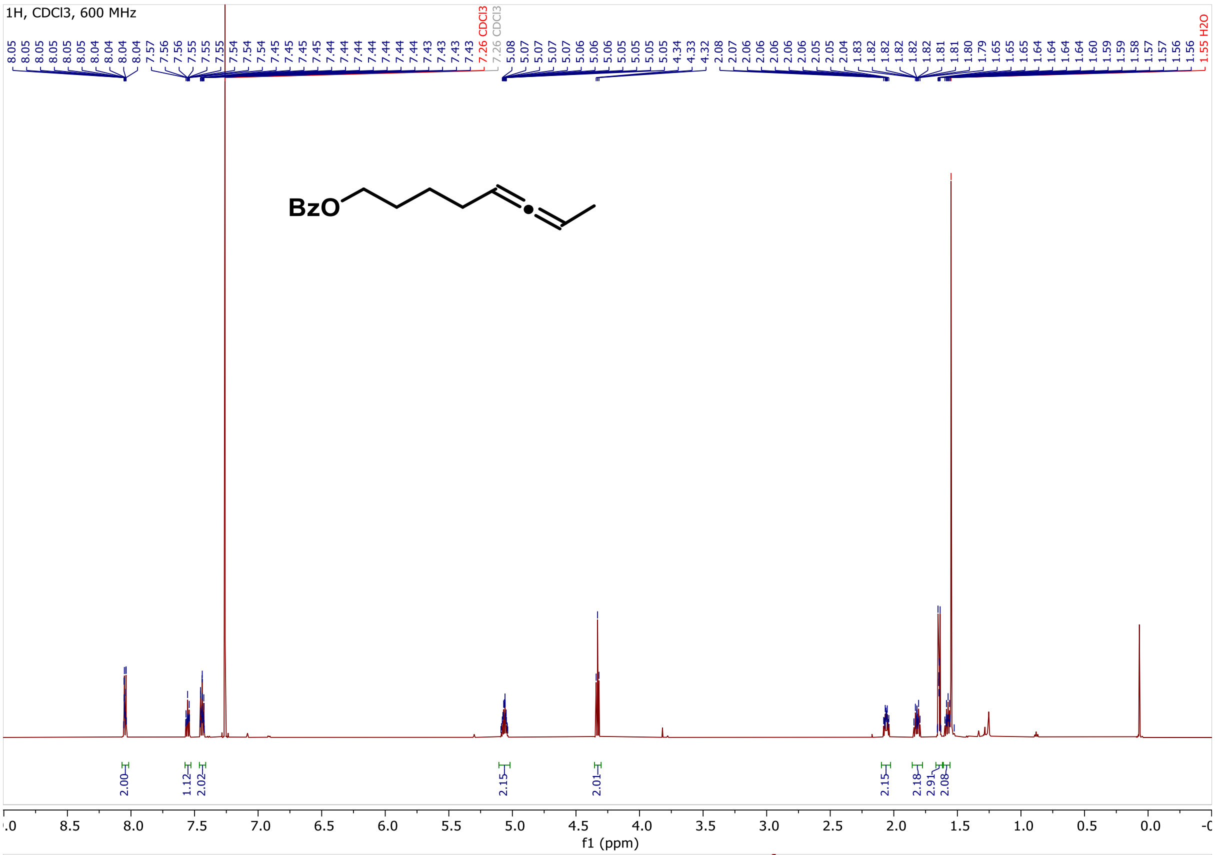
1H, CDCl3, 600 MHz
8.05 8.05 8.05 8.05 8.05 8.04 8.04 8.04 8.04 7.57 7.56 7.55 7.55 7.55 7.54 7.54 7.54 7.45 7.45 7.45 7.44 7.44 7.44 7.44 7.44 7.43 7.43 7.43 7.43 7.26 CDCl3 7.26 CDCl3 5.08 5.07 5.07 5.07 5.07 5.06 5.06 5.06 5.05 5.05 5.05 5.05 4.34 4.33 4.32 2.08 2.07 2.06 2.06 2.06 2.06 2.06 2.05 2.05 2.04 1.83 1.82 1.82 1.82 1.82 1.81 1.81 1.80 1.79 1.65 1.65 1.64 1.64 1.64 1.64 1.60 1.59 1.59 1.58 1.57 1.57 1.56 1.56 1.55 H2O
BzO
2.00 1.12 2.02 2.15 2.01 2.15 2.18 2.91 2.08
f1 (ppm)

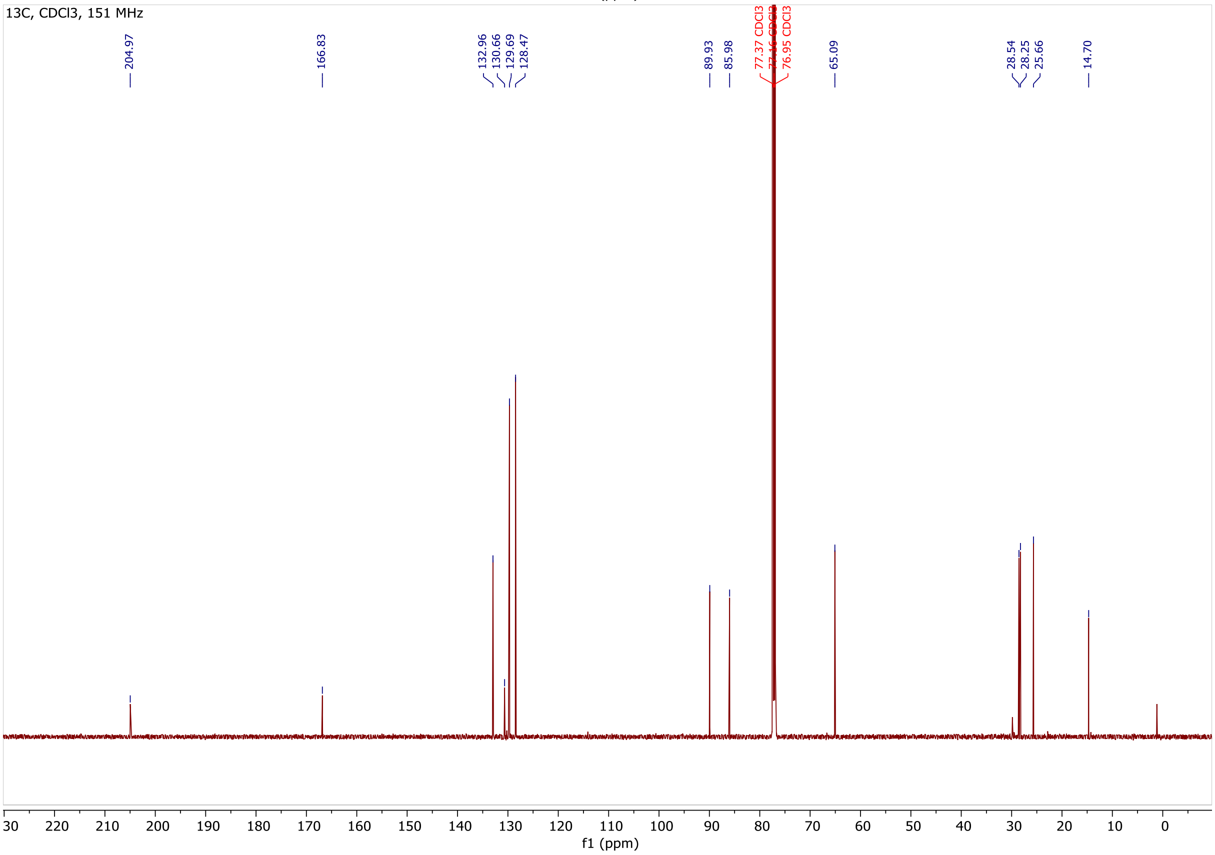
13C, CDCl3, 151 MHz
204.97
166.83
132.96 130.66 129.69 128.47
89.93
85.98
77.37 CDCl3 77.16 CDCl3 76.95 CDCl3
65.09
28.54 28.25 25.66
14.70
f1 (ppm)

References

1. Korber, J. N., Wille, C.; Leutzsch, M., Fürstner, A., *J. Am. Chem. Soc*. **2023**, (doi: 10.1021/jacs.3c10430).
2. Chabala, J. C., Vincent, J. E., *Tet. Lett.* **1978**, *19*, 937-940.
3. Haack, K.-J., Hashiguchi, S., Fujii, A., Ikariya, T., Noyori, R., *Angew. Chem. Int. Ed. Engl*. **1997**, *36*, 285-288.
4. Matsumara, K., Hashiguchi, S., Ikariya, T., Noyori, R., *J. Am. Chem. Soc.* **1997**, *119*, 8738-8739.
5. Hoye, T. R., Jeffrey, C. S., Shao, F., *Nat. Protocols* **2007**, *2*, 2451-2458.
6. Bolte, B., Odabachian, Y., Gagosz, F., *J. Am. Chem. Soc.* **2010**, *132*, 7294-7296.
7. Long, D. D., Smith, M. D., Martín, A., Wheatley, J. R., Watkin, D. G., Müller, M., Fleet, G. W. J., *J. Chem. Soc., Perkin Trans.* **2002**, 1982–1998.
8. Goodyear, E. H.; Haworth, W. N. *J. Chem. Soc*. **1927**, 3136.
9. Marshall, J. A., Xie, S., *J. Org. Chem.* **1995**, *60*, 7230-7237.
10. Cheng, X., Quintanilla, C. D., Zhang, L., *J. Org. Chem.* **2019**, *84*, 11054−11060.
11. Alfaro, R., Parra, A., Alemán, J., Ruano, J. L. G., Tortosa, M., *J. Am. Chem. Soc.* **2012**, *134*, 15165−15168.
12. Boucher, M. M., Furigay, M. H., Quach, P. K., Brindle, C. S., *Org. Process Res. Dev.* **2017**, *21*, 1394–1403.
13. Hu, J.-Q., Wang, J.-J., Li, Y.-L., Zhuo, L., Zhang, A., Sui, H.-Y., Li, X.-J., Shen, T., Yin, Y., Wu, Z.-H., Hu, W., Li, Y.-Z., Wu, C., *Org. Lett.* **2021**, *23*, 2114–2119.
